# High-risk clones of extended-spectrum β-lactamase-producing *Klebsiella pneumoniae* isolated from the University Hospital Establishment of Oran, Algeria (2011–2012)

**DOI:** 10.1371/journal.pone.0254805

**Published:** 2021-07-26

**Authors:** Assia Zemmour, Radia Dali-Yahia, Makaoui Maatallah, Nadjia Saidi-Ouahrani, Bouabdallah Rahmani, Nora Benhamouche, Hissa M. Al-Farsi, Christian G. Giske

**Affiliations:** 1 Faculté de Sciences de la Nature et la Vie, Département de Génétique Moléculaire Appliquée, Université des Sciences et la Technologie d’Oran Mohamed-Boudiaf USTOMB, Oran, Algérie; 2 Laboratoire de Génétique Médicale Appliquée à l’Ophtalmologie, Université d’Oran 1, Oran, Algérie; 3 Service de bactériologie, Etablissement Hospitalo-Universitaire 1er Novembre 1954, Oran, Algérie; 4 Faculté de médicine, Université d’Oran 1, Oran, Algérie; 5 Faculté de pharmacie de Monastir, Laboratoire d’Analyse, Traitement et Valorisation des Polluants de l’Environnement et des Produits (LATVPEP: LR01ES16), Université de Monastir, Monastir, Tunisie; 6 Faculté de Génie Electrique, Département d’Electronique, Université des Sciences et la Technologie d’Oran Mohamed-Boudiaf USTOMB, Oran, Algérie; 7 Division of Clinical Microbiology, Department of Laboratory Medicine, Karolinska Institutet, Stockholm, Sweden; 8 Central Public Health Laboratories, Ministry of Health, Muscat, Sultanate of Oman; 9 Clinical Microbiology, Karolinska University Hospital Solna, Stockholm, Sweden; Zhejiang University, CHINA

## Abstract

The purpose of the study was to characterize the resistome, virulome, mobilome and Clustered Regularly Interspaced Short Palindromic Repeats-associated (CRISPR-Cas) system of extended-spectrum β-lactamase producing *Klebsiella pneumoniae* (ESBL-KP) clinical isolates and to determine their phylogenetic relatedness. The isolates were from Algeria, isolated at the University Hospital Establishment of Oran, between 2011 and 2012. ESBL-KP isolates (n = 193) were screened for several antibiotic resistance genes (ARGs) using qPCR followed by Pulsed-Field Gel Electrophoresis (PFGE). Representative isolates were selected from PFGE clusters and subjected to whole-genome sequencing (WGS). Genomic characterization of the WGS data by studying prophages, CRISPR-Cas systems, Multi-Locus Sequence Typing (MLST), serotype, ARGs, virulence genes, plasmid replicons, and their pMLST. Phylogenetic and comparative genomic were done using core genome MLST and SNP-Based analysis. Generally, the ESBL-KP isolates were polyclonal. The whole genome sequences of nineteen isolates were taken of main PFGE clusters. Sixteen sequence types (ST) were found including high-risk clones ST14, ST23, ST37, and ST147. Serotypes K1 (n = 1), K2 (n = 2), K3 (n = 1), K31 (n = 1), K62 (n = 1), and K151 (n = 1) are associated with hyper-virulence. CRISPR-Cas system was found in 47.4%, typed I-E and I-E*. About ARGs, from 193 ESBL-KP, the majority of strains were multidrug-resistant, the CTX-M-1 enzyme was predominant (99%) and the prevalence of plasmid-mediated quinolone resistance (PMQR) genes was high with *aac(6′)-lb-cr* (72.5%) and *qnr’s* (65.8%). From 19 sequenced isolates we identified ESBL, AmpC, and carbapenemase genes: *bla*_*CTX-M-15*_ (n = 19), *bla*_*OXA-48*_ (n = 1), *bla*_*CMY-2*_ (n = 2), and *bla*_*CMY-16*_ (n = 2), as well as non-ESBL genes: *qnrB1* (n = 12), *qnrS1* (n = 1) and *armA* (n = 2). We found IncF, IncN, IncL/M, IncA/C2, and Col replicon types, at least once per isolate. This study is the first to report *qnrS* in ESBL-KP in Algeria. Our analysis shows the concerning co-existence of virulence and resistance genes and would support that genomic surveillance should be a high priority in the hospital environment.

## Introduction

*Klebsiella pneumoniae* (KP) is notorious for its great propensity to acquire antimicrobial resistance and confer nosocomial infections. This species is associated with the emergence of strains which are both hypervirulent and multidrug-resistant (MDR) [1]. KP frequently produce extended-spectrum β-lactamases (ESBLs), bacterial enzymes that can hydrolyze penicillins, cephalosporins, monobactams, and to some extent carbapenems [2,3]. Multidrug-resistant Gram-negative bacilli are the cause of high incidence rates of nosocomial infections worldwide [4]. The mobile genetic elements (MGEs) (plasmids, bacteriophages, and transposons) are capable of horizontal gene transfer (HGT) and hence are potential agents for the spread of virulence and/or antibiotic resistance genes (ARGs) [5,6].

In addition to ARGs and pathogenicity the prokaryotes, most archaea, and many bacteria encode the Clustered Regularly Interspaced Short Palindromic Repeats-associated genes (CRISPR-Cas) systems conferring an adaptive immunity against MGEs [7,8]; another form of acquired resistance, to MGEs. The CRISPR-Cas system is composed of CRISPR-loci (unique spacers and direct repeats (DRs) alternately), *cas* genes, and the leader sequence is an AT-rich sequence located upstream of the first repeat promotes transcription of the CRISPR locus [9–11]. Concerning, the classification of KP CRISPR-Cas system, two subtypes have been reported; I-E with one locus and I-E* with two loci up and downstream *cas* genes [12].

The Algerian antimicrobial resistance surveillance reported that the percentage of KP-ESBL was the highest among ESBL-producing Enterobacteriaceae during 2011, 2012, and 2013 with 57.7%, 60.7%, and 57.3%, respectively [13,14]. Consequently, ESBL-KP is considered as one of the most concerning antimicrobial resistance threats in Algeria.

The aims herein were to characterize ARGs, virulence genes, CRISPR systems, prophages, and clonal structure of clinical isolates KP-ESBL producing from the University Hospital Establishment, Oran, Algeria (EHUO).

## Materials and methods

### Ethical statement

The isolates were received from the Bacteriology laboratory of the University Hospital Establishment of Oran. The hospital routinely stores the ESBL-producing Enterobacteriaceae for further studies. The strains were collected for clinical diagnosis, and no additional sampling was required. A permit was obtained from the Ministry of Health in Algeria (referenced record: 114/Bac/EHU/2014) to transport and characterize the bacterial isolates and related clinical data via anonymization of the patients involved.

### Bacterial isolates and antimicrobial susceptibility

Initially, at the EHUO the strains were identified phenotypically by API20E® (bioMérieux-Marcy l’Etoile-France) and double disk synergy technique as KP-ESBL [15]. The antimicrobial susceptibility testing of bacteria was performed mainly by the disk diffusion method according to the Clinical and Laboratory Standards Institute (CLSI) guidelines [16]. Susceptibility testing was performed against different discs of antibiotics [ampicillin (10μg), ticarcillin (75μg), amoxicillin/clavulanic acid (20/10μg), cephalothin (30μg), cefazolin (30μg), ceftazidime (30μg), cefpirome (30μg), cefotaxime (30μg), cefepime (30μg), cefoxitin (30μg), aztreonam (30μg), ertapenem (10μg), imipenem (10μg), meropenem (10μg), amikacin (30μg), gentamicin (10μg), nalidixic acid (30μg), ciprofloxacin (5μg) pefloxacin (5μg), trimethoprim/sulfamethoxazole (10/20μg), fosfomycin (200μg), nitrofurantoin (300μg)]; The susceptibility to antibiotics was determined by the Mueller-Hinton (MH) agar diffusion method which was previously seeded with a pure culture of the strain to be studied from a 0.5 McFarland suspension and an incubation at 37°C for 18 hours, the control was the *E*. *coli* ATCC25922. Since 2012, the analysis was updated with several versions of CLSI guidelines, in this paper the 2020 criterion was used [16].

The reports showed that the percentage of KP-ESBL was the highest among ESBL-producing Enterobacteriaceae and associated with nosocomial infection in the EHUO. We received 219 KP-ESBL responsible for nosocomial infectious diseases, collected between January 2011 and December 2012 and isolated from different departments of the EHUO for further tests.

At Karolinska Institutet, the isolates were re-tested and re-identified using MALDI-TOF/MS (Microflex, MALDI Biotyper, BrukerDaltonics, Germany), the contaminated and non-viable strains were excluded. The European Committee on Antimicrobial Susceptibility Testing (EUCAST) guideline on detection of resistance mechanisms version 2.0 (July 2017) was used to select isolates that had a diameter of less than 27 mm with meropenem for the confirmation kit KPC/MBL and OXA-48 (ROSCO DIAGNOSTICA®, Taastrup, Denmark) [17].

### Molecular screening of ARGs

The extraction of the DNA by thermal shock method was carried out for all strains, four to five large colonies were taken, suspended in an Eppendorf tube containing 200 μL of molecular biology quality water (Sigma Aldrich), after homogenization (vortex) followed by heating at 95°C for 5 minutes, and centrifugation for 2 minutes. The total DNA was retained in the supernatant.

The characterization for all resistant genes was carried out by SYBR Green real-time PCR, using the Rotor Gene 3000 apparatus (Corbett Research, Australia). The analysis of the results was done with the Rotor Gene Real Time Analysis Software (Version 6.1). The same composition was used for the reaction mixture for all PCR-SYBR Green. A total volume of 25 μl of the reaction mixture contains 12.5 μl QuantiTect SYBR Green PCR Kit (Qiagen, Germany), 1.25 μl of each primer (10 μM), 7.5 μl of RNase-free water, and 2.5 μl DNA tested.

Amplification of the *bla*_*CTX-M*_ was done as described previously for all isolates [18]. CTX-M-positive isolates were screened for CTX-M-1, CTX-M-2, and CTX-M-9 groups (S1 Table) [19]. The thermal conditions were as follows: denaturation at 95°C for 15 mins, then 40 cycles of 30 s at 95°C, 1 min at 52°C and 1 min at 72°C, and a final extension of 65°C for 30 s. All PCRs were performed in the presence of appropriate positive control.

Plasmid*-*mediated quinolone resistance (PMQR) genes (*qnrA*, *qnrB*, *qnrC*, *qnrD*, *qnrS*, *qepA*, and *aac(6)–Ib*) was searched for in all strains (S1 Table) [20,21]. The thermal conditions for the amplification of the *qnrA*, *qnrB*, *qnrC*, *qnrD*, *qnrS*, and *qepA* genes were as follows: denaturation at 95°C for 15 mins, then 30 repetitions of 30 s at 95°C, 1 min at 52°C and 1 min at 72°C, and a final extension of 65°C for 30 s. Furthermore, the conditions of the PCR for *aac(6)-Ib* were as follows: denaturation at 95°C for 15 mins, then 45 cycles, 20 s at 95°C. 40 s at 59°C and 20 seconds at 70°C, and a final extension of 65°C for 30 s. PCRs were carried in presence of the following positive controls: *qnrB*, *qnrS*, *qepA*, and *aac(6)-Ib*. For *aac(6’)-Ib*, the positive PCR products were enzymatically digested by *BtsCl* (New England Biolabs, Ipswich, USA) and incubated at 50°C for 2 hours. Agarose gel 2%, E-Gel® (Invitrogen, USA) was used for the separation of digests.

Amikacin resistant strains were tested for the presence of 16S rRNA methylases genes; *armA*, *rmtA*, *rmtB*, *rmtC*, and *rmtD* (S1 Table), the primers were previously described elsewhere [22]. Using the following amplification conditions: denaturation at 95°C for 15 minutes and cycle repetitions: 30 s at 95°C and 1 min at 58°C and 1 min at 72°C, and a final extension at 65°C for 30 s. Positive controls for *armA*, *rmtB*, and *rmtD* were included in the PCR reaction.

Later, the Check MDR CT101 microarray (Checkpoints, The Netherlands) was used for strains that were negative in the CTX-M PCR and those which were resistant to cefoxitin for the detection of plasmid-mediated AmpC genes (pAmpC). The bacterial DNA extraction was done by the BioRobot M48 using the MagAttract DNA mini M48 kit (both from Qiagen, Hilden, Germany) according to the manufacturer’s instructions. The Nanodrop (Wilmington, Delaware USA) was used to check the DNA concentration. The results were analyzed by Check-Point Software (version 1.2).

### Pulsed-field gel electrophoresis

All isolates were typed using Pulsed-Field Gel Electrophoresis (PFGE) method. It was performed according to the PulseNet protocol for non-O157 *E*. *coli* using *XbaI* [23]. The band patterns were analyzed by GelCompar II version 5.10 (Applied Maths, Belgium). A dendrogram was generated based on Dice similarity and the Unweighted Pair-group Method with Arithmetic Averages (UPGMA). Closely related isolates were estimated according to the criteria established by Tenover *et al*. Dice co-efficient of ≥ 0.80 was considered the cut-off for possible clonal relatedness [24].

### Whole Genome Sequencing (WGS)

A total of nineteen isolates were taken for the WGS, seventeen were selected from the PFGE clusters, and two non-typeable isolates. The Genomic DNA was extracted using MagNApure 96 system (Roche Diagnostics, Nederland, Almere, Netherlands). The extracted DNA was quantified using the third generation Qubit 3.0 fluorometer (Life Technologies, Thermo Fisher Scientific Inc.) according to the manufacturer’s recommendations for the Qubit dsDNA HS Assay Kit (High Sensitivity). Sequencing was carried out on the HiSeq 2500 platform (Illumina®, San Diego, USA) at the Science for Life laboratory (SciLife, Solna, Sweden). Sequencing quality control showed that the 10x coverage was ˃98% for 19 isolates and the 30x coverage was ≥ 98% for 18 isolates. The main duplicate rate was ≥9.49% and the main median insert size between 186 and 267 (S2 Table). Raw reads data are available from the NCBI database (SRA) as project PRJNA672836 (accession no. SAMN16579464 to SAMN16579482).

## *In silico* analysis and annotation

The trimming and *de novo* assembly of the short paired reads were performed using CLC Genomics Workbench V 11.0, and the CLC Microbial Genomics Module 3.0 (Qiagen Bioinformatics, Aarhus, Denmark). The assembled genomes were mapped to the reference genome *K*. *pneumoniae* CG43 (GenBank accession number: NC_022566) and to call SNPs using the default settings.

The Center for Genomic Epidemiology database (https://cge.cbs.dtu.dk); ResFinder, PlasmidFinder, and pMLST were used [25,26]. CrisprCasFinder and CRISPRminer were utilized to characterize the CRISPR-Cas loci composed of operon organization, spacers and direct repeated (DRs), typing, self-targeting, and to map anti-CRISPR [9,27]. Basic Local Alignment Search Tool (megaBlast) was used only for non-duplicated spacers in several databases: virus (taxid: 10239), plasmids, and chromosomal bacterial genomes (taxid: 2). The leaders were searched by investing and blasting the regions upstream the CRISPR arrays (up to ~1kb) and using Bacterial promoter prediction program (Bprom) to predict the canonical core promoter elements (-10, -35 and the promoter sequence) of each CRISPR array [28]. PHAge Search Tool Enhanced Release (PHASTER) was used to identify putative prophages [29,30]. Kaptive V 0.7.0 was used to predict the K-type [31].

Assembled contigs have been deposited into the Bacterial Isolate Genome Sequence Database (BIGSdb) (http://bigsdb.pasteur.fr/klebsiella/klebsiella.html) and used to predict the virulence genes, the MLST scheme, and 632 loci core genome MLST (cgMLST632) scheme [32]. In this study, the isolates having three virulence gene clusters and more (high virulence factors) were considered as virulent strains while the isolates having less as classic strains (few virulence factors). The used MLST schemes: the first was based on the sequences of seven housekeeping genes (*gapA*, *infB*, *mdh*, *pgi*, *phoE*, *rpoB*, and *tonB*) and the second cgMLST632 was based on scanning the genomes against 632 loci. Annotated loci were saved in the form of allele numbers which allow the identification of allelic profiles of each isolate. The evolutionary relationship between isolates was assessed by Minimum Spanning Tree (MST), an algorithm implemented in BioNumerics v.7.6.3. The assembled and trimmed contigs in Fasta formats are available in the BIGSdb. The allelic profile of cgMLST scheme was analyzed by BioNumerics v.7.6.3 and the clonal relationship among isolates was investigated by MST using default parameters, and no re-sampling was performed. The allelic profiles were used as categorical data and the resulted phylogenetic network was able to illustrate the connection between isolates by displaying the mismatch between their profiles. According to Bialek-Davenet, clonal groups could be defined as related isolates which have less than 100 allelic mismatches [32]. The MST analysis was also expanded by overlaying the main characteristics of isolates such as virulence factors, ARGs, and infections sites.

The interactive tree of life, iTol V 5.5 (https://itol.embl.de/) was used to annotate the SNP-based analysis tree [33].

## Results

### Phenotypic analysis

For the bacterial isolates, after confirmation of the ESBL-KP phenotype, we finally included 193 non-duplicate clinical isolates from blood (n = 54), urinary tract infection (UTI) (n = 44) and others infection types (n = 95) (Table 1).

**Table 1:** Summary of the main findings.

All non-duplicate clinical isolates (N = 193)	
Samples (N = 193)	Blood (n = 54), UTI (n = 44), abdominal fluid (n = 5), respiratory (n = 2), drain (n = 1), vaginal discharge (n = 4), cerebrospinal fluid (n = 9), pleural fluid (n = 1), wound secretion (n = 49), and tracheal sample (n = 24).
Sampling period	January, 2011 to December, 2012.
Hospital	University Hospital Establishment of Oran, Algeria.
Phenotypic resistance to antibiotics (%)	Amoxicillin/clavulanic acid (16.6), ceftazidime (83.9), cefotaxime (99.5), cefoxitin (3.6), ertapenem (1), amikacin (20.2), nalidixic acid (42.5), ciprofloxacin (76), pefloxacin (77.5), gentamicin (91.2).
Resistance genes and enzymes (N = 193)	CTX-M-1 group (n = 191), CTX-M-9 group (n = 1), ESBL SHV 238S and 240K (n = 1), *acc(6’)-Ib-cr* (n = 140), *qnrB* (n = 123), *qnrS* (n = 4), *armA* (n = 17), CMY II (n = 4).
Combination resistance genes and enzymes (N = 193)	CTX-M-1 group only (n = 30) CTX-M-1 group, *acc(6’)-Ib-cr* (n = 28) CTX-M-1 group, *armA* (n = 6) CTX-M-1 group, CMYII (n = 1) CTX-M-1 group, *qnrB* (n = 10) CTX-M-1 group, *qnrB*, *acc(6’)-Ib-cr* (n = 100) CTX-M-1 group, *qnrB*, *acc(6’)-Ib-cr*, *armA* (n = 5) CTX-M-1 group, *qnrB*, *acc(6’)-Ib-cr*, *armA*, CMYII (n = 2) CTX-M-1 group, *qnrB*, *acc(6’)-Ib-cr*, CMYII (n = 1) CTX-M-1 group, *qnrB*, *acc(6’)-Ib-cr*, OXA-48 (n = 1) CTX-M-1 group, *qnrB*, *armA* (n = 4) CTX-M-1 group, *qnrS*, *acc(6’)-Ib-cr* (n = 3) CTX-M-9 group, *qnrS* (n = 1) SHV-Variant only (n = 1)
PFGE results	Typeable isolates (n = 191), the biggest cluster (n = 37), similarity (63.7%), 12 PFGE-clusters.
**Genome sequenced KP-ESBL (N = 19)**	
Samples	Blood (n = 7), UTIs (n = 6), and others (n = 6).
Resistance genes	
ESBL resistance genes	*bla*_*CTX-M-15*_ (n = 19), *bla*_*TEM-1B*_ (n = 12), *bla*_*SHV-variant*_ (n = 2), *bla*_*CMY-2*_ (n = 1), *bla*_*CMY-16*_ (n = 2), and *bla*_*OXA-48*_ (n = 1).
No ESBL resistance genes	*arm*A (n = 2), *qnrB1* (n = 12), *qnrS1* (n = 1), *acc(6’)-Ib-cr* (n = 11), *oqxA* (n = 19), *oqxB* (n = 19), *aph(3’’)-Ib* (n = 12), *aph(6)-Id* (n = 13), *aac(3)-IIa* (n = 12), *aadA1* (n = 3), *aadA2* (n = 3), and others
STs Total STs (17)	ST13 (n = 1), ST14 (n = 1), ST23 (n = 1), ST37 (n = 2), ST48 (n = 1), ST86 (n = 1), ST147 (n = 2), ST111 (n = 1), ST307 (n = 1), ST348 (n = 1), ST392 (n = 1), ST405 (n = 1), ST831 (n = 1), ST1426 (n = 2), ST1942 (n = 1), ST2108 (n = 1).
High risk clones	ST14 (n = 1), ST37 (n = 2), ST147 (n = 2), ST307 (n = 1).
K-Types Total K-types (n = 16)	K1 (n = 1), K2 (n = 2), K3 (n = 1), K12 (n = 1), K15 (n = 1), K18 (n = 1), K20 (n = 2), K27 (n = 1), K31 (n = 1), K61 (n = 1), K62 (n = 1), K63 (n = 1), K64 (n = 2), K102 (n = 1), K124 (n = 1), K151 (n = 1).
Virulence	Classic strains (n = 12), virulent strains (n = 7). Associated serotypes with virulence: K1 (n = 1), K2 (n = 2), K3 (n = 1), K31 (n = 1), K62 (n = 1), and K151 (n = 1).
Replicons Total isolates with replicons (n = 17)	Inc groups IncF: IncHI1 (n = 3), IncL/M (n = 2), IncL/M(poxa-48) (n = 1), IncA/C2 (n = 2) and IncR (n = 2). IncF: FII_K_ (n = 13), FIB_K_ (n = 12), FIB (pQil) (n = 3), and FIB (Mar) (n = 2). Col replicons: ColRNAI (n = 3), Col44I (n = 3), Col440II (n = 1), and ColpVC (n = 1).
CRISPR-Cas system types	Total CRISPR-Cas (n = 9), I-E (n = 5), I-E* (n = 4). Total spacers (n = 184), spacers length (22 or 23 bp), unique spacers (n = 135), matched with phages (n = 16), matched with plasmids (n = 18), matched with chromosome (n = 155), no homology (n = 26), Self-target spacers (n = 9), DRs I-E length (29 bp), DRs I-E* length (28 or 29 bp).
Phages Total phage (N = 86)	Intact (n = 51), incomplete (n = 17), questionable (n = 18). Phages carried *bla*_*CTX-M-15*_ (n = 2).

In the paper abdominal fluid, respiratory, drain, vaginal discharge, cerebrospinal fluid, pleural fluid, wound secretion, and tracheal sample were grouped as others. DRs: Direct Repeats, UTI: Urinary Tract Infection.

Antimicrobial susceptibility tests were analyzed according to CLSI standards, 99.5% of the strains were resistant to cefotaxime, 76% were resistant to ciprofloxacin, and only 3.6% were resistant to cefoxitin. Resistance to gentamicin was observed in 91.2% of isolates, while resistance to amikacin was observed in 20.2% of isolates. Two strains were resistant to ertapenem and two strains were intermediate to imipenem. Only one strain (0.5%) was intermediate to meropenem (Table 1, S2 Fig, and S3 Table). Eight isolates were tested with ROSCO test; 7 isolates had ESBL and porin loss with synergism between CAZ/clavulanate and one isolate represented no zone of inhibition to temocillin, it was featured as ESBL and OXA-48 with synergism between CAZ/clavulanate. The synergism between CAZ/clavulanate is due to the clavulanate inhibits of beta-lactamases in vitro which creates an area of synergy.

### Molecular and core genomic typing

PFGE showed that almost all of the isolates (191/193) were typeable. The PFGE analysis divided the 191 isolates into 37 PFGE types. The majority of the strains were grouped in clonal groups consisting of two or more isolates. The similarity between the isolates was 63.7% which showed a relatively high degree of relatedness between the members of the population. Importantly, the PFGE analysis has the ability to distinguish between clonally related and unrelated isolates. Cluster C11 was the most predominant (n = 35) followed by other medium and minor clusters (S1 Fig). Nineteen isolates were selected for whole-genome sequencing: seventeen representing PFGE-cluster isolates, (one isolate from each cluster, and more than one for the big clusters), and the two non-typeable isolates as well.

**Fig 1:**
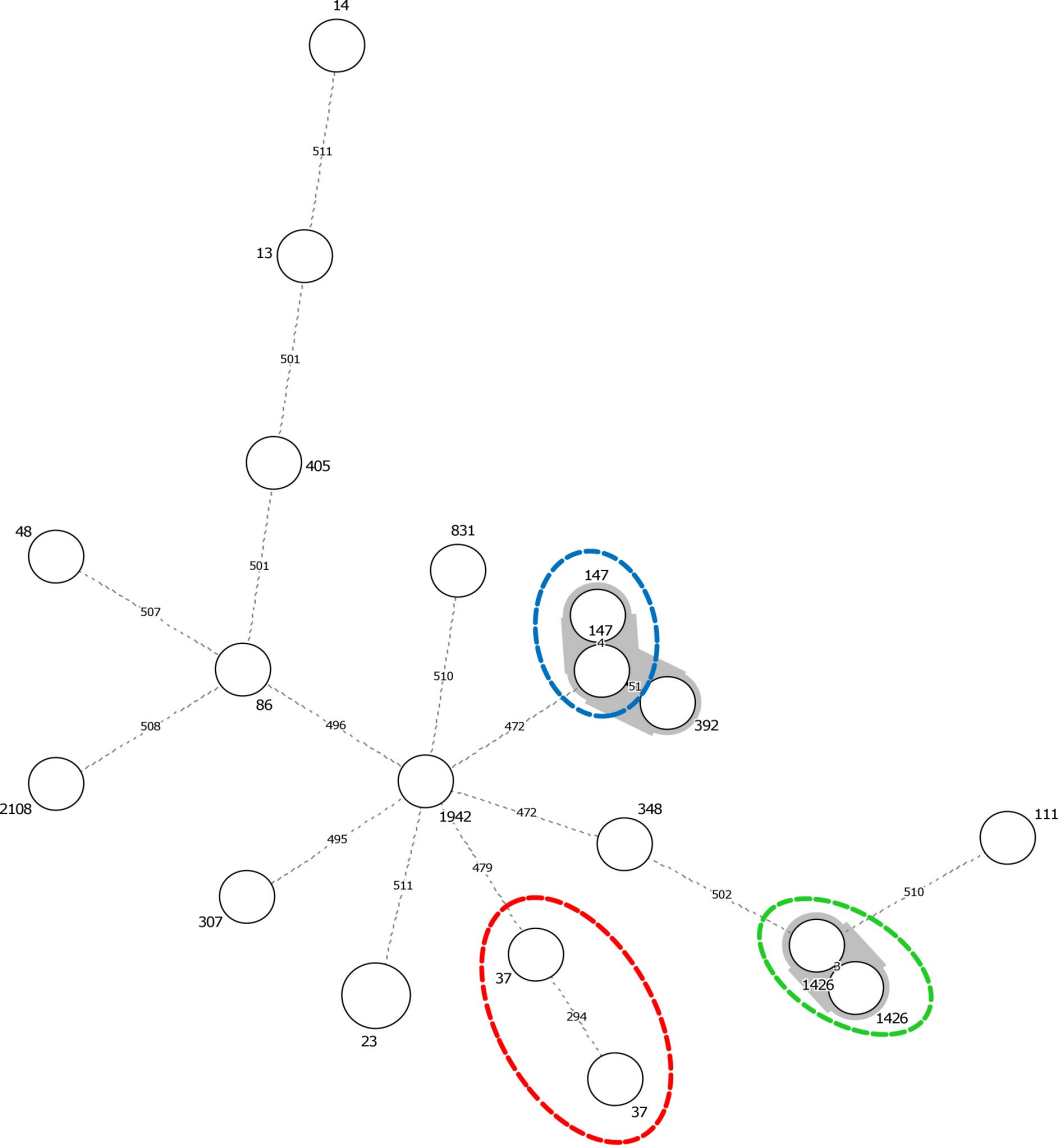
Minimum Spanning Tree (MST) analysis of 19 isolates of *K*. *pneumoniae* based on 632 allelic profiles of cgMLST. Each node within the tree represents a single isolate with a unique genotype. Selected nodes are labeled with corresponding STs. The length of branches linked two nodes indicates the number of allelic mismatches in a pairwise comparison. The oval discontinued dash in red, green, and blue demonstrate the isolates having ST37, ST1426, and ST147, respectively.

**Fig 2:**
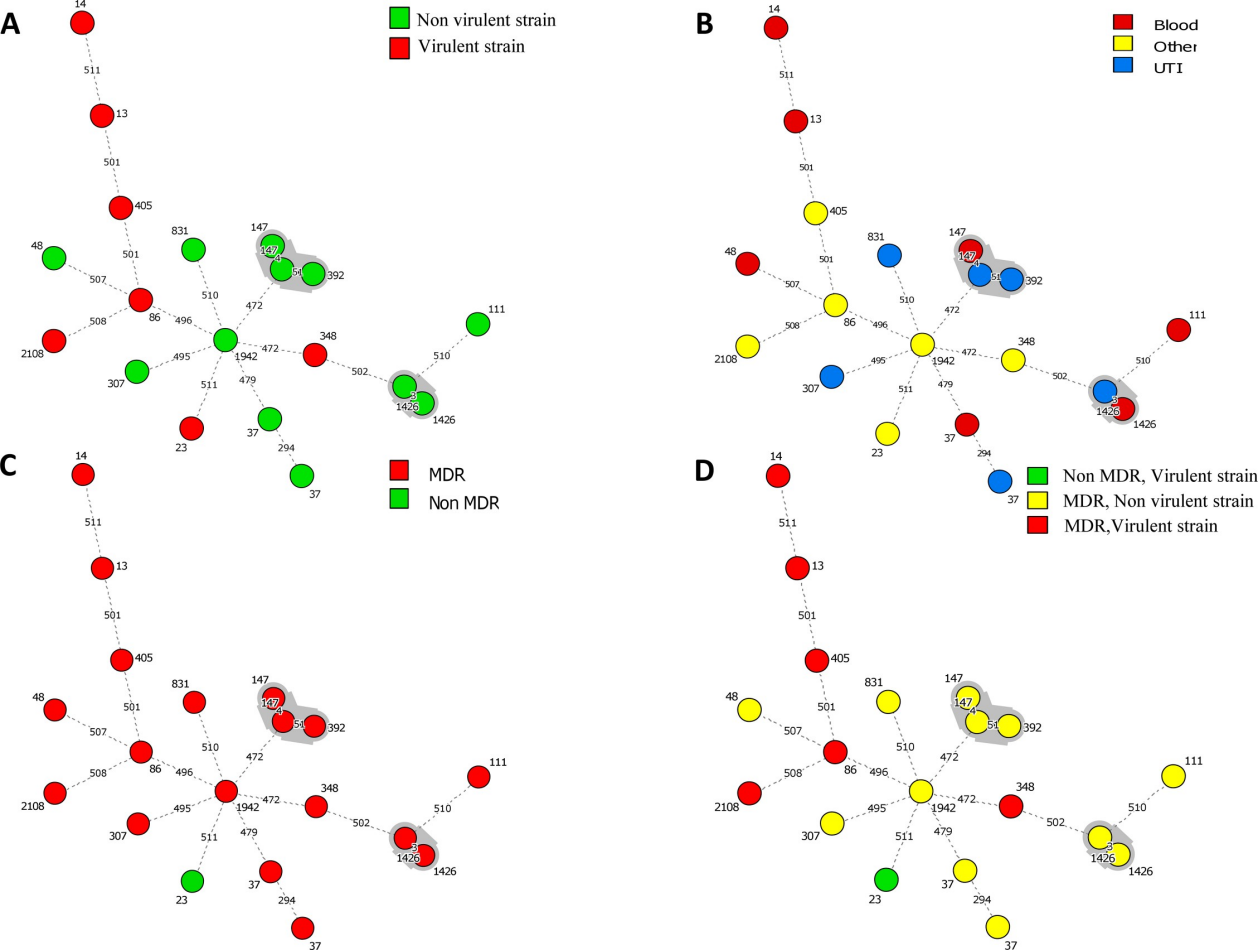
Minimum Spanning Trees (MSTs) analysis showing the distribution of MDR profiles, virulence factors, and infection types among 19 isolates of *K*. *pneumoniae* based on 632 allelic profiles of cgMLST. Each node within the tree represents a single isolate with a unique genotype. Selected nodes are labeled with corresponding STs. The length of branches linked two nodes indicates the number of allelic mismatches in a pairwise comparison. Long branches are marked with dashed lines. Thick lines correspond to a short branch with less than 100 allelic mismatches. The grey zones surrounding nodes indicate that they belong to the same clonal group. Four MST graphs were generated separately based on the following associations. A: MST versus virulence genes, B: MST versus infection types, C: MST versus resistance genes profiles, D: MST versus MDR and virulence classes. MDR: Multi-Drug Resistance. UTI: Urinary Tract Infection.

cgMLST analysis revealed 16 distinct STs and most of them were well described in the literature. Each PFGE cluster was assigned a distinct ST. The largest cluster C11 had two related STs—ST392 and its single locus variant (SLV) ST147. Two distinct clusters (C9 and C10) were shown to belong to the same sequence type, ST37. The MST disclosed the relatedness of isolates and the connection among STs. We downloaded profiles of all isolates and their STs from the BIGSdb-Kp to draw the MST based on their cgMLST profiles seen in Figs 1 and 2, demonstrate three characteristics: first, the isolates were divergent, having a high number of allelic mismatches (472 to 511). Three isolates of Cluster C11 were found to be linked together in the same clonal group. Second, the cgMLST632 scheme was able to distinguish between strains that had the same ST, for instance in ST147 and ST1426. The number of allelic mismatches within identical STs was very low (i.e.3 and 4 allelic changes within ST1426 and ST147 isolates respectively). Third, the cgMLST increased the resolution in two isolates of ST37 (differed by 294 loci) and showed a high complexity within these isolates suggesting that ST37 isolates are heterogeneous.

**Fig 3:**
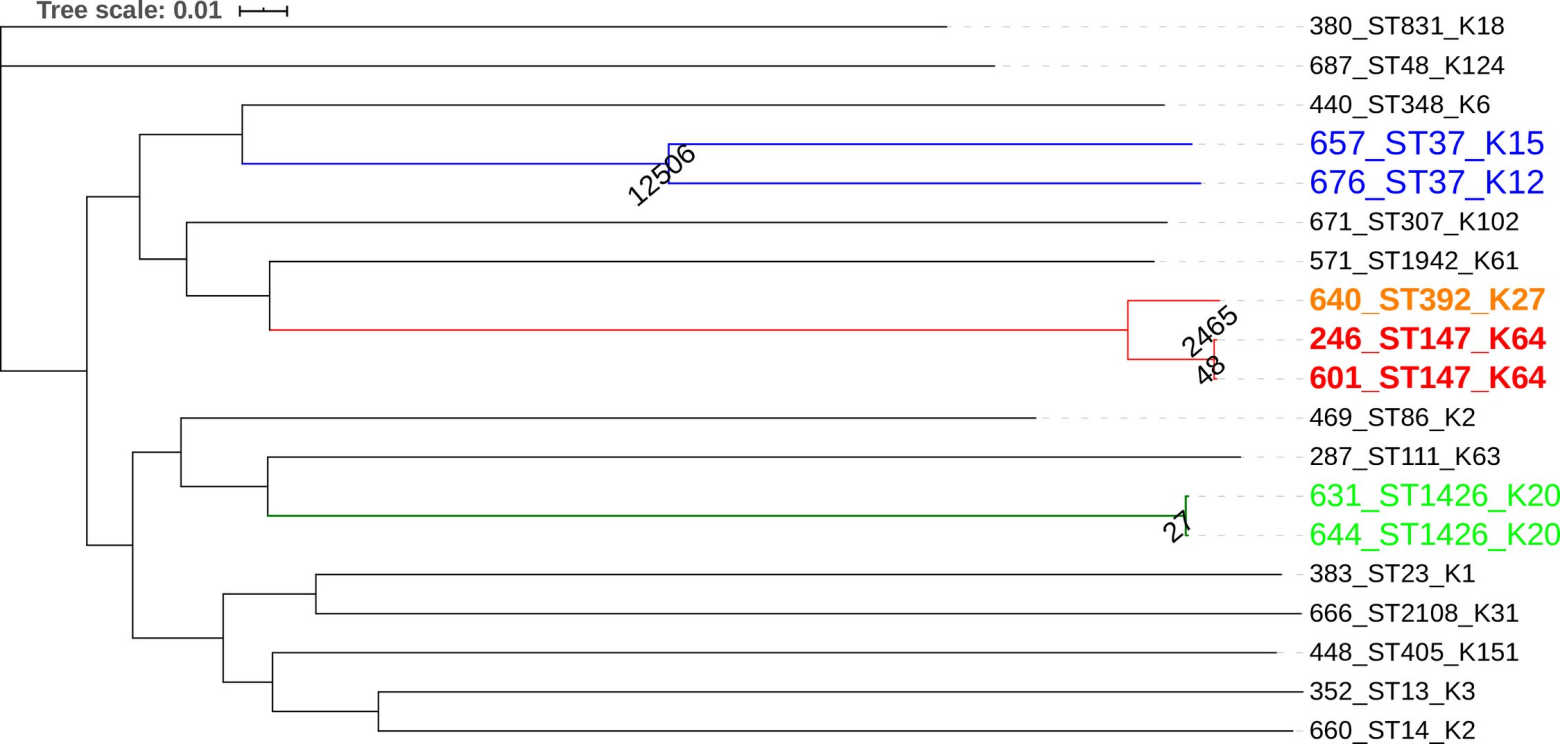
Maximum likelihood tree created from concatenated SNPs sequences. The numbers on the nodes represent the SNPs differences between isolates.

SNP-based phylogenetic analysis revealed a sharp discontinuity within isolates. Identical to the cgMLST results, the Maximum Likelihood tree (MLTree) displayed two minor groups of related isolates, but a majority of unrelated isolates were apparently clustered in heterogeneous branches that were well separated from each other. The SNP-based phylogenetic approach identified closely related isolates which were previously obtained by PFGE and cgMLST but with much finer resolution. Furthermore, the phylogenetic analysis revealed congruence between SNP-based analysis and cgMLST, especially within closely related isolates. In total, 85,965 SNPs were present in the alignment, with 19,701 the average SNPs differences over all sequence pairs. Interestingly, SNP-based analysis provided optimal resolution especially within identical STs; ST1426 isolates differed with 27 SNPs (Fig 3).

### Genotypic and ARGs analysis

Screening of ARGs by PCRs showed that CTX-M-1 group was the predominant with 98.96% (191/193), only one strain was from the CTX-M-9 group and one strain had SHV-type (238S+240K). About the PMQR, the prevalence of *aac(6’)-Ib-cr* and *qnr* genes was very high 72.5% and 65.8%, respectively. Among *qnr* gene, 63.7% were *qnrB*, whereas 2.1% were *qn*r*S*. Among amikacin-resistant strains (24/193), we found that 17 isolates were positive for *armA*, and 7 isolates were negative for all 16S rRNA methylase encoding genes. Among the isolates resistant to cefoxitin 6/193, 4 had the CMY II and 2 isolates were negative for all pAmpC enzymes. The most prevalent combination was CTX-M group 1, *qnrB* and *aac(6’)-Ib-cr* with 51.81% (100/193) (Table 1).

**Fig 4:**
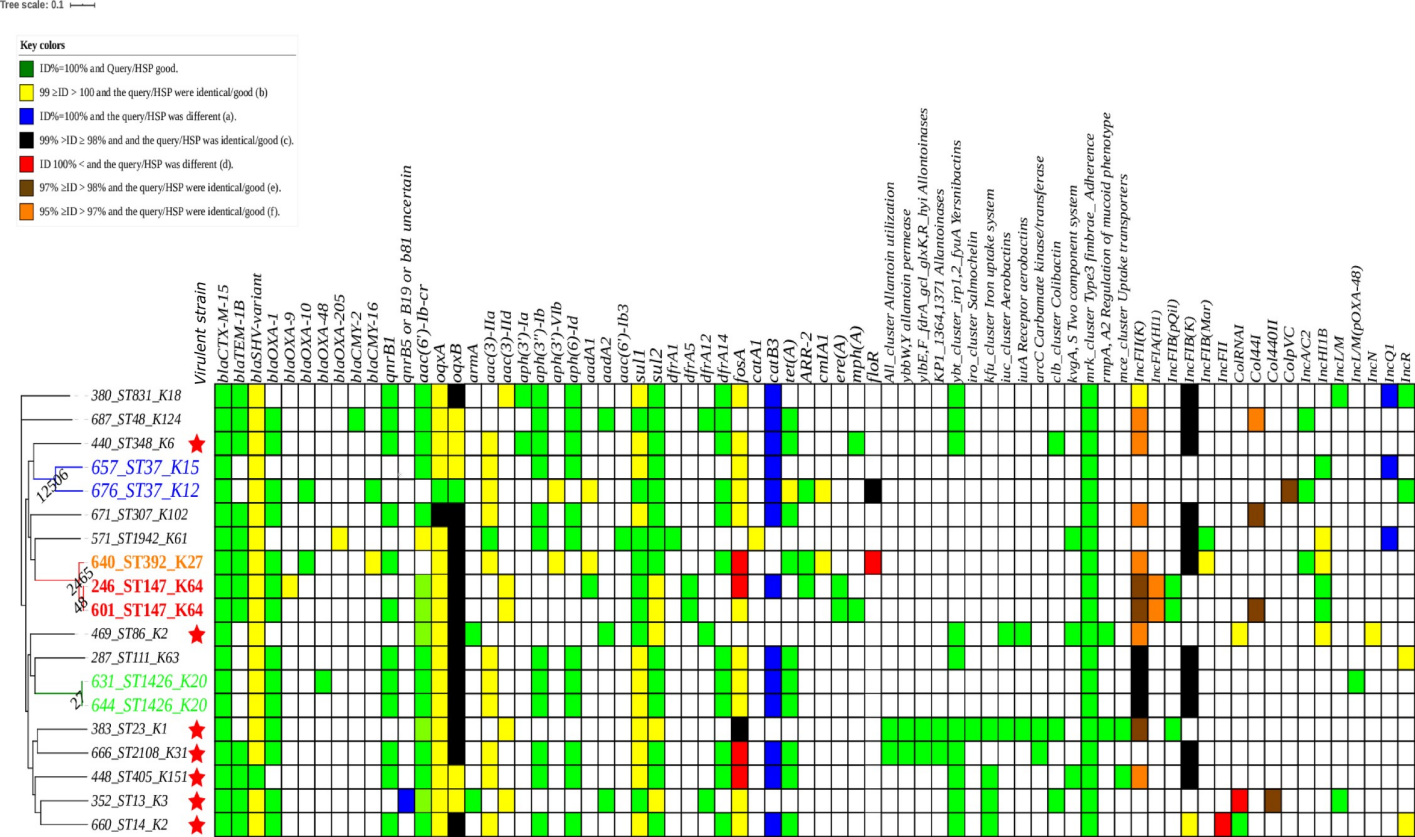
Heatmap with all results (resistance genes, virulence, MLST, K-types, replicons with the ML Tree from the SNP-based analysis) among the sequenced 19 isolates. The scale is modified.

Mapping ARGs revealed that β-lactamases genes were found in all isolates: *bla*_*CTX-M-15*_ was found in all isolates, *bla*_*TEM-1B*_ (12/19) whereas *bla*_*SHV-variant*_ (2/19). Regarding carbapenemase genes, only one strain had *bla*_*OXA-48*._ Two variants of pAmpC were found *bla*_*CMY-2*_ (1/19) and *bla*_*CMY-16*_ (2/19). The aminoglycosides resistance genes found were: *armA* (2/19), *aac(3)-IIa* (1/19), *aadA2* (3/19), *aadA1* (1/19), *aph(3’’)-Ib* (12/19), and *aph(6)-Id* (12/19). Resistance genes to fluoroquinolones were detected frequently; both *qnrB1* (12/19) and *aac(6’)-lb-cr* (11/19), the latter confers simultaneous resistance to aminoglycosides and fluoroquinolones. We rarely found *qnrS1* (1/19). Almost all isolates had the concomitant presence of resistance determinants to β-lactams, aminoglycosides, and fluoroquinolones. We observed the presence of phenicol, sulfonamide, tetracycline, and macrolide resistance genes (Table 1 and Fig 4).

### Capsular serotype and virulence genes

Typing of the capsular *cps* (*wzc* and *wzi*) revealed sixteen K-type: 1, 2 (n = 2), 3, 6, 12, 15, 18, 20 (n = 2), 27, 31, 61, 62, 63, 64 (n = 2), 102, 124, and 151. K2 was found in two isolates having different STs (ST14 and ST86). The isolates having the same STs, ST147, and ST1426 had the same K-type K64 and K20 respectively (Table 1).

All sequenced isolates had at least *mrk* cluster genes encoding to fimbriae type III, thus giving biofilm formation phenotype. The isolate having the ST23 and K1 was the most virulent carrying yersiniabactin, aerobactin/receptor, allantoinases, and fimbriae type III clusters, in addition to *iro*, *kfu*, *clb*, *mce* clusters, and *rmpA/A2*. The following K-types: K1 (ST23), K2 (ST14 and ST86), K3 (ST13), K31 (ST2108), K6 (ST348), and K151 (ST405) were found in virulent strains (Table 1 and Fig 4).

### Analysis of mobile genetic elements

In total 17 plasmid replicon types were detected (Table 1). The identified replicons were from plasmid incompatibility groups (IncF, IncHI1, IncL/M, IncA/C2, and IncR), and Col plasmids replicons (ColRNAI, Col44I, Col440II, and ColpVC). The replicons were FII_K_ (n = 13), FIB_K_ (n = 12), FIB (pQil) (n = 3), and FIB (Mar) (n = 2). The last replicon was found in two isolates and it was simultaneously present with the FIBK. The replicon sequence typing (RST) scheme discriminating of IncF plasmid variants revealed that the same FAB Formula K5:A22: B- in the isolates having the same ST147. The ST of the replicon IncA/C was ST3 or it was the nearest. For the replicon IncN was ST9.

The analysis of the prophages showed that all strains had at least one prophage. The highest number was observed in the isolate having ST111 with 9 prophages. Crossed analysis between the position of the resistance genes and the prophages revealed that *bla*_*CTX-M-15*_ was hosted in the incomplete sequence of the phage SJ46 in two isolates (ST86 and ST1942), (Table 1).

### Analysis of CRISPR-Cas system

**Fig 5:**
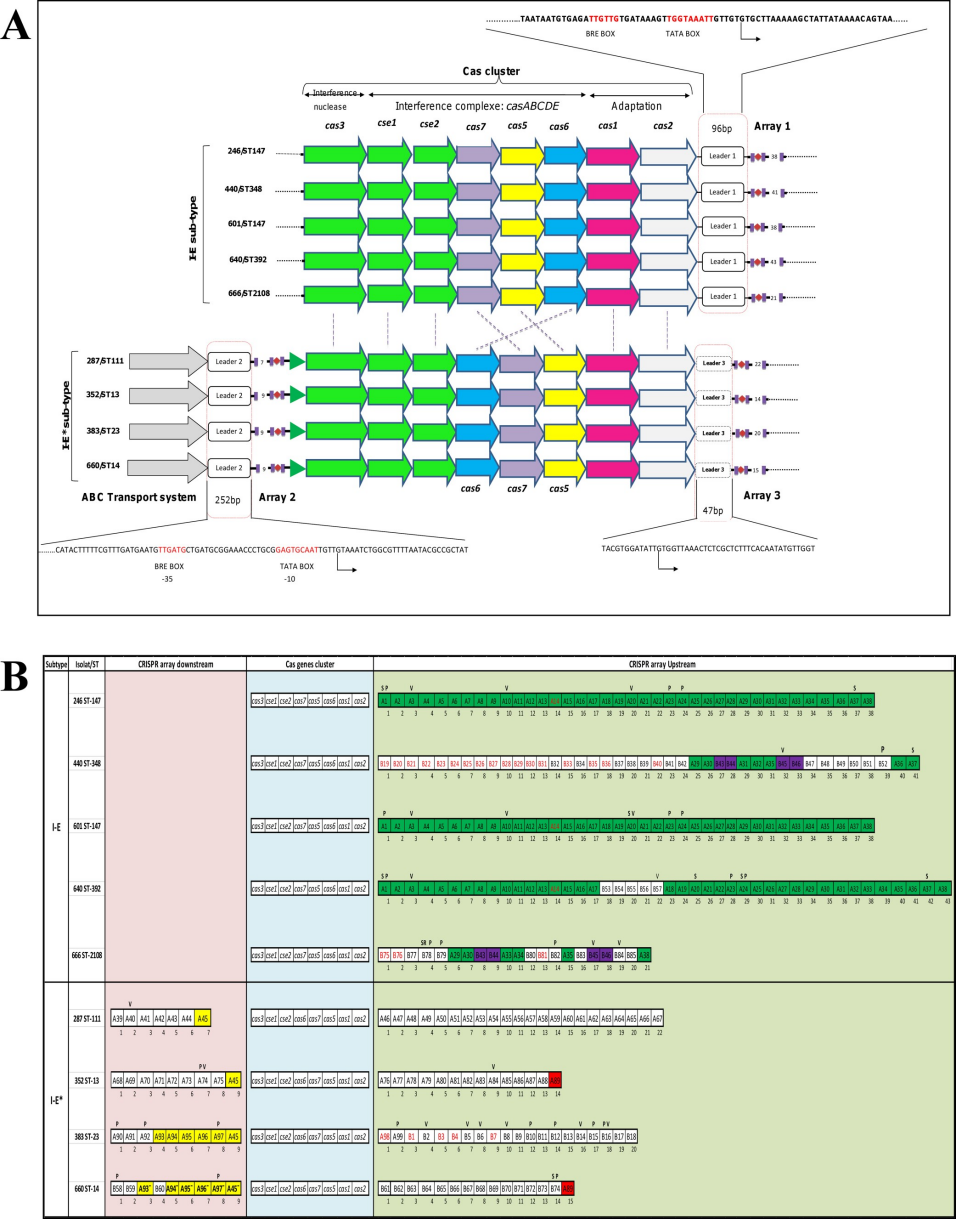
Architecture and organization of the CRISPR-Cas system in clinical ESBL-KP isolates. A. The operon organization (*cas* genes), the leaders, classification, and core-promoter. The rows represent *cas* genes in different colors, the brown diamonds represent the spacer, and the blue rectangle represents the Direct Repeated (DR). B. Spacer map, their distribution, and the matching results. Spacers are represented in a box without repeats. Identical spacers are represented by the same ID and color. P: Plasmid, V: Virus or Phage, S: Self-target, RS: The reverse self-target, spacers in red had no homology. +: Longer spacer.

In this study, the occurrence of the CRISPR-Cas system was 47.36% (9/19 isolates). in Fig 5A, two groups are seen; the first, consists of 5 isolates sub-typed I-E and the second is composed of 4 isolates sub-typed I-E*. In our results, the isolates having STs: 392, 147, 2108, 348 were typed I-E, and those who had STs: 13, 23, 111, and 14 were typed I-E*. The anti-CRISPR was absent in all isolates having the CRISPR-Cas system. Isolates having the sub-type I-E had the highest number of spacers.

A total of 184 non-duplicated spacers were found; 135 spacers were unique and 49 were shared between CRISPR arrays. Blasting spacer’s results showed 158 spacers had perfect homology spacers targeting with 16 bacteriophages, 18 plasmids, and 155 chromosomes. Some spacers matched with chromosomes, plasmids, and/or phages. Isolates within the same ST (ST147) had the same array and organization. The rate of self-targeting spacers was 5% (9 of 184) found in 5 of 9 isolates (55.55%) (Table 1 and Fig 5B).

Three leader groups were found: leaders 1 (96bp), leaders 2 (252bp), and leaders 3 (47bp). The first for I-E subtype and the two latest for the I-E* sub-type. Leaders1 and leaders 3 were conserved but leaders 2 had 96.4% of identity. Concerning the core promoter (-10, -35 and promoter) was found for leaders 1 and leaders 2, while leaders 3 had only the promoter. Searching upstream the leaders 2 an ABC transport system was found (Fig 5A).

## Discussion

Herein, the data presents genomic epidemiology and surveillance study in ESBL-KP clinical isolates between 2011 and 2012, from one hospital “EHUO” in Oran, Algeria. Phylogenetically, the isolates were genomically diverse and characterized by multiple combinations of ARGs. ESBLs are usually associated with multidrug resistance and have become a great challenge for infection control in human health that resulted in treatment failure [34,35]. Herein, the CTX-M group 1 was found in almost all isolates (99%) and featured later *bla*_CTX-M-15_ in the 19 sequenced isolates, which was expected due to its predominance in most parts of the world [36–40]. Rodrigues *et al*. reported that CTX-M-15 was associated with the increase of MDR-KP epidemic clones (ST15, ST147, and ST336) [41]. In our study, two isolates were ST147 and could represent a higher risk in the nosocomial setting, due to the propensity of causing outbreaks. The predominant replicons were IncFII_K_ and IncFIB_K_. It was reported that *bla*_CTX-M-15_ is mainly harbored on IncFII_K_ plasmids in ESBL-KP isolates and it was described as dynamic, with the capacity to disseminate ARGs among Enterobacteriaceae [34,42–44]. The characteristic combination *bla*_CTX-M-15_, *bla*_TEM-1B_, *bla*_OXA-1_, *qnrB*, and *aac(6′)-Ib-cr* was found in 8/19 isolates and 7/8 were also associated to IncF replicon group which was previously partially described in Morocco; *bla*_CTX-M-15_, *bla*_TEM-1b_, *bla*_OXA-1_, *aac(6′)-Ib-cr*, and *qnrB1* genes were co-transferred and carried by a conjugative plasmid of high molecular weight [45]. In Algeria, the first report of OXA-48-producing KP located in IncL/M-type plasmid, belonging to the pandemic ST101 clone, isolated in May 2014 [46]. Our data show, OXA-48-producing KP was isolated from a patient with UTI in 2012 and belongs to the ST1426 non-epidemic clone, IncL/M replicon, and *bla*_*OXA-48*_. This means that in Algeria, *bla*_OXA-48_ was present earlier than the first published report.

Regarding AmpC enzymes, CMY-2 type was found, featured as *bla*_CMY-2_ and *bla*_CMY-16_. CMY-2 is the most widespread type of AmpC enzyme with the widest geographical distribution, including Algeria [47,48]. During the last two decades, PMQR has been reported by the acquisition of *qnr*, *qepA*, and *aac(6’)-Ib-cr* genes [49]. It was previously described that *qnrB* was predominant among strains from Africa while *qnrS* was mainly detected in strains from Vietnam [50]. Our results showed a high prevalence of PMQR genes *aac(6’)Ib-cr* (72.5%), *qnrB* (63.7%) and rarely *qnr*S (2%), featured by WGS as *qnrB1* and *qnrS1*. The first report of *qnrS1*, *qnrB1* and *qnrB4* was from *Enterobacter cloacae* clinical isolates from Algerian hospitals, in 2008 [51]. In Algiers, *Klebsiella oxytoca* was isolated from wastewater hospital had PMQR genes (*qnrB1* and *aac(6’)Ib-cr*) [37]. This study is the first report of *qnrS1* in ESBL-KP in Algeria. As previously described, clinical isolates carrying *armA* have been associated with IncL/M plasmids, with the concomitant presence of the *bla*_*CTX-M3*_ [52]. Also, *armA* was identified on IncN plasmids, in animals [53]. In the present study, *arm*A was found in two isolates; one had IncL/M replicon and the second IncN replicon as ST9. This suggests that animal contact and food (animal meat) represent a potential reservoir and a route for dissemination of IncN plasmid harboring *arm*A to humans by HGT.

Herein, an incomplete sequence of *Salmonella* phage was found hosting *bla*_CTX-M-15_. It was previously described that bacteriophages could contribute to the spread of ARGs among Enterobacteriaceae isolated from animals and food, highlighting the role of HGT of ARGs and *bla*_*CTX-M*_ particularly [54,55]. Thus phages could be a reservoir to spread ARGs by transduction.

About the virulent strains, several serotypes of K antigens have been reported around the world, the most studied were K1 and K2 because they were associated with a serious infection [56,57]. It was found that the K1 was associated with the ST23 and K2 was found in several STs: 25, 65, 86, 380, 14, 66, 374, 493, and 1013 (SLV ST5) [58]. In this study, the virulent strains were K1 (ST23), K2 (ST14 and ST86), K3 (ST13), K6 (ST348), K31 (ST2108), and K151 (ST405).

The MST analysis about the ARGs, virulence factors, and infection types provided an overview of the sequenced isolates, their combination, and relatedness. Fig 2 showed that three isolates of CG147 were isolated from different origins, with few virulence factors (classic) and MDR. Regarding the CG1426, the related isolates were also isolated from distinct origins, possessed few virulence factors and not presenting the same ARGs profiles. The combination MDR and virulent strains was seen in 6 of 19 strains (2 cases of bloodstream infection and 4 other cases). The combination of MDR and virulence genes presents a high health risk by increasing mortality and antibiotics less efficient. It was reported that the prevalence of KP having the combination MDR and hyper-virulence is increasing and our data would provide further arguments for enhancing the clinical awareness for *K*. *pneumoniae* having the combination MDR and virulence [57].

In three studies, the CRISPR-Cas system in KP genomes had a variable occurrence ranging between 11.5% and 54.4% [14,59,60]. A relation could be seen between STs, CRISPR-Cas type, and types of samples; regarding UTI, from 6 isolates: 4 had no CRISPR-Cas system and 2 had CRISPR-Cas system type I-E. While blood samples, from 7 isolates: 3 isolates had no CRISPR-Cas system, 3 isolates had I-E* and one isolate had I-E. In another study, the subtype I-E was found in isolates mostly from UTI samples and having ST34, 45, 66, 67, 147, 273, 383, 392, and 941, while the subtype I-E* was found in isolates from blood samples present in ST14, 15, 23, 111, 374, 493, and 505 [60]. The leader sequence length is variable, but must be at least 60 bp in length and can be up to 500 bp. Apparently, the sequence length is relatively conserved within a species [12,61]. The leaders’ lengths corroborate with previous work from Shen *et al* [14]. Paradoxically, the leaders 3 lacked (-10) and (-35) sites required for the array transcription. Yosef *et al*. suggested that (-10) and (-35) sites are essential for the interference and not for the adaptation process [62]. Thus, self-target spacers in the loci downstream the leaders 3 could not have auto-immunity function.

The PFGE method was able to distinguish between strains and accurately define dominant clones. Currently, WGS-based typing approaches have become international typing reference methodology. Accordingly, the seven-gene MLST pattern can easily be extracted from WGS data; the analyzed genomes could be sorted into 16 different STs. MLST analysis has shown that the spread of ESBL-KP is largely multi-clonal, by contrast, the international spread of KPC-KP is limited to specific clones. The PFGE and MLST identified that the largest cluster (C11) was ST147 (n = 35), this sequence type poses a serious public health threat worldwide.

In our study, the analysis of five major groups C1, C7, C8, C9, and C11 showed twenty-eight resistance patterns. The relationship between resistance patterns and pulsotype demonstrated that the strains from C11 had mostly the pattern CEP CTX CAZ NAL PEF CIP GEN (42.8%) but this same resistance profile belonged to the PFGE-clusters (S4 Table). So far, there is no significant association between the resistance patterns and pulsotypes of ESBL-KP isolates, the same statement was previously described in several studies concerning KP clinical isolates [63,64]. Chromosomally identical *K*. *pneumoniae* strains had different resistance profiles; this is due to the presence of plasmid-mediated resistance genes [64,65]. Phenotypic methods such as antimicrobial susceptibility tests had a weak discriminatory power [65].

The first cgMLST scheme employed was based on a total of 694 highly conserved genes [47], and cluster analysis based on the data-enabled precise definition of globally distributed virulent strains and MDR clonal groups. During this study, we identified the CGs and used the same rule of Bialek-Davenet *et al* for the definition of CGs for which KP cgMLST CGs were defined as groups of cgMLST profiles ≤ 100 allelic mismatches. The cgMLST gives an overview of major CGs and yielded a satisfactory resolution within identical sequence types but it is not sufficient for outbreak investigation as set out in our study. Hence we join the conclusion that the authors highlighted the need to combine additional loci to gain more resolution required to understand the recent transmission events for epidemiological purposes.

More recently, Zhou *et al* have designed an extended scheme of cgMLST based on a total of 1,143 conserved genes from 671 KP genomes [66]. The novel extended cgMLST scheme was able to distinguish outbreak from non-outbreak isolates and highlighted the possibility to reveal several sub-clones of epidemic ST11 clone. Furthermore, based on this cgMLST, a threshold with <10 alleles difference was applied for clustering outbreak strains. In the same context, few studies have validated this strict cut-off (< 10 allele difference) for tracing local and regional clusters of KPC ST512 from several healthcare facilities in Finland, and to seek for an eventual epidemiological link between MDR-KP ST348 isolates in horses and humans in Portugal [67,68].

In addition to cgMLST, we conducted an SNP-based analysis and constructed a deep phylogeny that provided reliable and concordant results. Firstly, to differentiate between isolates (631 and 644 isolates) within identical STs; ST1426 isolates differed with 27 SNPs and secondly, to know the occurrence of these isolates was sporadic despite their close date of isolation (1 month). The isolates have shown a slight difference in their profiles of ARGs and replicon types (Fig 2). The *bla*_OXA-48_ and its associative IncL/M replicon type were restricted to the 631 isolate. The same characteristics for the two isolates (601 and 246 both had ST147) with the exception that differed with 48 SNPs and isolated separately in an interval of time of one year. These isolates are distantly related to their SLV ST392 (2,465 SNPs). In the same context of identical STs (ST37), the amplitude of polymorphism reached 12,506 SNPs, due to a major recombinational event. The SNP-based analysis provided insight into the genetic diversity of the detected clones of ESBL-KP and has proven its capability for readily scaling the SNP differences among unrelated and closely related isolates. However, it is still unclear whether a simple or strict SNP threshold is appropriate for case definition of an outbreak. The SNP thresholds vary widely even for one pathogen and the difficulty to define allele/SNP threshold remains a matter of discussion in outbreak simulations [69]. Nowadays, the majority of analyses are based on SNP-based approaches which provided an exceptionally high resolution. However, these approaches are on the other hand more flexible as they are not subjected to predefined schemes. The analyses are dependent on the quality of sequencing, assembly, and essentially the selection of reference genome. Consequently, such approaches tend to be less standardized and can hamper the comparability between different studies, especially if different parameters are used i.e. different reference genome. Alternatively, for some species including *K*. *pneumoniae* the BacWGSTdb is a useful, free, and public database for typing and tracking outbreak sources [70].

There is still a paucity of surveillance and epidemiology data of antimicrobial resistance in Africa generally and Algeria specially. The reports from the Algerian surveillance system are statistically not representative of the situation; few labs are collaborating comparing to the size of the country. To complement current approaches, sentinel genomic surveillance of MDR-virulent-KP could be of high public health importance in Algeria.

## Conclusions

This study revealed the dissemination of ESBL-producing *K*. *pneumoniae* in the EHUO, and showed that the hospital is a reservoir of highly pathogenic lineages. Importantly, WGS provided a deeper insight into the epidemiological characterization of major clones, including resistome, virulome, plasmid replicon typing, phage typing, and CRISPR-Cas contents. The cgMLST and SNP-based analysis were able to display the phylogenetic relationship among the studied clones; our finding pointed out a polyclonal spread of ESBL-KP with high genetic diversity. Furthermore, the combination of the PFGE and SNP-based analyses provided optimal resolution for the clinical isolates, indicating no evidence of a classical outbreak between 2011 and 2012 in this hospital. In terms of resistome and virulome, the strains demonstrated an MDR and/or virulence profiles. These high-risk strains pose a serious challenge for clinical prevention, diagnosis, and treatment. Undoubtedly, there is a compelling need for more comprehensive approaches to understand the evolution and transmission of these successful high-risk clones. To our knowledge, this is the first report of *qnrS1*, virulence genes, and CRISPR-Cas system in *K*. *pneumoniae* in Algeria. Finally, ESBL-KP isolates cause nosocomial infections and therefore public health problems, more genomic surveillance should be a priority in the hospital environment.

## Supporting information

S1 Fig Pulsed-field gel electrophoresis after *XbaI* digestion, Genomic DNA macro-restriction profiles of 193 clinical isolates ESBL-KP. (PDF)

S2 Fig Graph of the interpretive results of antimicrobial susceptibility according to CLSI 2020. (DOCX)

S1 Table Primers sequences. B: G or T or C, M: A or C, V: G or A or C, H: A or T or C, S: G or C, Y: T or C.
(DOCX)

S2 Table Sequencing quality of the nineteen sequenced isolates. (XLSX)

S3 Table Interpretive results of antimicrobial susceptibility according to CLSI 2020, 193 clinical isolates ESBL-KP. A: Salmonella breakpoints.
(DOC)

S4 Table Relationship between resistance patterns and PFGE-pulsotype. AMC: Amoxicillin/clavulanic acid, CAZ: Ceftazidime, CTX: Cefotaxime, CEF: Cefepime, FOX: Cefoxitin, ERT: Ertapenem, IMP: Imipenem, MEM: Meropenem, AMK: Amikacin, GEN: Gentamicin, NAL: Nalidixic acid, CIP: Ciprofloxacin, PEF: Pefloxacin.
(DOCX)

S1 Raw images (PDF)
