# Supplementary material for: High-risk clones of extended-spectrum β-lactamase-producing Klebsiella pneumoniae isolated from the University Hospital Establishment of Oran, Algeria (2011–2012)
Source: PLoS One. 2021 Jul 26;16(7):e0254805. doi: 10.1371/journal.pone.0254805 (PMC8312963; doi:10.1371/journal.pone.0254805)
Supplement: S1 Fig — (PDF) [file pone.0254805.s001.pdf]

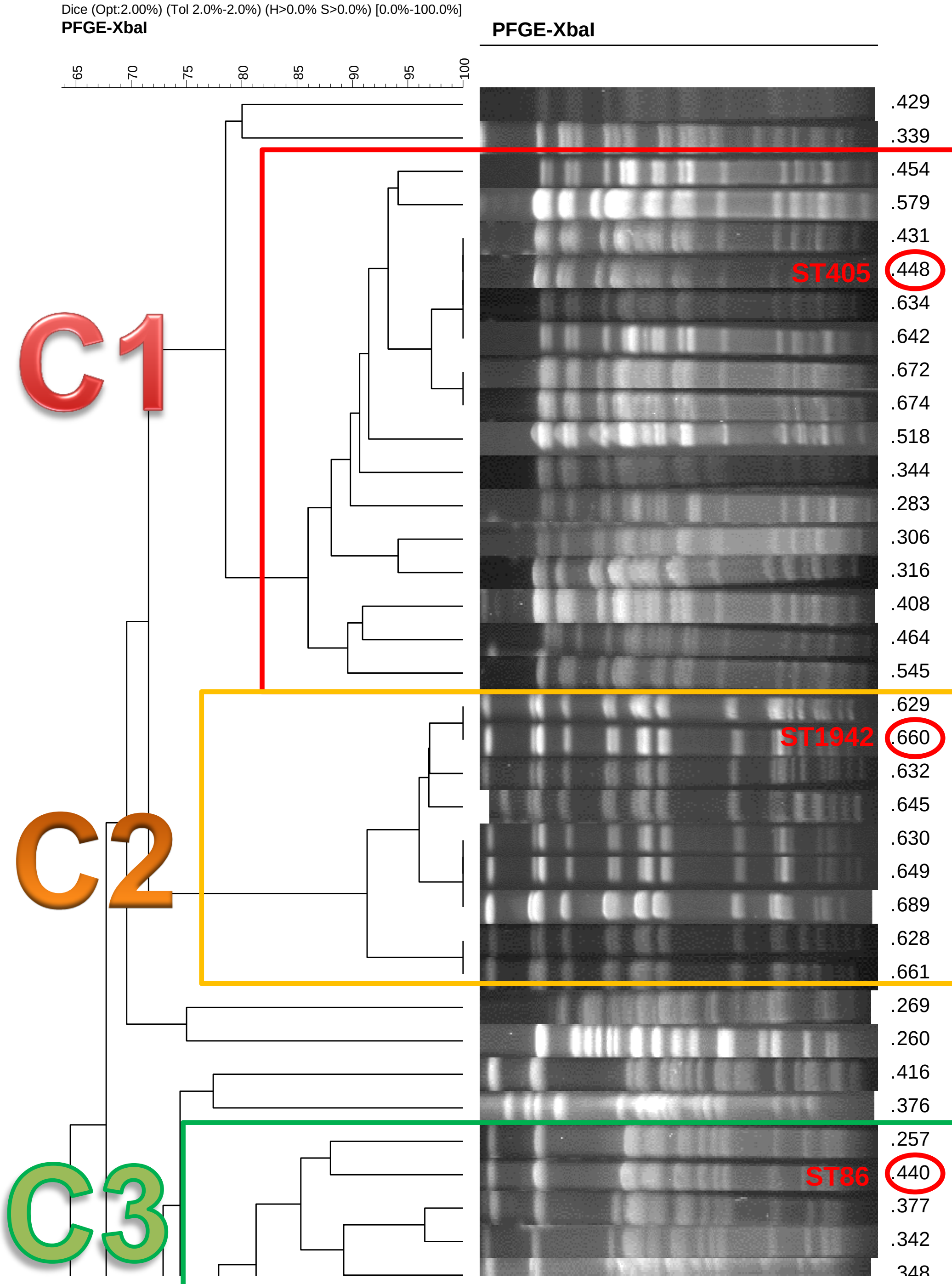

.429	ab	14-09-2011	Surgical medical emergency
.339	sa	29-05-2011	Surgical neurology
.454	tr	25-10-2011	Medical reanimation
.579	cc	11-03-2012	Orthopedic surgery
.431	lc	24-08-2011	Neonatal
.448	tr	09-10-2011	Medical reanimation
.634	ab	31-07-2012	General surgery
.642	cc	08-08-2012	Surgical reanimation
.672	ps	02-10-2012	Surgical cardiac
.674	dn	04-10-2012	Surgical cardiac
.518	gf	04-01-2012	Genycology
.344	ps	07-06-2012	Orthopedic surgery
.283	sa	21-03-2011	Reanimation medico-surgical emergency
.306	cc	30-03-2011	Surgical reanimation
.316	sa	28-04-2011	Hematology
.408	tr	02-09-2011	Surgical reanimation
.464	ps	04-11-2012	Orthopedic surgery
.545	ps	12-02-2012	Reeducation
.629	sa	16-07-2012	Reanimation medico-surgical emergency
.660	cc	15-09-2012	Reanimation medico-surgical emergency
.632	sa	22-07-2012	Reanimation medico-surgical emergency
.645	sa	13-08-2012	Surgical reanimation
.630	tr	17-07-2012	Reanimation medico-surgical emergency
.649	tr	14-08-2012	Reanimation medico-surgical emergency
.689	tr	08-11-2012	Reanimation medico-surgical emergency
.628	tr	16-07-2012	Reanimation medico-surgical emergency
.661	lc	12-09-2012	Surgical reanimation
.269	cc	08-03-2011	Medical reanimation
.260	cc	01-03-2011	Medical reanimation
.416	ps	07-09-2011	Orthopedic surgery
.376	ur	24-07-2011	Nephrology
.257	ps	23-02-2011	Gastrology
.440	ps	28-09-2011	Nephrology
.377	ps	26-07-2011	Surgical vascular
.342	ps	04-06-2011	Surgical vascular
.348	sa	10-06-2011	Medical reanimation

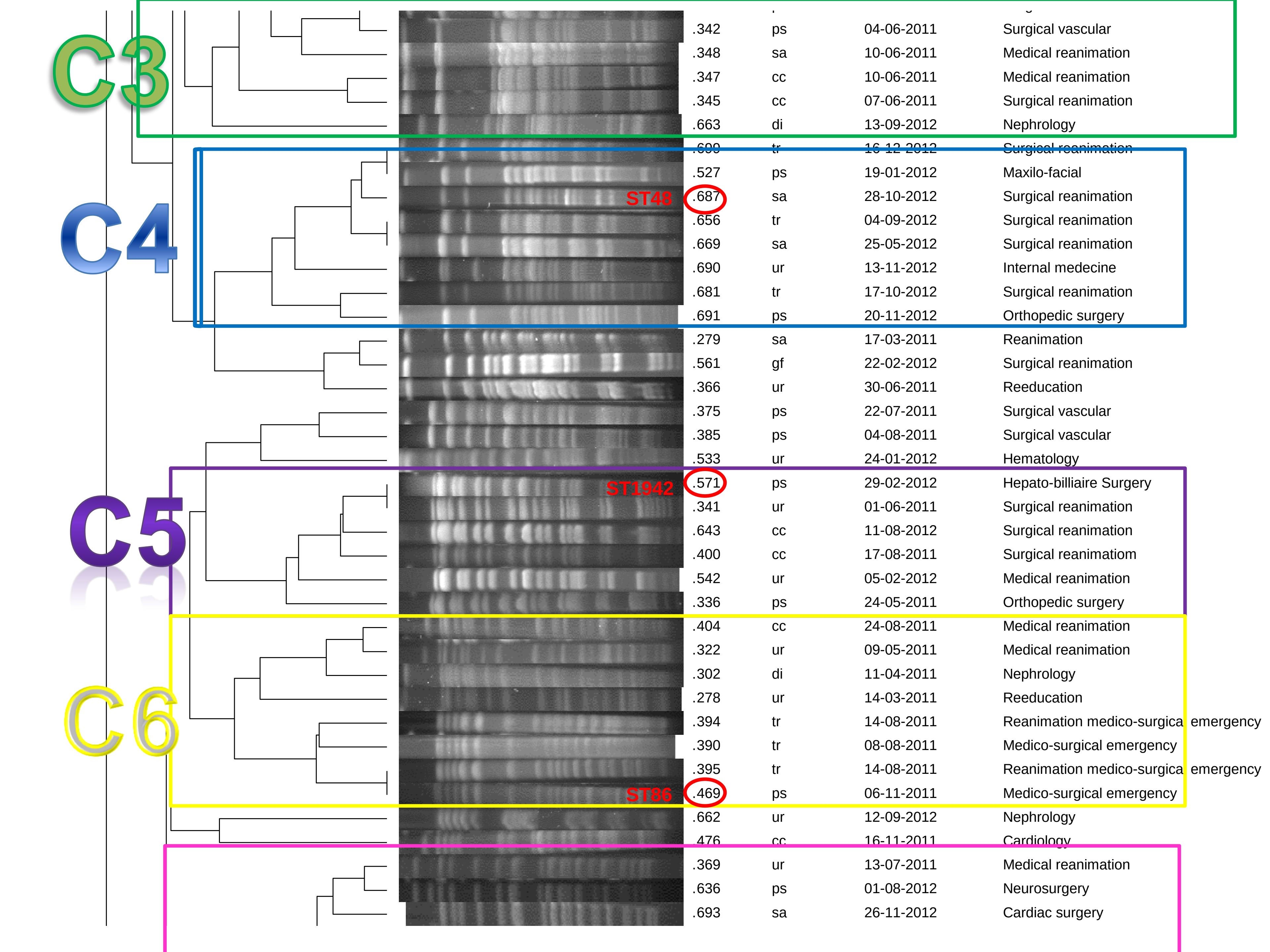
C3
C4
C5
C6
ST48
ST1942
ST86
.342 ps 04-06-2011 Surgical vascular
.348 sa 10-06-2011 Medical reanimation
.347 cc 10-06-2011 Medical reanimation
.345 cc 07-06-2011 Surgical reanimation
.663 di 13-09-2012 Nephrology
.699 tr 16-12-2012 Surgical reanimation
.527 ps 19-01-2012 Maxilo-facial
.687 sa 28-10-2012 Surgical reanimation
.656 tr 04-09-2012 Surgical reanimation
.669 sa 25-05-2012 Surgical reanimation
.690 ur 13-11-2012 Internal medecine
.681 tr 17-10-2012 Surgical reanimation
.691 ps 20-11-2012 Orthopedic surgery
.279 sa 17-03-2011 Reanimation
.561 gf 22-02-2012 Surgical reanimation
.366 ur 30-06-2011 Reeducation
.375 ps 22-07-2011 Surgical vascular
.385 ps 04-08-2011 Surgical vascular
.533 ur 24-01-2012 Hematology
.571 ps 29-02-2012 Hepato-billiaire Surgery
.341 ur 01-06-2011 Surgical reanimation
.643 cc 11-08-2012 Surgical reanimation
.400 cc 17-08-2011 Surgical reanimatiom
.542 ur 05-02-2012 Medical reanimation
.336 ps 24-05-2011 Orthopedic surgery
.404 cc 24-08-2011 Medical reanimation
.322 ur 09-05-2011 Medical reanimation
.302 di 11-04-2011 Nephrology
.278 ur 14-03-2011 Reeducation
.394 tr 14-08-2011 Reanimation medico-surgica emergency
.390 tr 08-08-2011 Medico-surgical emergency
.395 tr 14-08-2011 Reanimation medico-surgica emergency
.469 ps 06-11-2011 Medico-surgical emergency
.662 ur 12-09-2012 Nephrology
.476 cc 16-11-2011 Cardiology
.369 ur 13-07-2011 Medical reanimation
.636 ps 01-08-2012 Neurosurgery
.693 sa 26-11-2012 Cardiac surgery

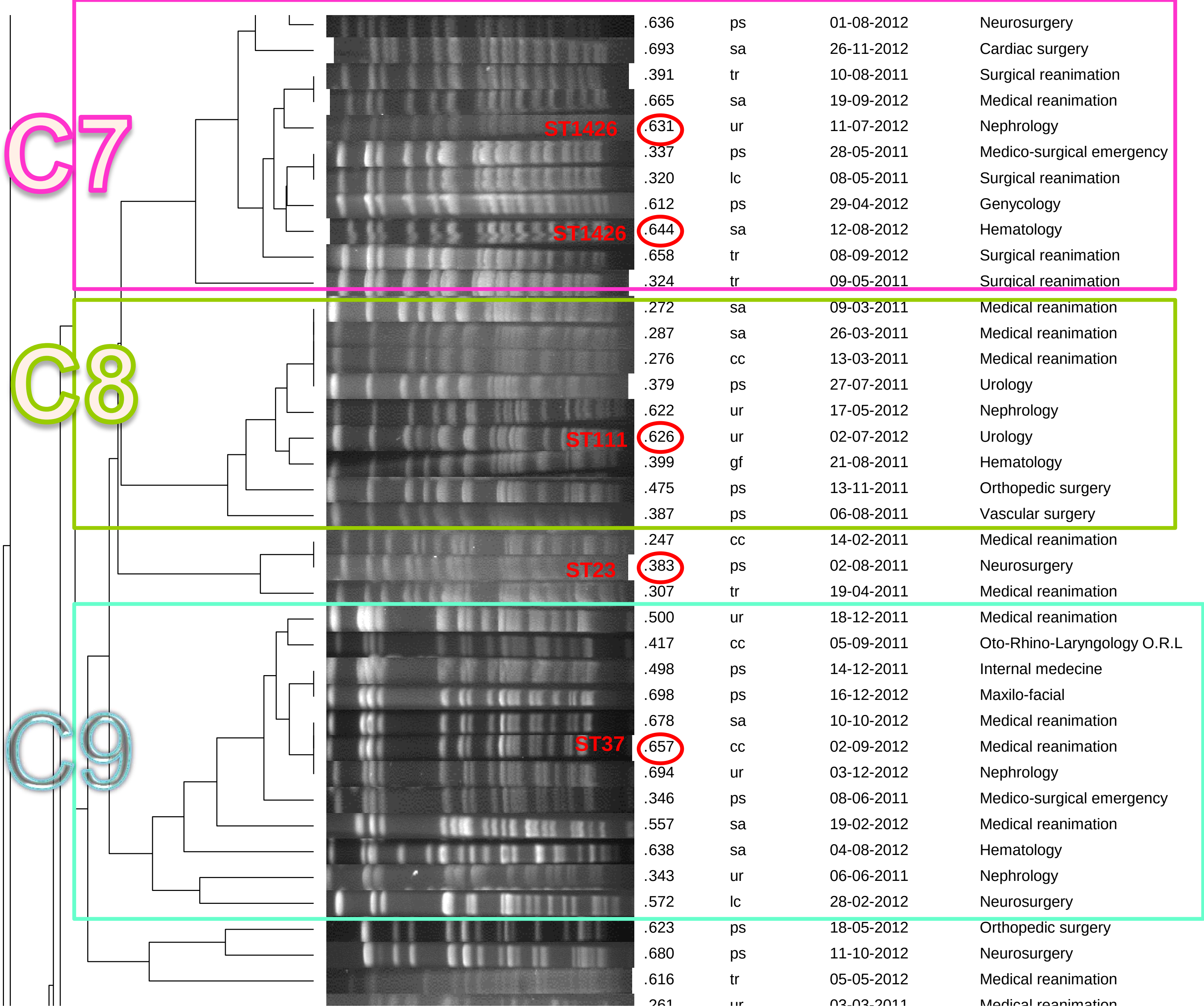
C7
C8
C9
ST1426
ST1426
ST111
ST23
ST37
.636 ps 01-08-2012 Neurosurgery
.693 sa 26-11-2012 Cardiac surgery
.391 tr 10-08-2011 Surgical reanimation
.665 sa 19-09-2012 Medical reanimation
.631 ur 11-07-2012 Nephrology
.337 ps 28-05-2011 Medico-surgical emergency
.320 lc 08-05-2011 Surgical reanimation
.612 ps 29-04-2012 Genycology
.644 sa 12-08-2012 Hematology
.658 tr 08-09-2012 Surgical reanimation
.324 tr 09-05-2011 Surgical reanimation
.272 sa 09-03-2011 Medical reanimation
.287 sa 26-03-2011 Medical reanimation
.276 cc 13-03-2011 Medical reanimation
.379 ps 27-07-2011 Urology
.622 ur 17-05-2012 Nephrology
.626 ur 02-07-2012 Urology
.399 gf 21-08-2011 Hematology
.475 ps 13-11-2011 Orthopedic surgery
.387 ps 06-08-2011 Vascular surgery
.247 cc 14-02-2011 Medical reanimation
.383 ps 02-08-2011 Neurosurgery
.307 tr 19-04-2011 Medical reanimation
.500 ur 18-12-2011 Medical reanimation
.417 cc 05-09-2011 Oto-Rhino-Laryngology O.R.L
.498 ps 14-12-2011 Internal medecine
.698 ps 16-12-2012 Maxilo-facial
.678 sa 10-10-2012 Medical reanimation
.657 cc 02-09-2012 Medical reanimation
.694 ur 03-12-2012 Nephrology
.346 ps 08-06-2011 Medico-surgical emergency
.557 sa 19-02-2012 Medical reanimation
.638 sa 04-08-2012 Hematology
.343 ur 06-06-2011 Nephrology
.572 lc 28-02-2012 Neurosurgery
.623 ps 18-05-2012 Orthopedic surgery
.680 ps 11-10-2012 Neurosurgery
.616 tr 05-05-2012 Medical reanimation
.261 ur 03-03-2011 Medical reanimation

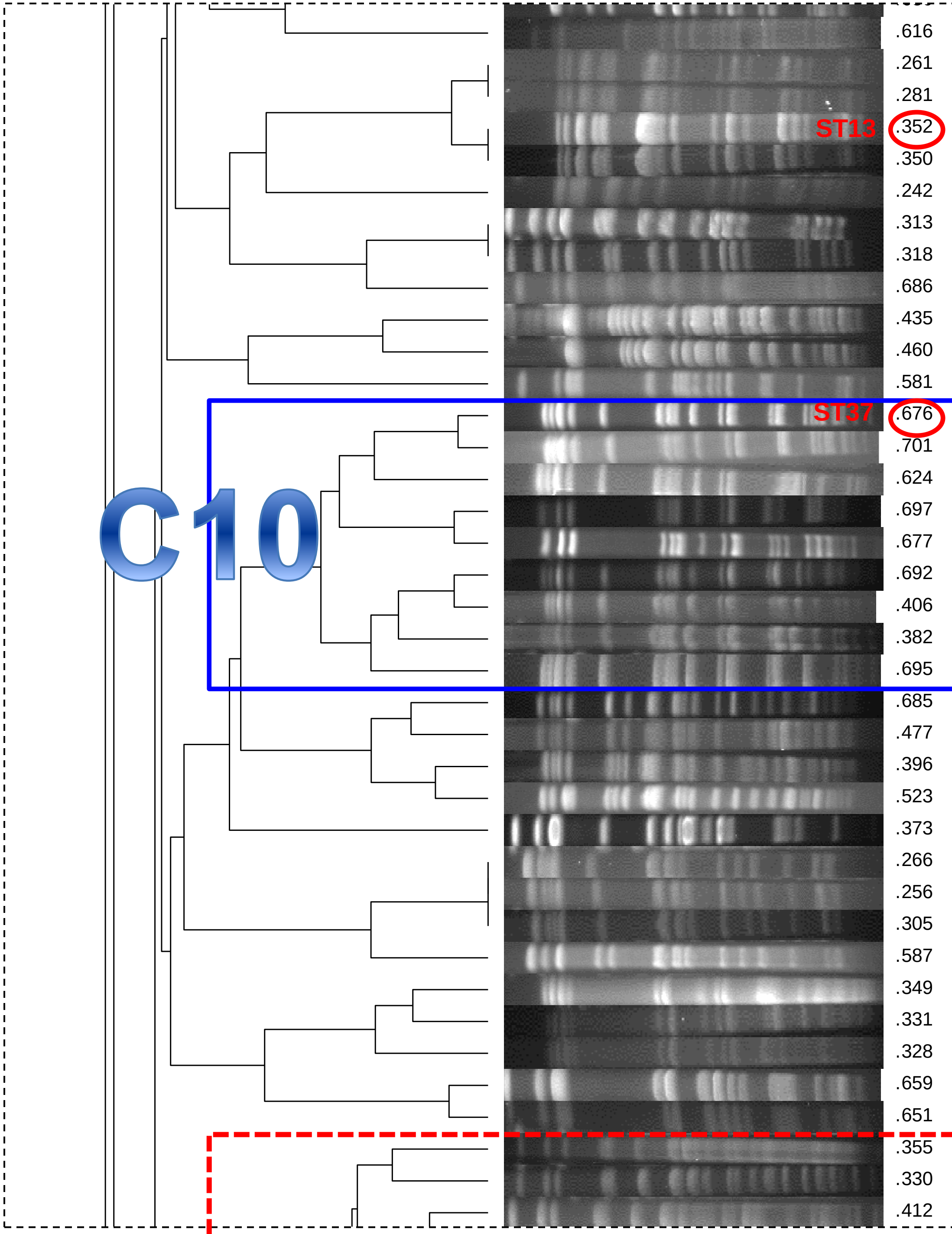

[illegible]	ps	[illegible]	[illegible]
.616	tr	05-05-2012	Medical reanimation
.261	ur	03-03-2011	Medical reanimation
.281	gf	21-03-2011	Medical reanimation
.352	cc	13-06-2011	Medical reanimation
.350	ps	12-06-2011	Medical reanimation
.242	ur	14-02-2011	Surgical reanimation
.313	ps	24-04-2011	Hepato-billiaire Surgery
.318	ps	08-05-2012	Surgical reanimation
.686	lc	21-10-2012	Neurosurgery
.435	ps	22-09-2011	Vascular surgery
.460	ps	23-10-2011	General surgery
.581	ps	19-03-2012	Vascular surgery
.676	ur	30-09-2012	Vascular surgery
.701	ab	23-12-2012	Medico-surgical emergency
.624	ur	15-05-2012	Gastrology
.697	ps	18-12-2012	Vascular surgery
.677	sa	01-10-2012	Reanimation medico-surgical emergency
.692	ur	25-11-2012	Reeducation
.406	ur	22-08-2011	Medical reanimation
.382	ps	02-08-2011	Internal medecine
.695	ps	03-12-2012	Neurosurgery
.685	ur	23-10-2012	Nephrology
.477	cc	23-11-2011	Reeducation
.396	ur	16-08-2011	Nephrology
.523	ur	15-01-2012	Nephrology
.373	sa	20-07-2011	Surgical reanimation
.266	ps	05-03-2011	Vascular surgery
.256	ps	03-09-2011	Surgical reanimation
.305	tr	13-04-2011	Surgical medical emergency
.587	ps	28-03-2012	Orthopedic surgery
.349	cc	12-06-2011	Reanimation medico-surgical emergency
.331	cc	22-05-2011	Reanimation medico-surgical emergency
.328	tr	16-05-2011	Surgical reanimation
.659	sa	09-09-2012	Medical reanimation
.651	ur	23-08-2012	Medical reanimation
.355	ur	20-06-2011	Medical reanimation
.330	ur	21-05-2011	Medical reanimation
.412	ps	03-09-2011	Surgical reanimation

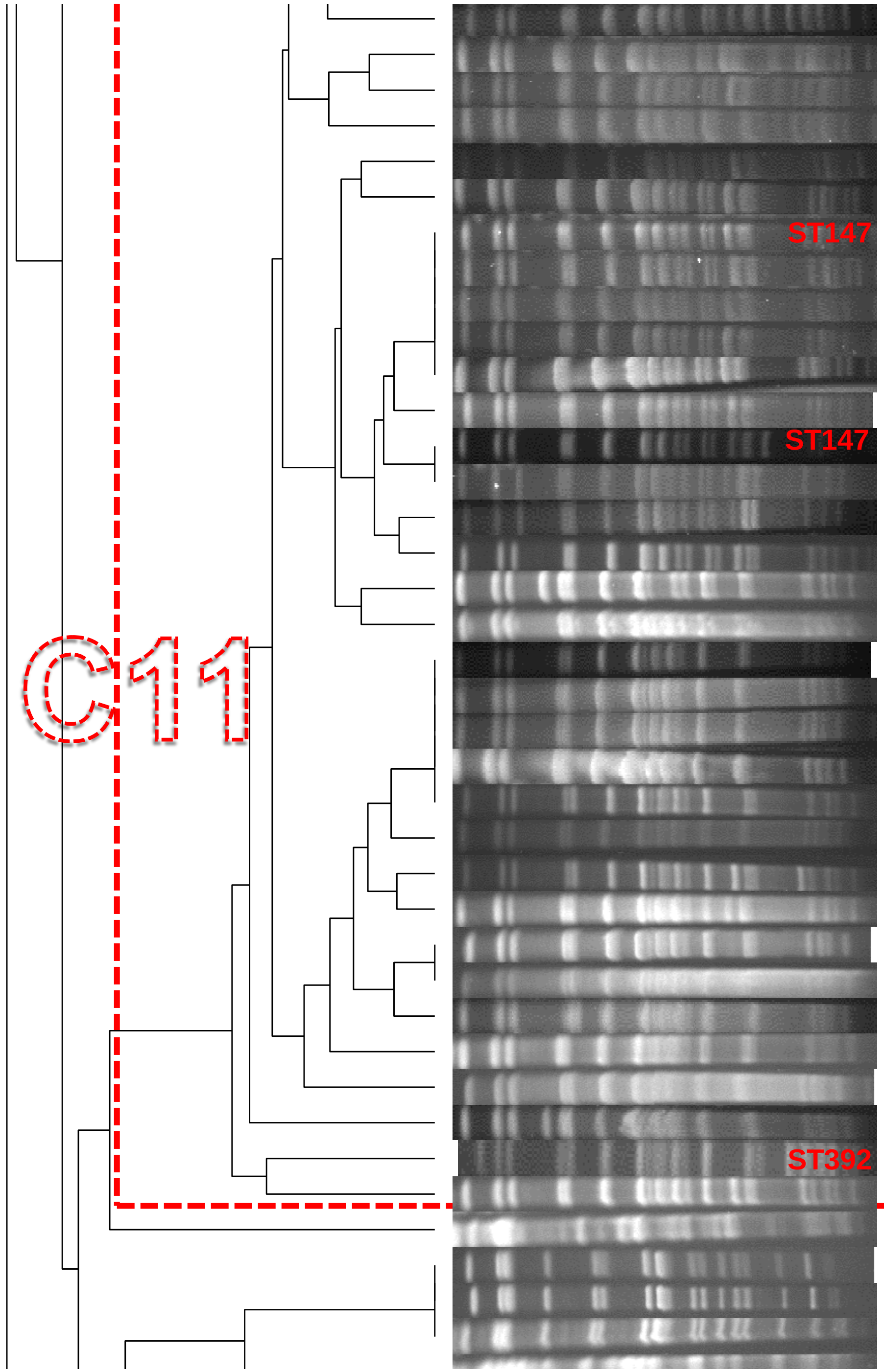

.330	ur	21-05-2011	Medical reanimation
.412	ps	03-09-2011	Surgical reanimation
.244	sa	12-02-2011	Medical reanimation
.245	cc	11-02-2011	Medical reanimation
.430	ps	14-09-2011	Vascular surgery
.436	ab	18-09-2011	Nephrology
.246	cc	13-02-2011	Surgical reanimation
.249	ur	12-02-2011	Surgical reanimation
.255	sa	14-02-2011	Cardiology
.228	ps	16-01-2011	Medico-surgical emergency
.494	ur	07-12-2011	Nephrology
.393	cc	14-08-2011	Surgical reanimation
.601	ur	18-04-2012	Nephrology
.251	sa	17-02-2011	Medical reanimation
.304	cc	11-04-2011	Medical reanimation
.688	ps	08-11-2012	Orthopedic surgery
.606	ps	22-04-2012	Orthopedic surgery
.586	cc	29-03-2012	Medico-surgical emergency
.679	ur	11-10-2012	Nephrology
.486	tr	05-12-2011	Medical reanimation
.474	cc	14-11-2011	Nephrology
.442	sa	03-10-2011	Reanimation medico-surgical emergency
.555	ur	21-02-2012	Medical reanimation
.633	sa	27-07-2012	Nephrology
.670	tr	30-09-2012	Surgical reanimation
.613	ps	29-04-2012	Reeducation
.573	lc	06-03-2012	Neurosurgery
.582	ur	18-03-2012	Nephrology
.449	ps	10-10-2011	Vascular surgery
.574	ab	08-03-2012	Gastrology
.529	ps	20-10-2012	Orthopedic surgery
.683	ut	23-10-2012	Nephrology
.640	ur	05-08-2012	Medical reanimation
.576	ps	11-03-2012	Reanimation medico-surgical emergency
.551	cc	19-02-2012	Surgical reanimation
.539	lc	02-02-2012	Neonatal
.521	lc	10-01-2012	Neonatal
.538	lc	31-01-2012	Neonatal
.546	ur	18-02-2012	Nephrology

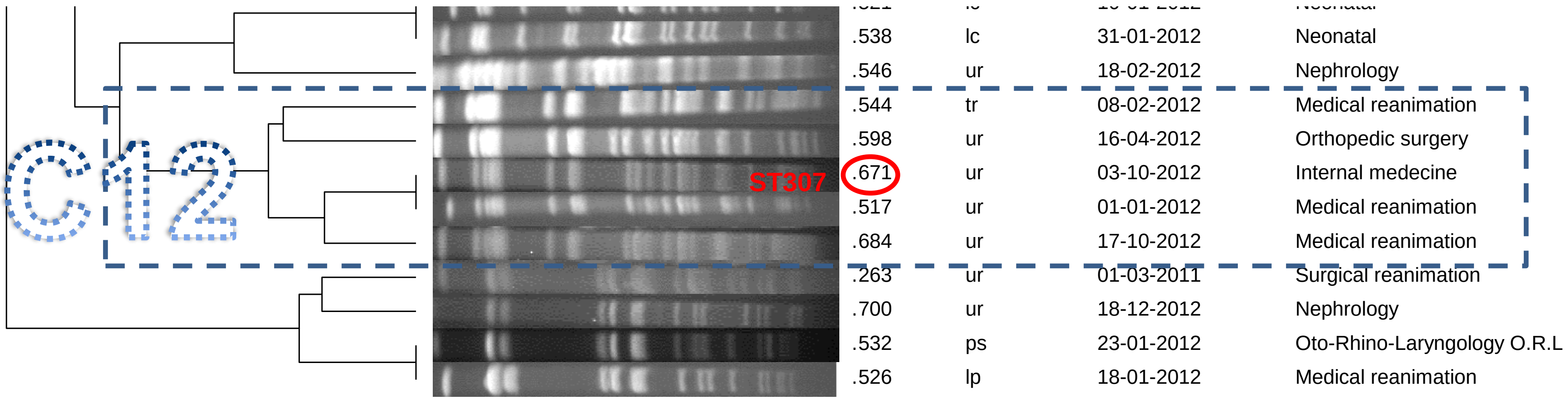

C12
ST307
.538 lc 31-01-2012 Neonatal
.546 ur 18-02-2012 Nephrology
.544 tr 08-02-2012 Medical reanimation
.598 ur 16-04-2012 Orthopedic surgery
.671 ur 03-10-2012 Internal medecine
.517 ur 01-01-2012 Medical reanimation
.684 ur 17-10-2012 Medical reanimation
.263 ur 01-03-2011 Surgical reanimation
.700 ur 18-12-2012 Nephrology
.532 ps 23-01-2012 Oto-Rhino-Laryngology O.R.L
.526 lp 18-01-2012 Medical reanimation
