# Supplementary material for: High-risk clones of extended-spectrum β-lactamase-producing Klebsiella pneumoniae isolated from the University Hospital Establishment of Oran, Algeria (2011–2012)
Source: PLoS One. 2021 Jul 26;16(7):e0254805. doi: 10.1371/journal.pone.0254805 (PMC8312963; doi:10.1371/journal.pone.0254805)
Supplement: S1 Table — B: G or T or C, M: A or C, V: G or A or C, H: A or T or C, S: G or C, Y: T or C. (DOCX) [file pone.0254805.s003.docx]

| Group | Target genes | Primers | Sequences 5’→ 3’ | References |
| --- | --- | --- | --- | --- |
| CTX | *bla_CTX-M all_* | CTX-M F | SCS ATG TGC AGY ACC AGT AA | [18] |
|  |  | CTX-M R | ACC AGA AYV AGC GGB GC |  |
|  | *bla_CTX+M-1 group_* | CTX-M-1 F | AAA AAT CAC TGC GCC AGT TC | [19] |
|  |  | CTX-M-1 R | AGC TTA TTC ATC GCC ACG TT |  |
|  | *bla_CTX+M-2 group_* | CTX-M-2 F | CGA CGC TAC CCC TGC TAT T |  |
|  |  | CTX-M-2 R | CCA GCG TCA GAT TTT TCA GG |  |
|  | *bla_CTX+M-9 group_* | CTX-M-9 F | CAA AGA GAG TGC AAC GGA TG |  |
|  |  | CTX-M-9 R | ATT GGA AAG CGT TCA TCA CC |  |
| PMQR | *qnrA* | qnrA F | GGG TAT GGA TAT TAT TGA TAA AG | [20] |
|  |  | qnrA R | CTA ATC CGG CAG CAC TAT TA |  |
|  | *qnrB* | qnrB F | GGM ATH GAA ATT CGC CAC TG |  |
|  |  | qnrB R | TTT GCY GYY CGC CAG TCG AA |  |
|  | *qnrC* | qnrC F | GAT TTT TCC GGC CAA GAT TT |  |
|  |  | qnrC R | TAA CAA TCA CCC CCA ACT GC |  |
|  | *qnrD* | qnrD F | CGA GAT CAA TTT ACG GGG AAT |  |
|  |  | qnrD R | CGG TGA ACA ATA ACA CCT AAA CTC |  |
|  | *qnrS* | qnrS F | AGT GAT CTC ACC TTC ACC GC |  |
|  |  | qnrS R | CAG GCT GCA ATT TTG ATA CC |  |
|  | *qepA* | qepA F | GGA CAT CTA CGG CTT CTT CG |  |
|  |  | qepA R | GGT GAT GAT GAT CTC GTT GC |  |
|  | *aac(6)–Ib* | aac(6)F | TGA CCA ACA GCA ACG ATT CC | [21] |
|  |  | aac(6)R | TTA GGC ATC ACT GCG TGT TC |  |
| 16S rRNA methylases genes | *armA* | armA_F | AGG TTG TTT CCA TTT CTG AG | [22] |
|  |  | armA_R | TCT CTT CCA TTC CCT TCT CC |  |
|  | *rmtA* | rmtA_F | CTA GCG TCC ATC CTT TCC TC |  |
|  |  | rmtA_R | TTT GCT TCC ATG CCC TTG CC |  |
|  | *rmtB* | rmtB_F | CCC AAA CAG ACC GTA GAG GC |  |
|  |  | rmtB_R | CTC AAA CTC GGC GGG CAA GC |  |
|  | *rmtC* | rmtC_F | CGA AGA AGT AAC AGC CAA AG |  |
|  |  | rmtC_R | ATC CCA ACA TCT CTC CCA CT |  |
|  | *rmtD* | rmtD_F | GAG CGA ACT GAA GGA AAA AC |  |
|  |  | rmtD_R | CAG CAC GTA AAA CAG CTC |  |
