# Supplementary material for: High-risk clones of extended-spectrum β-lactamase-producing Klebsiella pneumoniae isolated from the University Hospital Establishment of Oran, Algeria (2011–2012)
Source: PLoS One. 2021 Jul 26;16(7):e0254805. doi: 10.1371/journal.pone.0254805 (PMC8312963; doi:10.1371/journal.pone.0254805)
Supplement: S3 Table — A: Salmonella breakpoints. (DOC) [file pone.0254805.s005.doc]

| **Antibiotics** | **%R** | **%I** | **%S** |
| --- | --- | --- | --- |
| **Ampicillin** | 100 | 0 | 0 |
| **Amoxicillin/clavulanic acid** | 16.6 | 47.7 | 35.8 |
| **Ceftazidime** | 83.9 | 13 | 3.1 |
| **Cefotaxime** | 99.5 | 0 | 0.5 |
| **Cefoxitin** | 3.6 | 3.6 | 92.7 |
| **Aztreonam** | 73 | 22.3 | 4.7 |
| **Ertapenem** | 1 | 1.6 | 97.4 |
| **Imipenem** | 0 | 1.1 | 98.9 |
| **Meropenem** | 0 | 0.5 | 99.5 |
| **Amikacin** | 20.2 | 14 | 65.8 |
| **Gentamicin** | 91.2 | 0 | 8.8 |
| **Nalidixic acid** | 42.5 | 20.7 | 36.8 |
| **Ciprofloxacin** | 76 | 4 | 20 |
| **Pefloxacin^A^** | 77.5 | 0 | 22.5 |
| **Trimethoprim-sulfamethoxazole** | 81.4 | 3.5 | 15.2 |
