# Supplementary material for: High-risk clones of extended-spectrum β-lactamase-producing Klebsiella pneumoniae isolated from the University Hospital Establishment of Oran, Algeria (2011–2012)
Source: PLoS One. 2021 Jul 26;16(7):e0254805. doi: 10.1371/journal.pone.0254805 (PMC8312963; doi:10.1371/journal.pone.0254805)
Supplement: S4 Table — AMC: Amoxicillin/clavulanic acid, CAZ: Ceftazidime, CTX: Cefotaxime, CEF: Cefepime, FOX: Cefoxitin, ERT: Ertapenem, IMP: Imipenem, MEM: Meropenem, AMK: Amikacin, GEN: Gentamicin, NAL: Nalidixic acid, CIP: Ciprofloxacin, PEF: Pefloxacin. (DOCX) [file pone.0254805.s006.docx]

|  | Pulsotypes | | | | |  |
| --- | --- | --- | --- | --- | --- | --- |
| Resistance patterns | C1 | C7 | C8 | C9 | C11 | Total |
| CEP CTX CAZ PEF GEN | 7 | 7 | 6 | 1 | 0 | 21 |
| CEP CTX CAZ NAL PEF CIP GEN | 0 | 0 | 0 | 6 | 15 | 21 |
| CEP CTX NAL PEF CIP GEN | 0 | 0 | 0 | 0 | 8 | 8 |
| CEP CTX PEF GEN | 3 | 2 | 1 | 0 | 1 | 7 |
| CEP CTX CAZ PEF | 1 | 0 | 0 | 0 | 0 | 1 |
| CEP CTX CAZ PEF CIP | 1 | 0 | 0 | 0 | 0 | 1 |
| AMC CEP CTX NAL GEN | 1 | 0 | 0 | 0 | 0 | 1 |
| AMC CEF CTX CAZ PEF GEN | 1 | 0 | 0 | 0 | 0 | 1 |
| CEP CTX NAL PEF GEN | 1 | 1 | 0 | 0 | 0 | 2 |
| CEP CTX PEF CIP | 1 | 0 | 0 | 0 | 0 | 1 |
| AMC CEP CTX CAZ NAL PEF CIP GEN | 0 | 0 | 0 | 1 | 1 | 2 |
| AMC CEP CTX CAZ NAL AMK PEF CIP GEN | 0 | 0 | 0 | 0 | 1 | 1 |
| CEP CTX CAZ GEN | 0 | 0 | 1 | 0 | 0 | 1 |
| AMC CEP CTX CAZ IMP PEF MEM ERT GEN | 0 | 1 | 0 | 0 | 0 | 1 |
| CTX CAZ PEF CIP GEN | 0 | 1 | 0 | 0 | 0 | 1 |
| AMC CEP CTX CAZ NAL CIP GEN | 0 | 0 | 1 | 0 | 0 | 1 |
| CEP CTX CAZ PEF CIP GEN | 0 | 0 | 1 | 0 | 0 | 1 |
| CEP FOX CTX CAZ NAL PEF CIP GEN | 0 | 0 | 0 | 1 | 0 | 1 |
| CTX CAZ PEF GEN | 0 | 0 | 0 | 1 | 0 | 1 |
| AMC CEP CTX CAZ PEF | 0 | 0 | 0 | 1 | 0 | 1 |
| AMC CEP FOX CTX CAZ NAL PEF CIP GEN | 0 | 0 | 0 | 0 | 1 | 1 |
| CEP CTX CAZ NAL PEF GEN | 0 | 0 | 0 | 0 | 2 | 2 |
| CEP CTX CAZ NAL CIP GEN | 0 | 0 | 0 | 0 | 1 | 1 |
| AMC CEF CTX CAZ NAL PEF CIP GEN | 0 | 0 | 0 | 0 | 1 | 1 |
| CEF CTX CAZ NAL PEF CIP GEN | 0 | 0 | 0 | 0 | 1 | 1 |
| CEP CTX CAZ NAL AMK PEF CIP GEN | 0 | 0 | 0 | 0 | 1 | 1 |
| CTX CAZ NAL AMK PEF CIP | 0 | 0 | 0 | 0 | 1 | 1 |
| AMC CEP FOX CTX CAZ NAL AMK PEF CIP GEN | 0 | 0 | 0 | 0 | 1 | 1 |
| Total | 16 | 12 | 10 | 11 | 35 | 84 |
