# Supplementary material for: High-risk clones of extended-spectrum β-lactamase-producing Klebsiella pneumoniae isolated from the University Hospital Establishment of Oran, Algeria (2011–2012)
Source: PLoS One. 2021 Jul 26;16(7):e0254805. doi: 10.1371/journal.pone.0254805 (PMC8312963; doi:10.1371/journal.pone.0254805)
Supplement: S1 Raw images — (PDF) [file pone.0254805.s007.pdf]

PEGE Gels, captured using GelDoc2

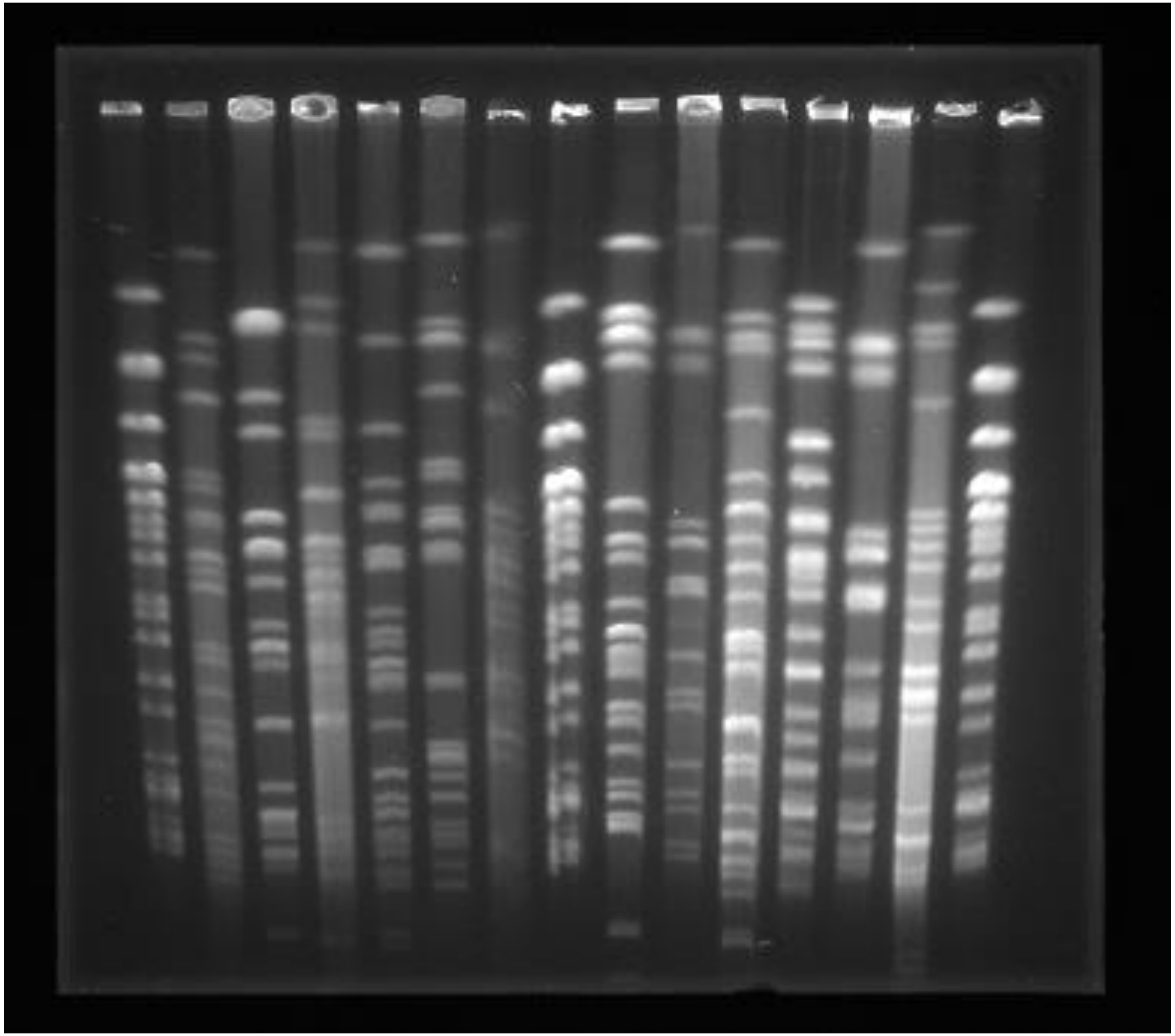

Gel 1:

From the right to the left

1. G5244
2. 601
3. 532
4. 685
5. 638
6. 700
7. 698
8. G5244
9. 527
10. 632
11. 622
12. 633
13. 623
14. 678
15. G5244

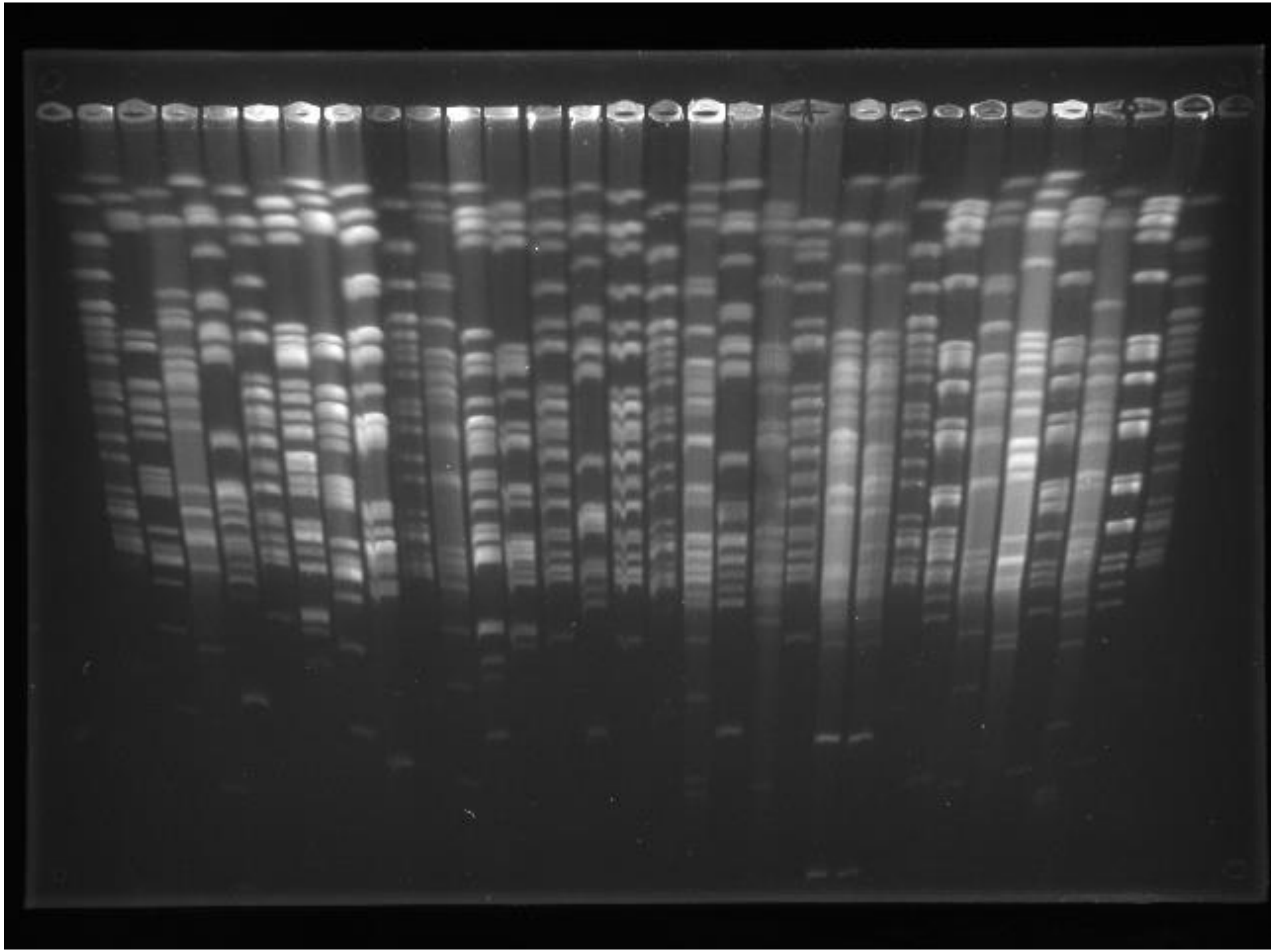

Gel 2

From the right to the left

1. G5244
2. 695
3. 616
4. 624
5. 699
6. 633
7. 676
8. G5244
9. 681
10. 687
11. 693
12. 692
13. 645
14. 640
15. G5244
16. 644
17. 675
18. 665
19. 697
20. 657
21. 679
22. G5244
23. 686
24. 659
25. 557
26. 631
27. 629
28. 663
29. 651
30. G5244

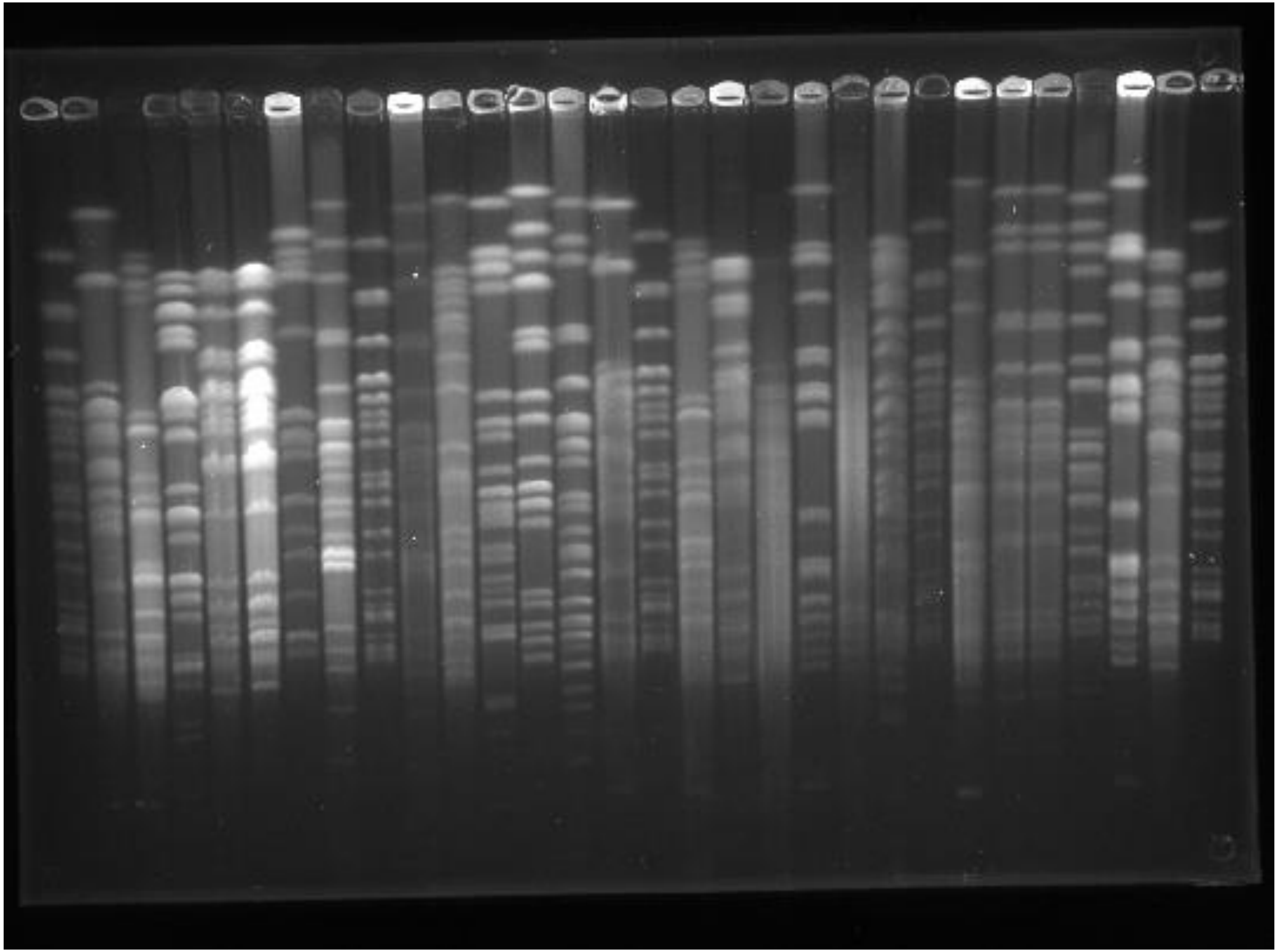

Gel 3

From de right to the left

1. G5244
2. 634
3. 661
4. 636
5. 679
6. 679
7. 687
8. G5244
9. 643
10. X
11. 628
12. X
13. 344
14. 328
15. G4255
16. 345
17. 330
18. X
19. 346
20. 302
21. 304X
22. G5244
23. 304
24. 305
25. 316X
26. 316
27. 350
28. 331
29. 348
30. G5244

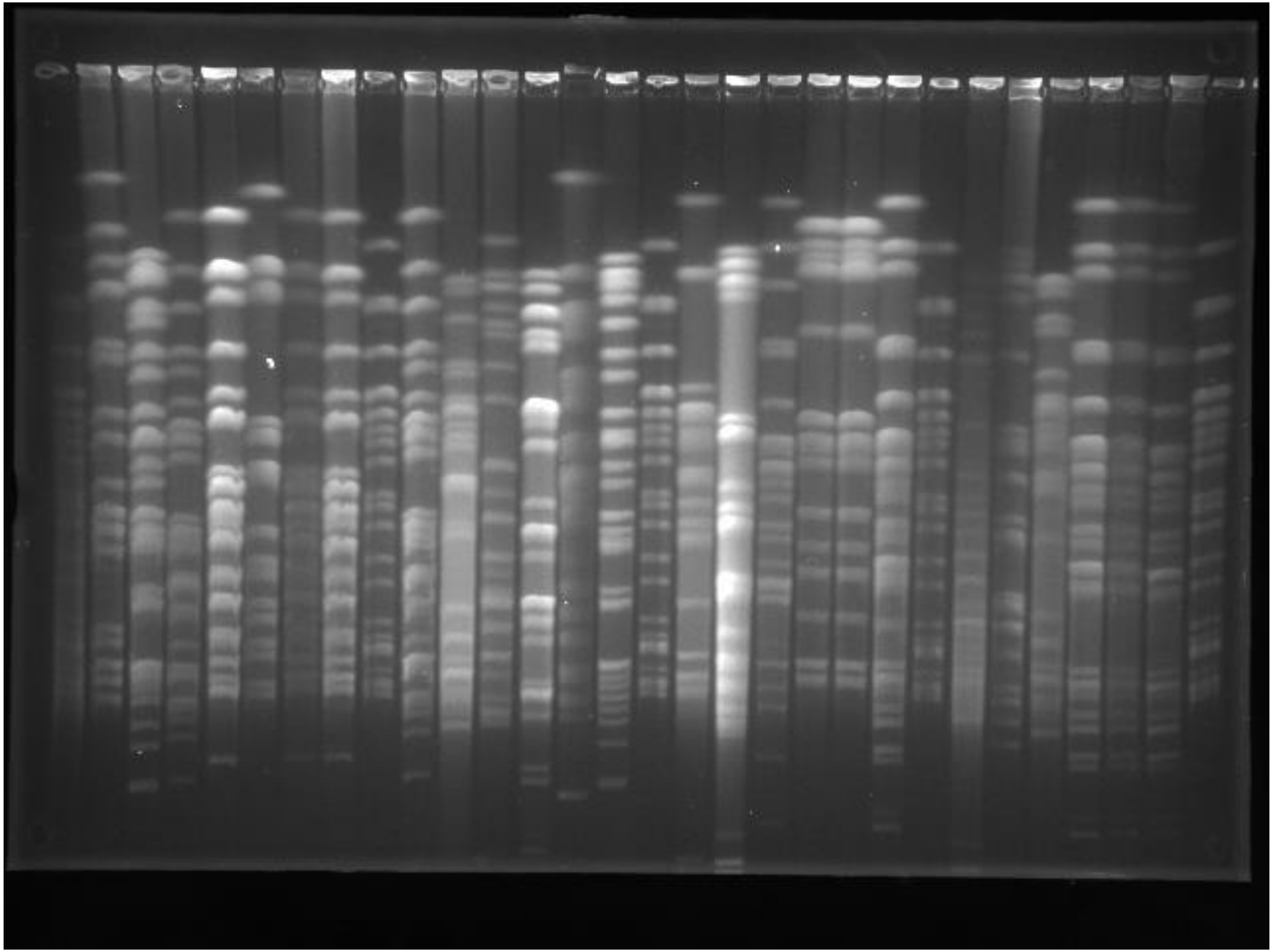

Gel 4

From de right to the left

1. G5244
2. 430
3. 393
4. 393X
5. 408
6. 382
7. 394
8. G5244
9. 412
10. 266
11. 266X
12. 251
13. 349
14. 342
15. G5244
16. 341
17. 339
18. 352
19. 322
20. 306
21. 307
22. G5244
23. 320
24. 324
25. 343
26. 337
27. 311
28. 366
29. 313
30. G5244

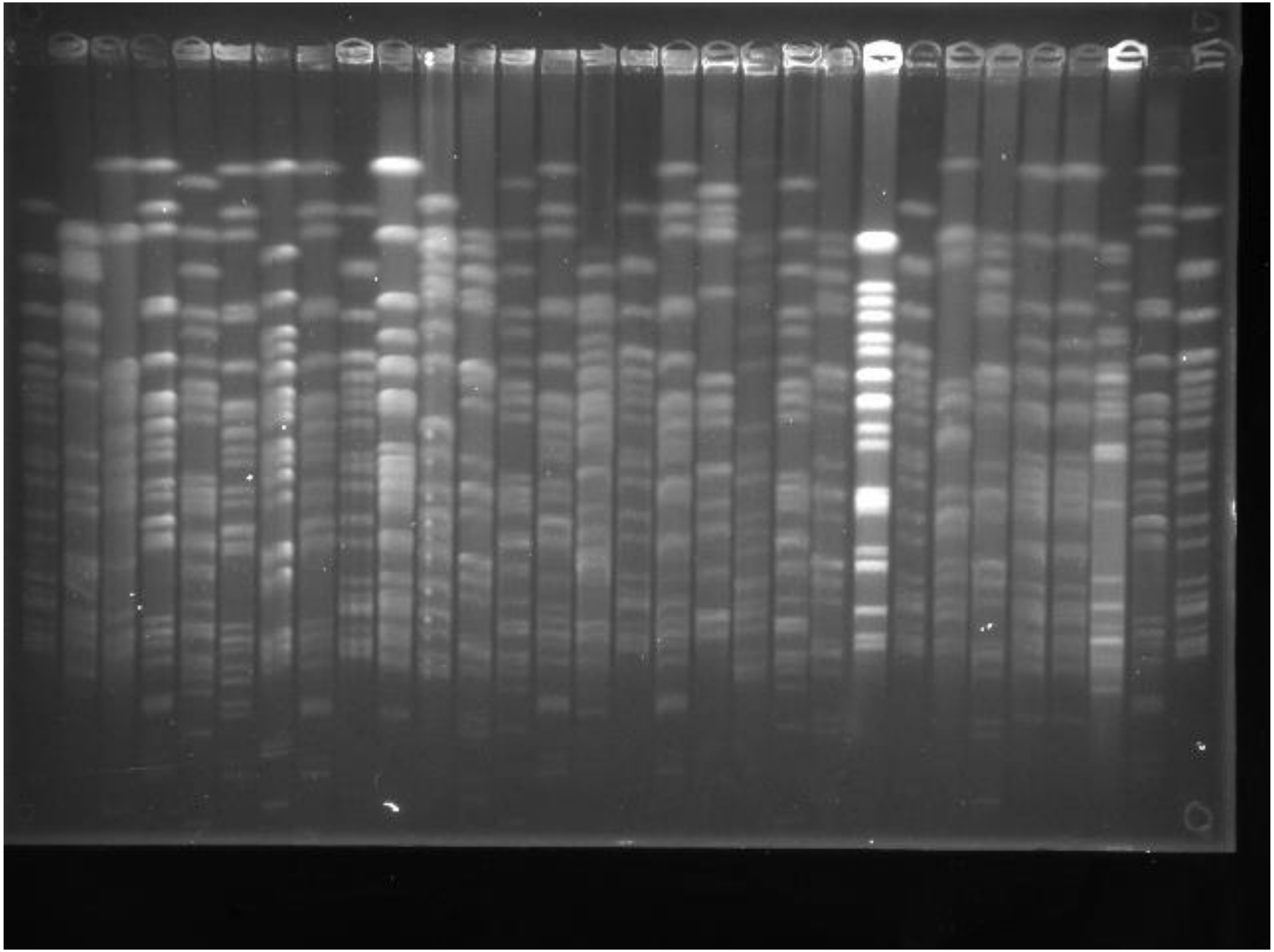

Gel 5

From the right to the left

1. G5244
2. 228
3. 283
4. 276
5. 287
6. 281
7. 263
8. G5244
9. 260
10. 261
11. 247
12. 278
13. 256
14. 245
15. G5244
16. 269
17. 244
18. 248
19. 285
20. 286
21. 272
22. G5244
23. 255
24. 279
25. 249
26. 268
27. 246
28. 257
29. 242
30. G4255

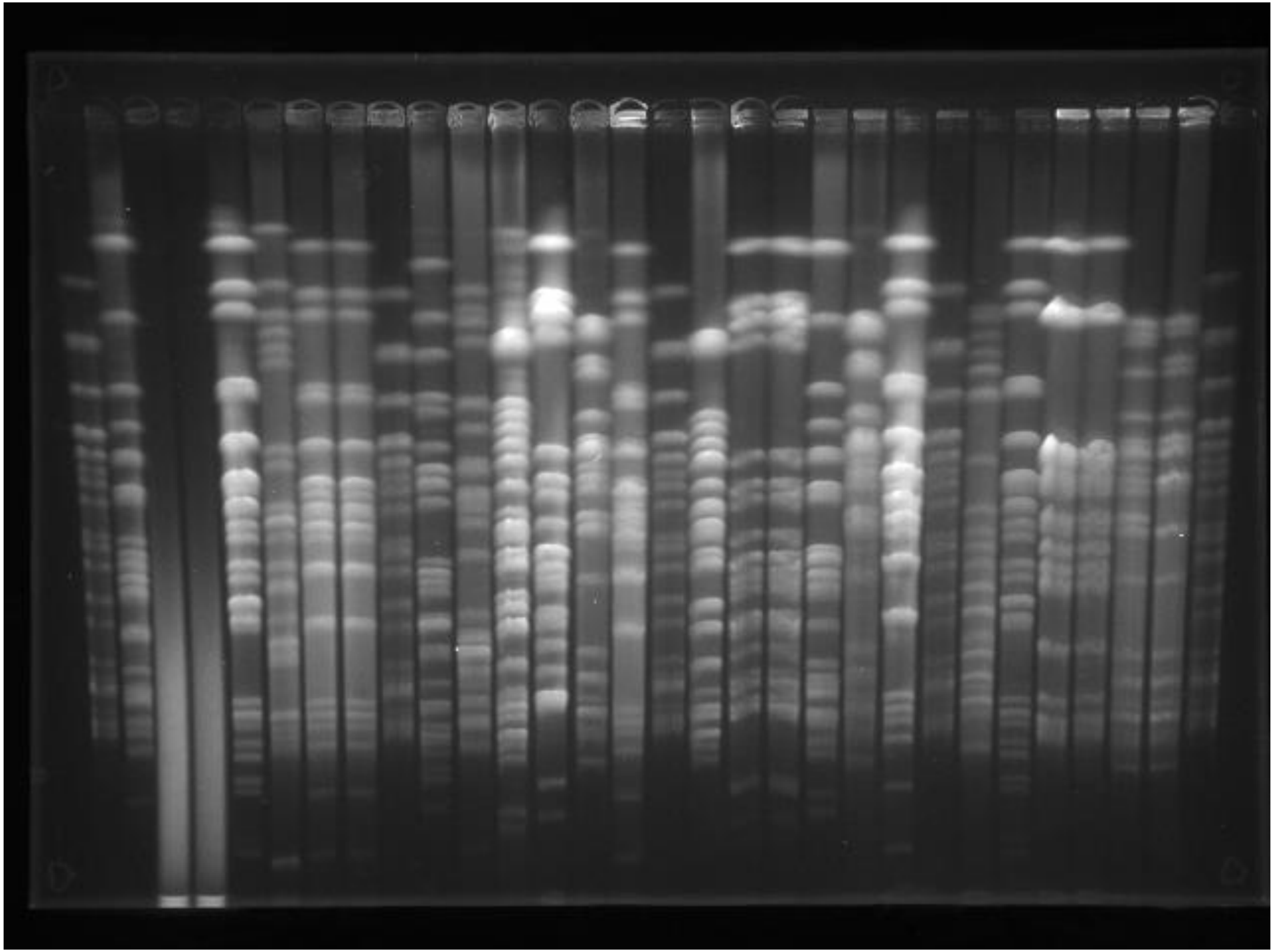

Gel 6

From the right to the left

1. G5244
2. 454
3. 464
4. 440
5. 440X
6. 436
7. 469
8. G5244
9. 442
10. 448
11. 475
12. 498X
13. 498
14. 460
15. G5244
16. 449
17. 431
18. 500
19. 435
20. 477
21. 471
22. G4255
23. 474
24. 486
25. 476
26. 494
27. 356X
28. 356X
29. 399
30. G5244

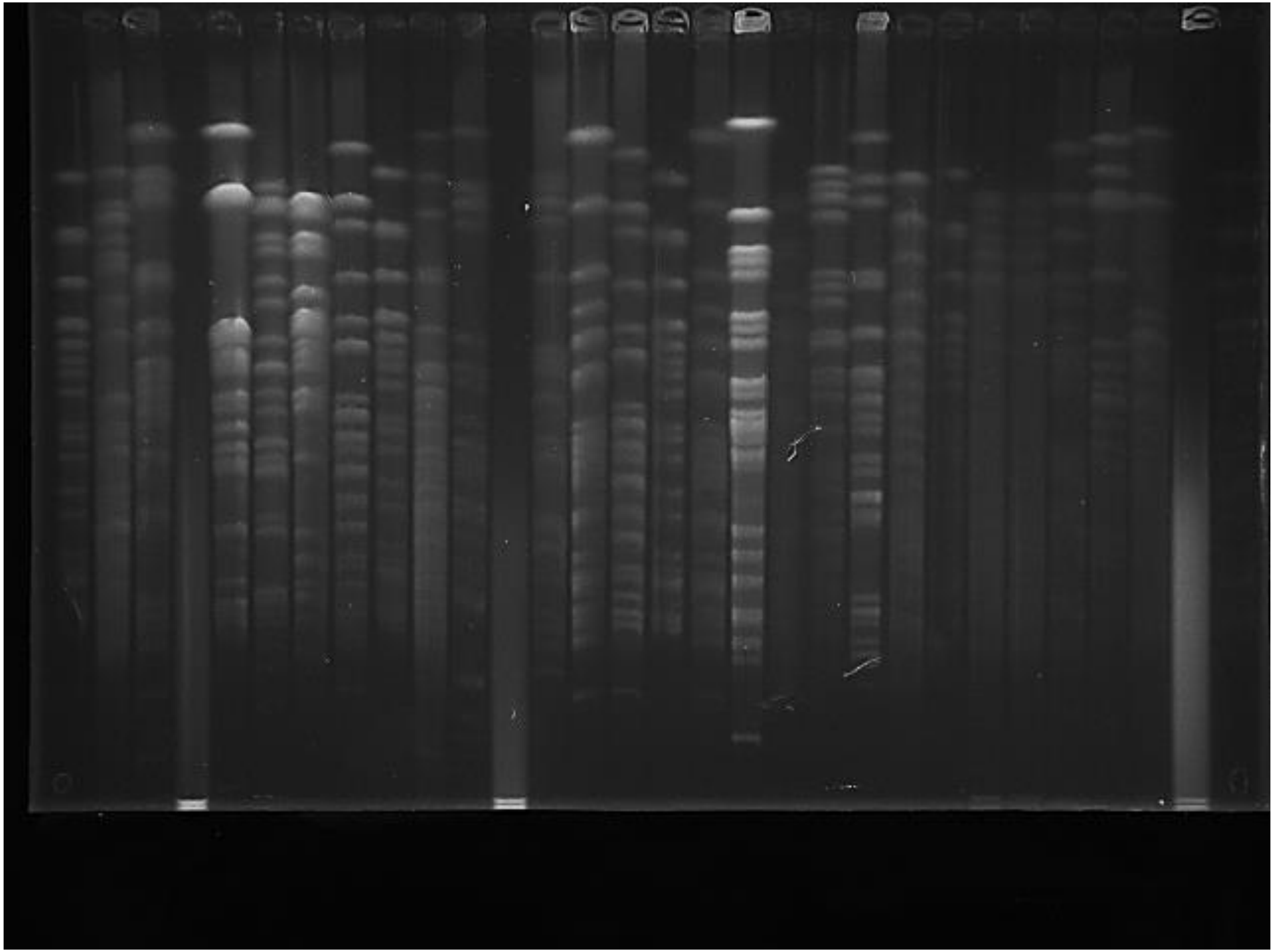

Gel 7

From the right to the left

1. G5244
2. 380X
3. 377X
4. 373X
5. 383X
6. 390X
7. 394X
8. G4255
9. 375
10. 393
11. 396
12. 429
13. 339
14. 379
15. G5244
16. 369
17. 387
18. 382
19. 380X
20. 417
21. 355
22. G5244
23. 368
24. 448
25. 400
26. 440
27. 356X
28. 442
29. 404
30. G5244

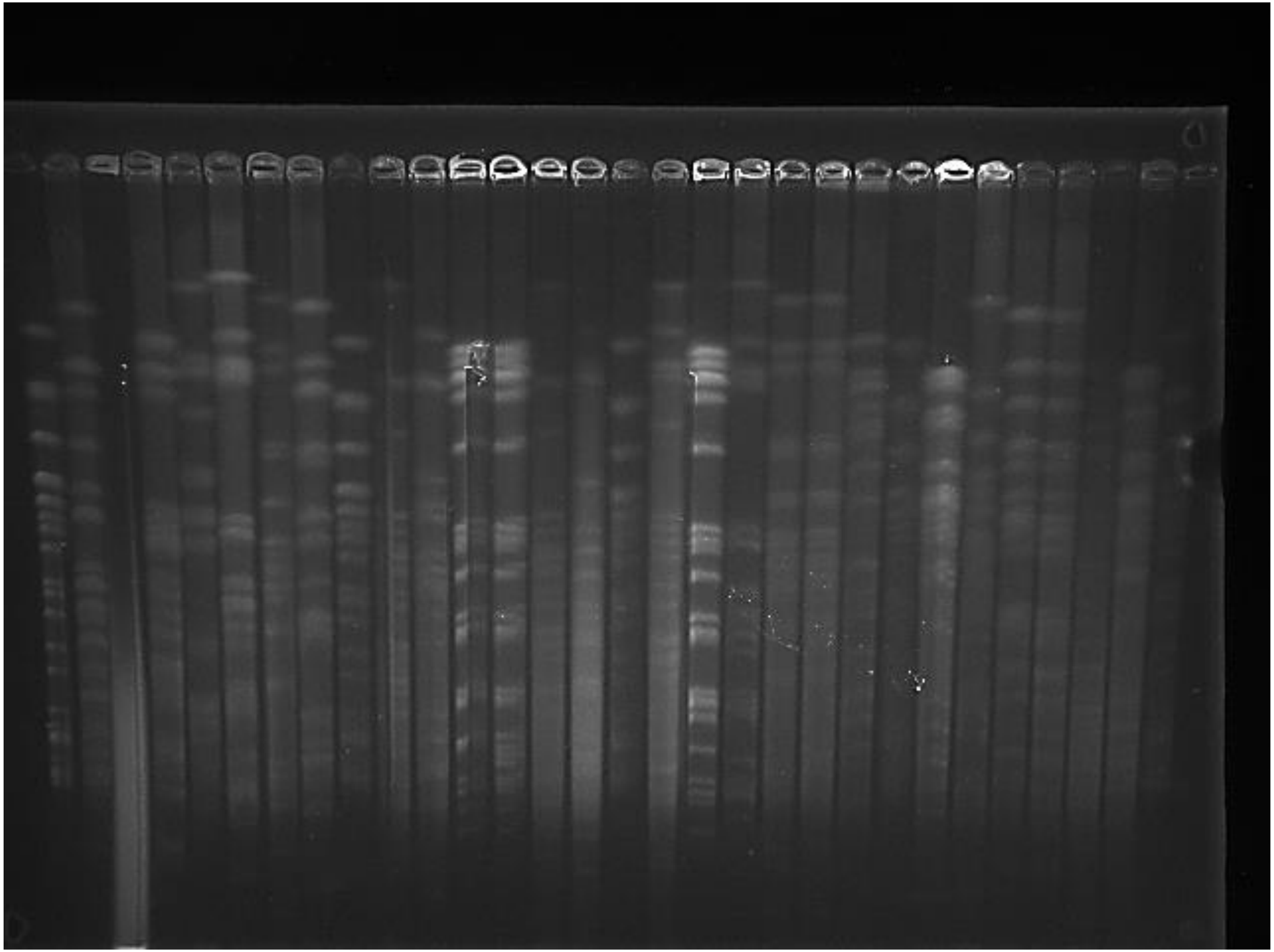

Gel 8

From de right to the left

1. G5244
2. X
3. X
4. X
5. X
6. X
7. X
8. G5244
9. X
10. X
11. X
12. X
13. X
14. X
15. G5244
16. 616
17. 687
18. 676
19. 695
20. 663
21. 681
22. G5244
23. 686
24. 679
25. 659
26. 629
27. 557
28. 380X
29. 631
30. G5244

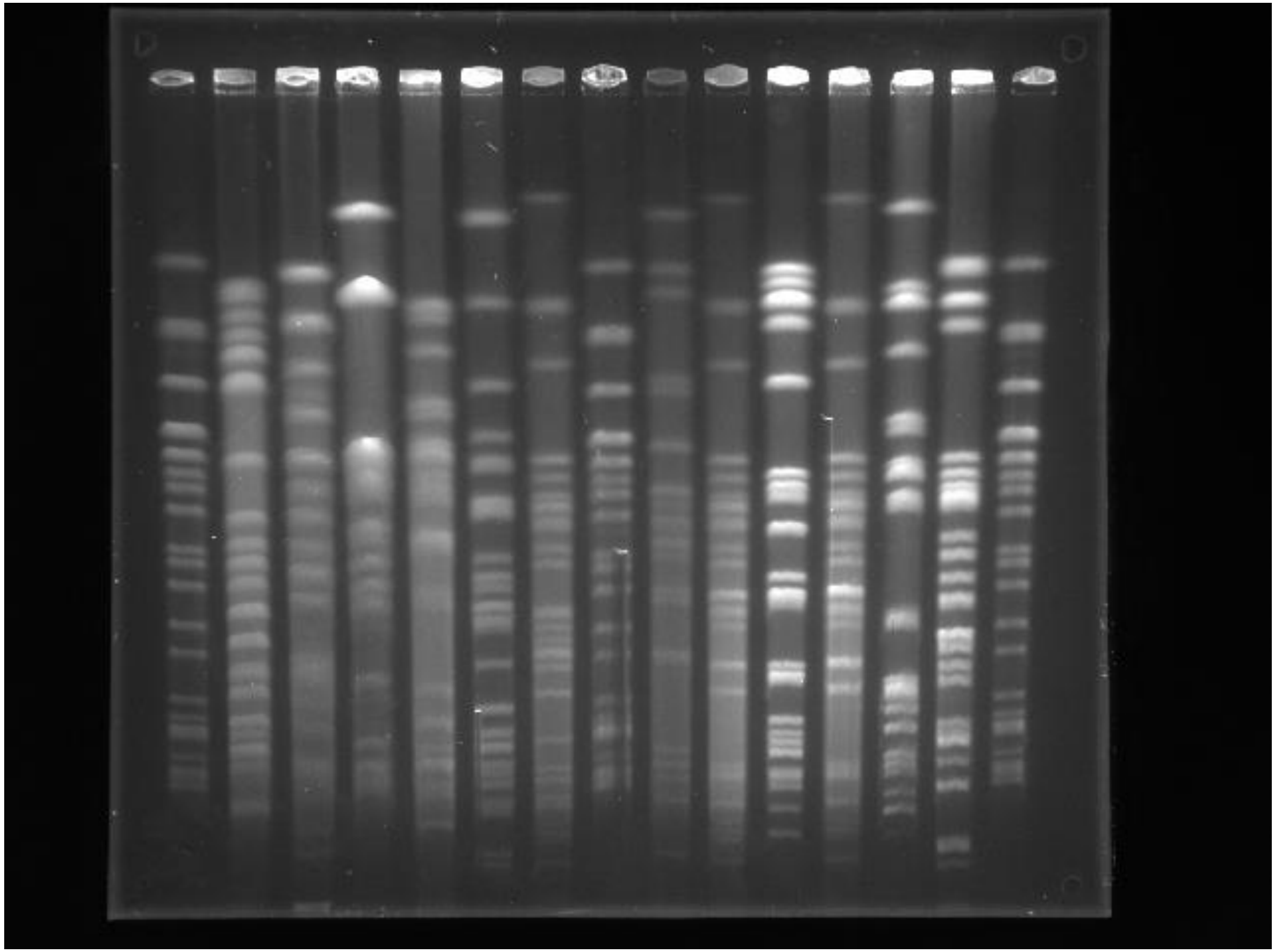

Gel 9

From de right to the left

1. G5244
2. 557
3. 629
4. 687
5. 676
6. 687X
7. 633
8. G5244
9. 681
10. 622
11. 306
12. 440
13. 375
14. 395
15. G5244

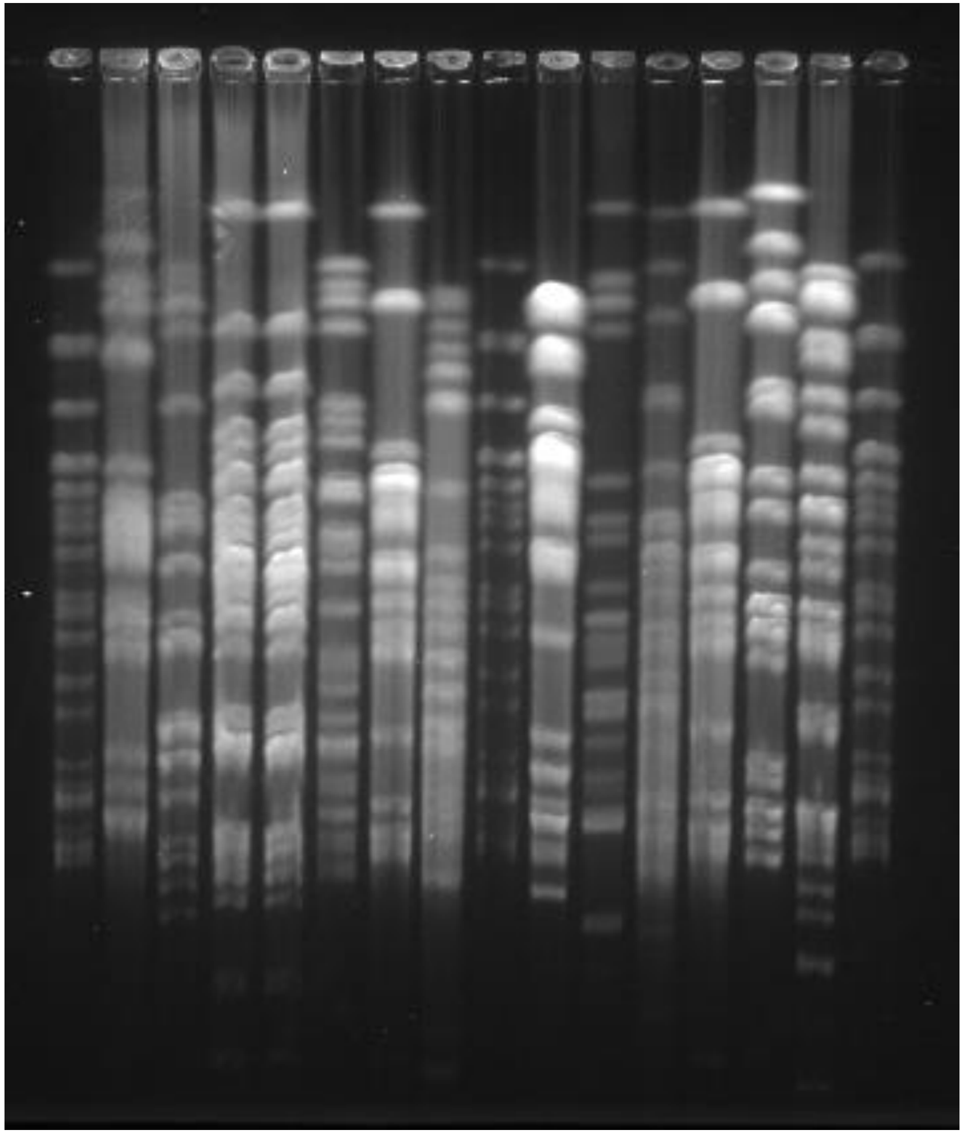

Gel 10

From the right to the left

1. G5244
2. 336
3. 313
4. 348
5. 355
6. 417
7. 448
8. G5244
9. 394
10. 377
11. 396
12. 366
13. 366
14. 382
15. 376
16. G5244

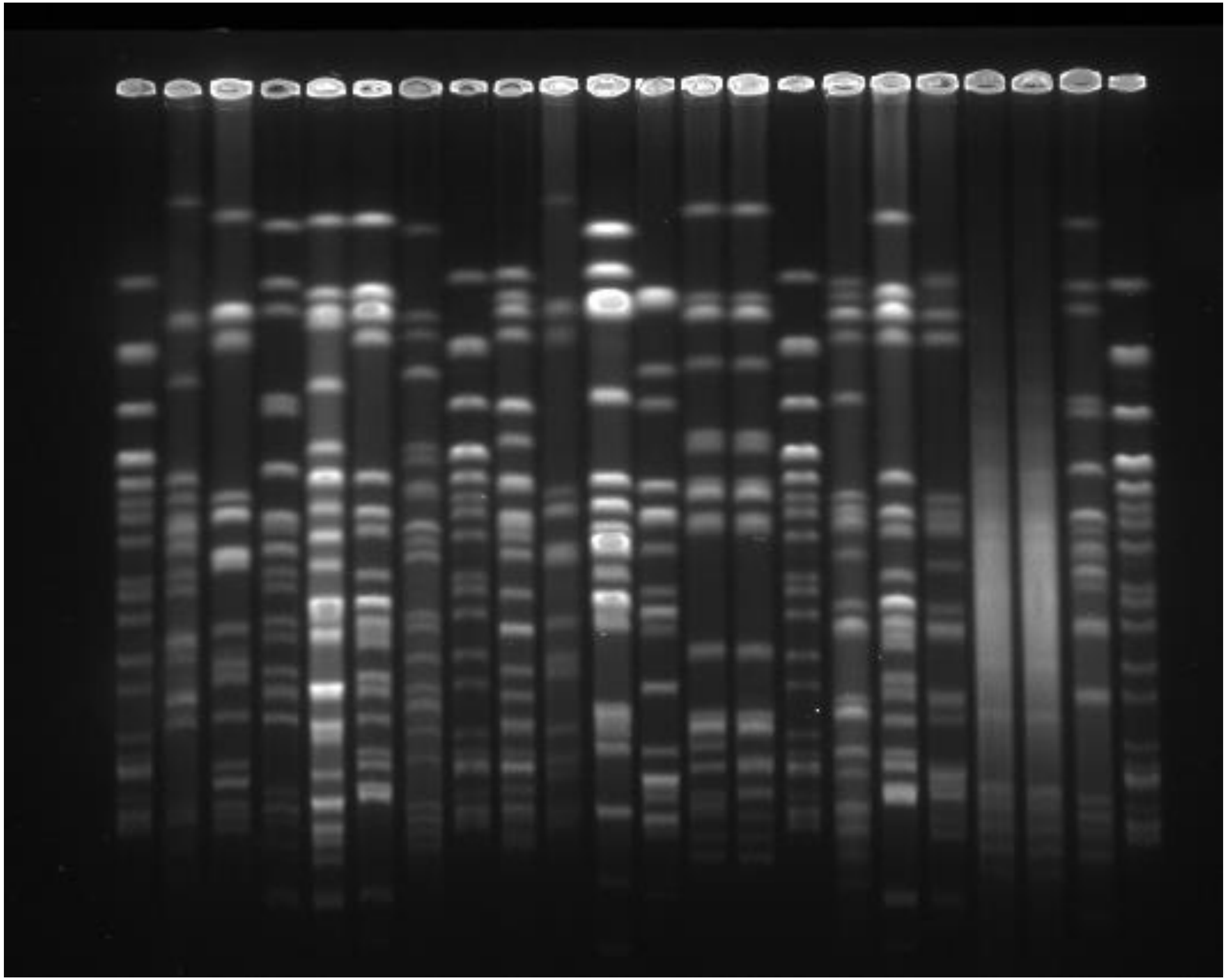

Gel 11

From the right to the left

1. G5244
2. 679
3. 666X
4. 666X
5. 697
6. 659
7. 692
8. G5244
9. 645
10. 632
11. 623
12. 373
13. 700
14. 685
15. G5244
16. 698
17. 678
18. 638
19. 601
20. 532
21. 527
22. G5244

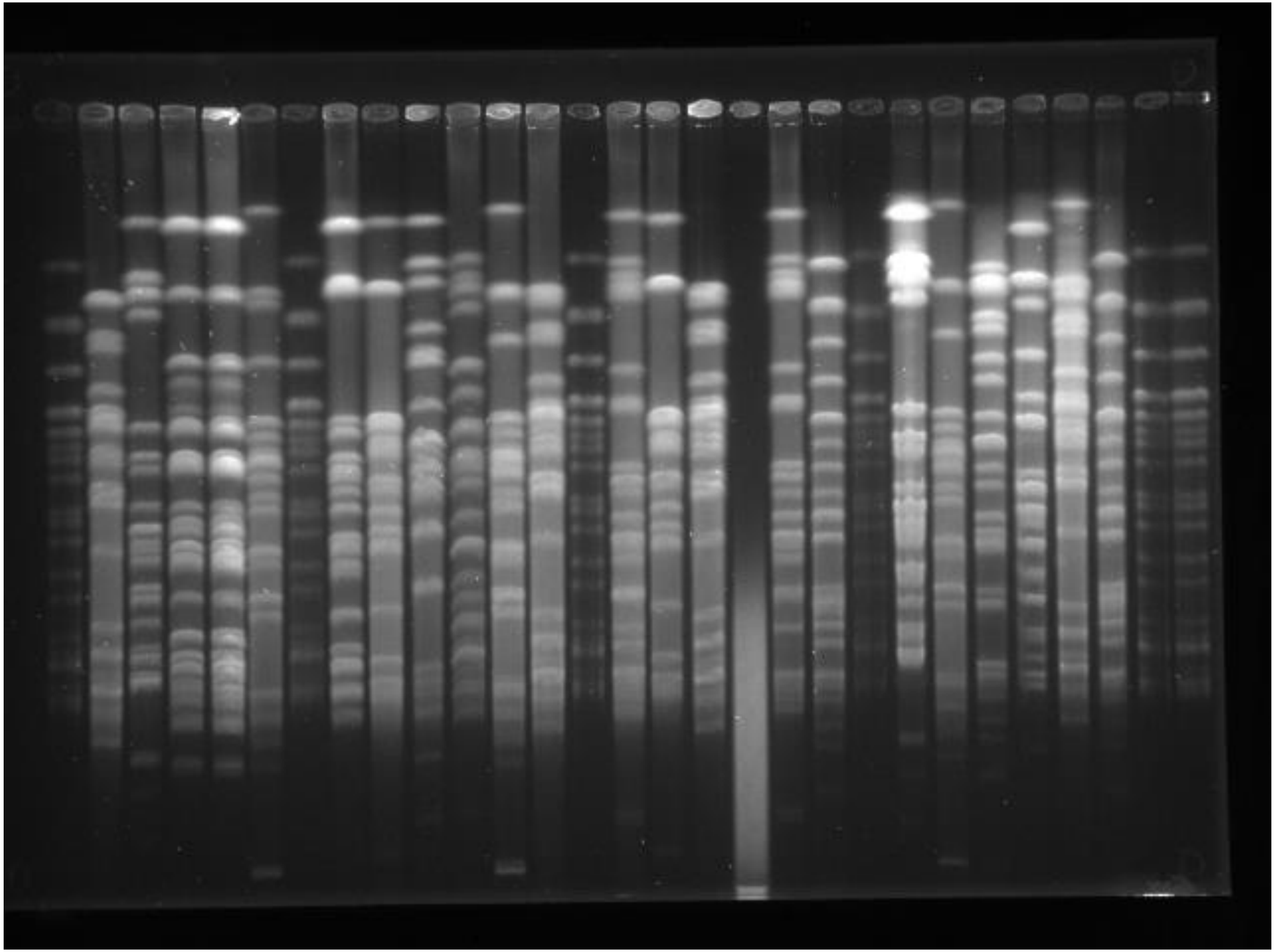

Gel 12

From the right to the left

1. G5244
2. G5244
3. 385
4. 674
5. 658
6. 571
7. 699
8. 694X
9. G5244
10. 385X
11. 671
12. 380X
13. 674
14. 440
15. 684
16. G5244
17. 672
18. 656
19. 685
20. 683
21. 257
22. 416
23. G5244
24. 690
25. 626X
26. 626
27. 694
28. 545
29. G5244

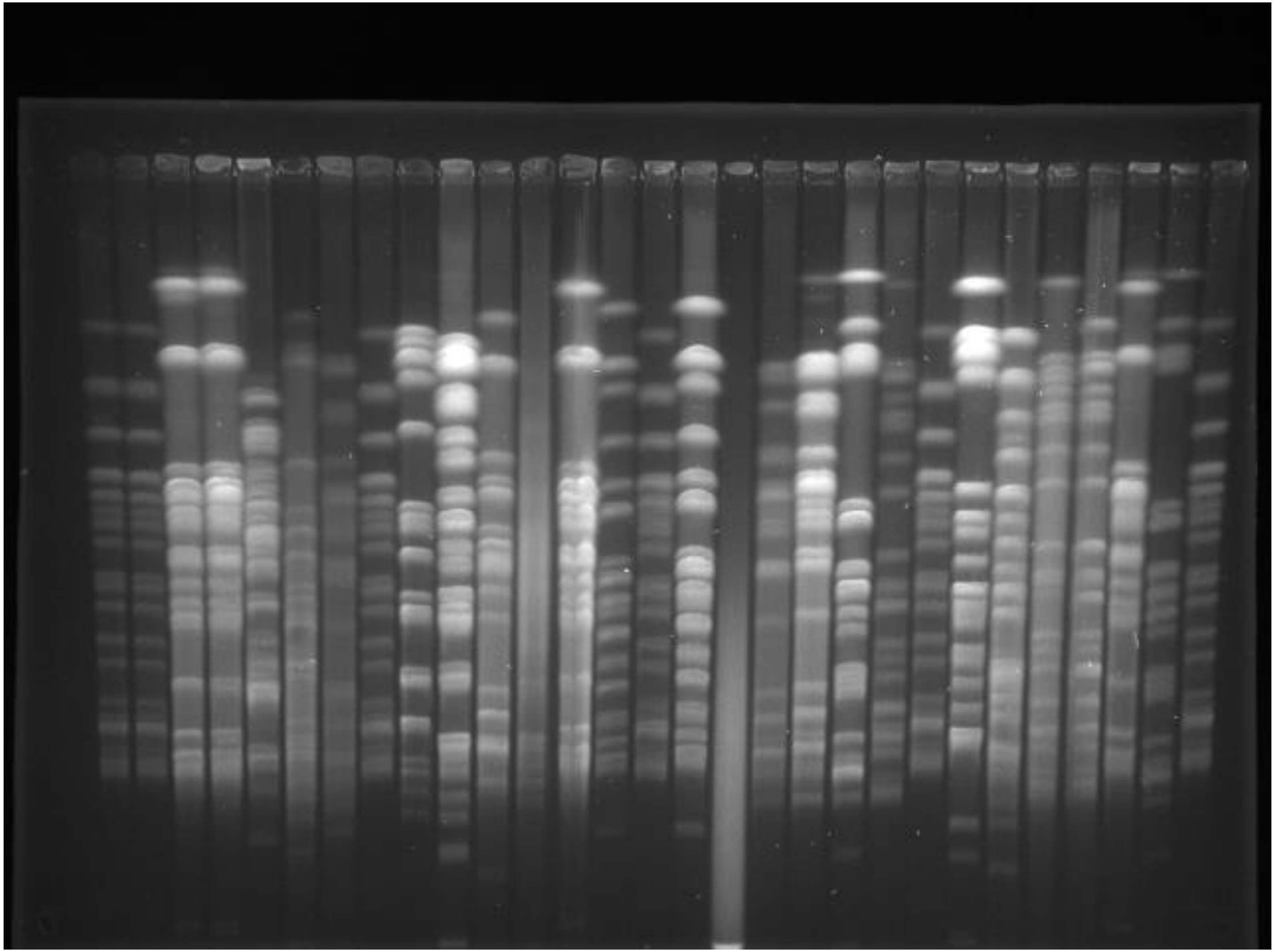

Gel 13

From the right to the left

1. G5244
2. 651
3. 377
4. 404
5. 302
6. 375
7. 500
8. G5244
9. 278
10. 659
11. 408
12. 283
13. 356X
14. 324
15. G5244
16. 391
17. 348X
18. 666X
19. 663
20. 695
21. 429
22. G5244
23. 616
24. 269
25. 345
26. 347
27. G5244
28. G5244

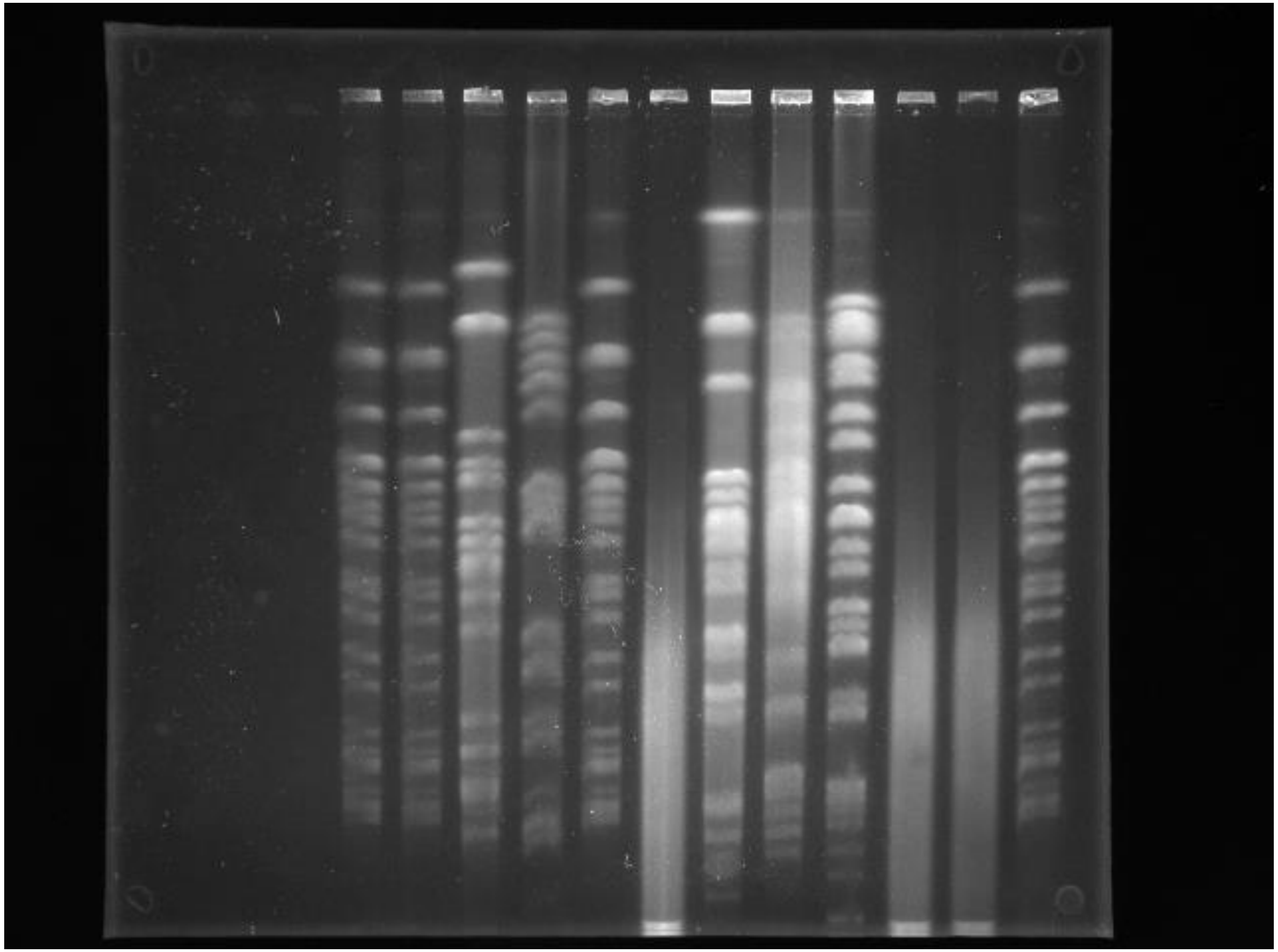

Gel 14

From the right to the left

1. G5244
2. 380X
3. 380X
4. 643
5. 666X
6. 669
7. 356X
8. G5244
9. 662
10. 663
11. G5244
12. G5244

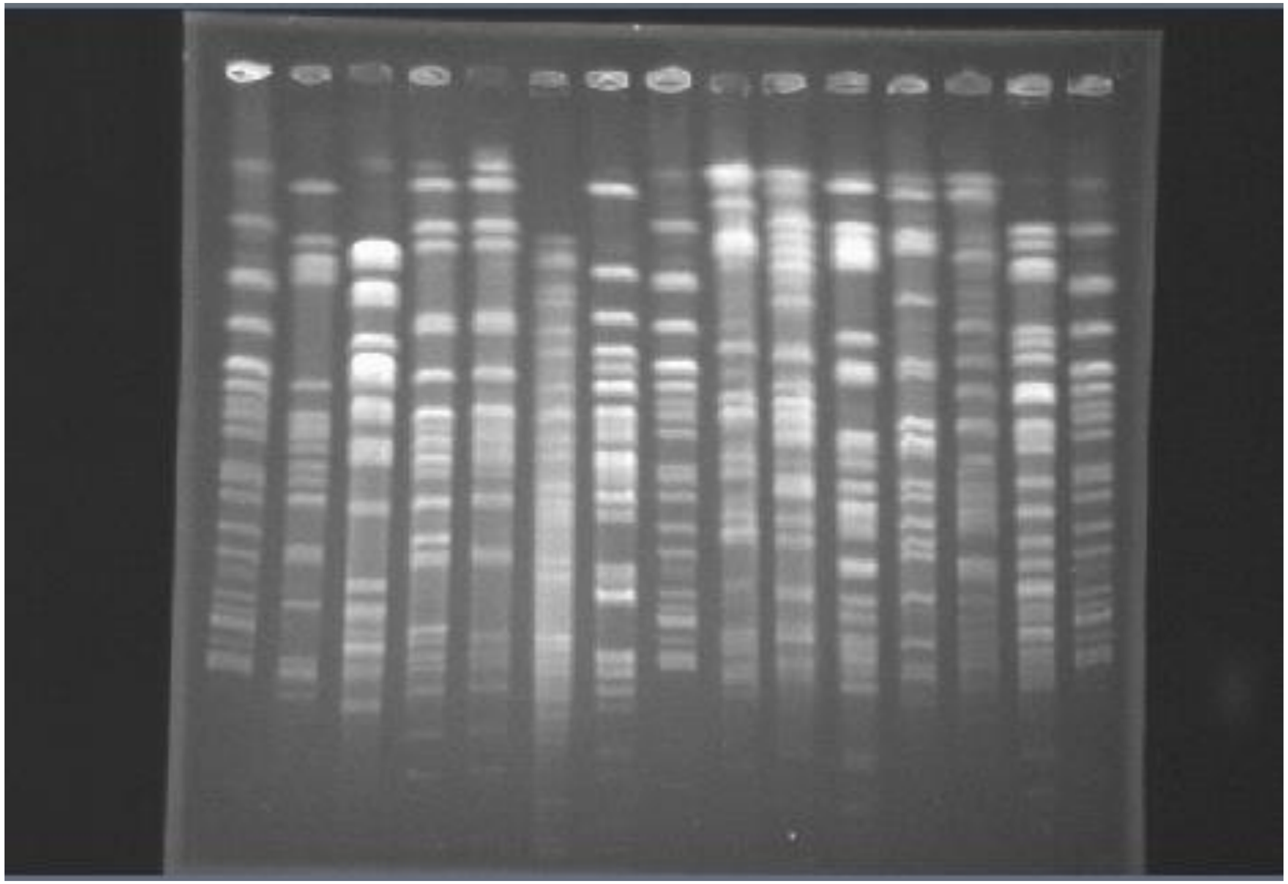

Gel 15

From the right to the left

1. G5244
2. 523
3. 533
4. 538
5. 544
6. 546
7. 551
8. G5244
9. 561
10. 571
11. 574
12. 576
13. 579
14. 581
15. G5244

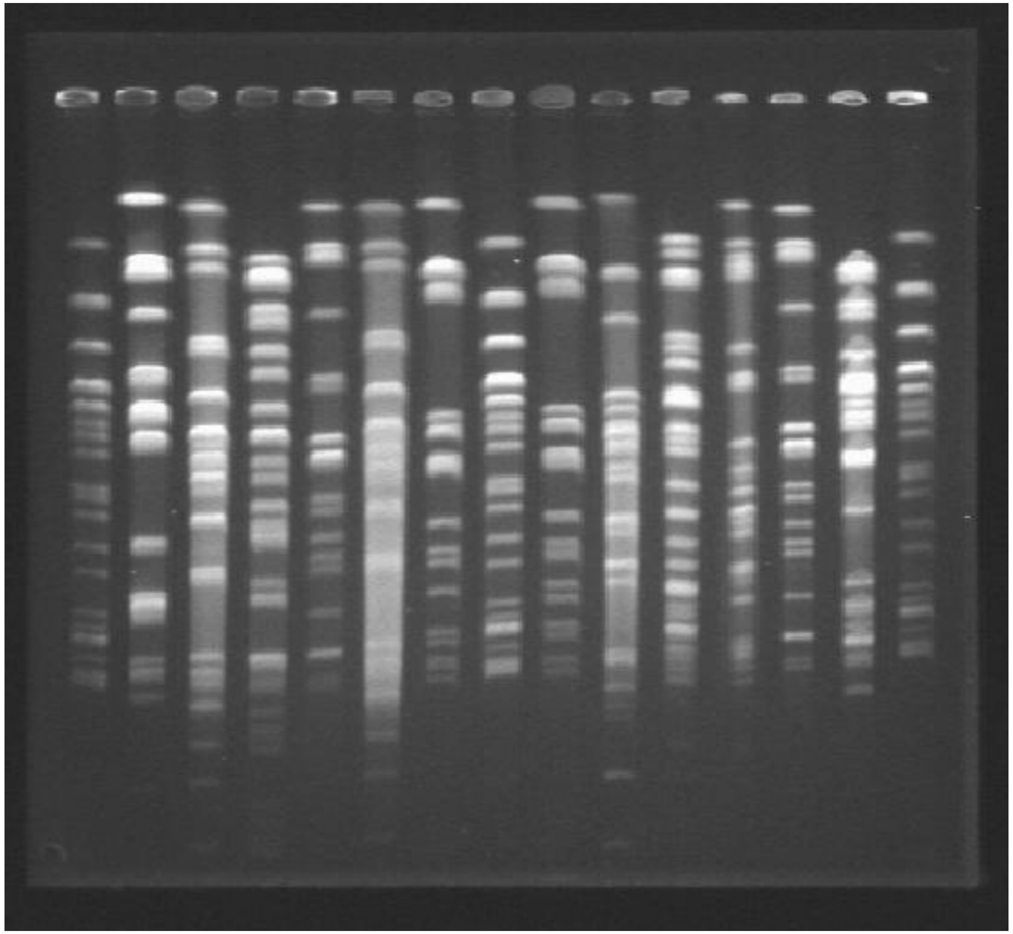

Gel 16:
From the left to the right

1. G5244
2. 689
3. 573
4. 542
5. 539
6. 529
7. 526
8. G5244
9. X
10. 527
11. 523
12. 517
13. 521
14. 518
15. G5244

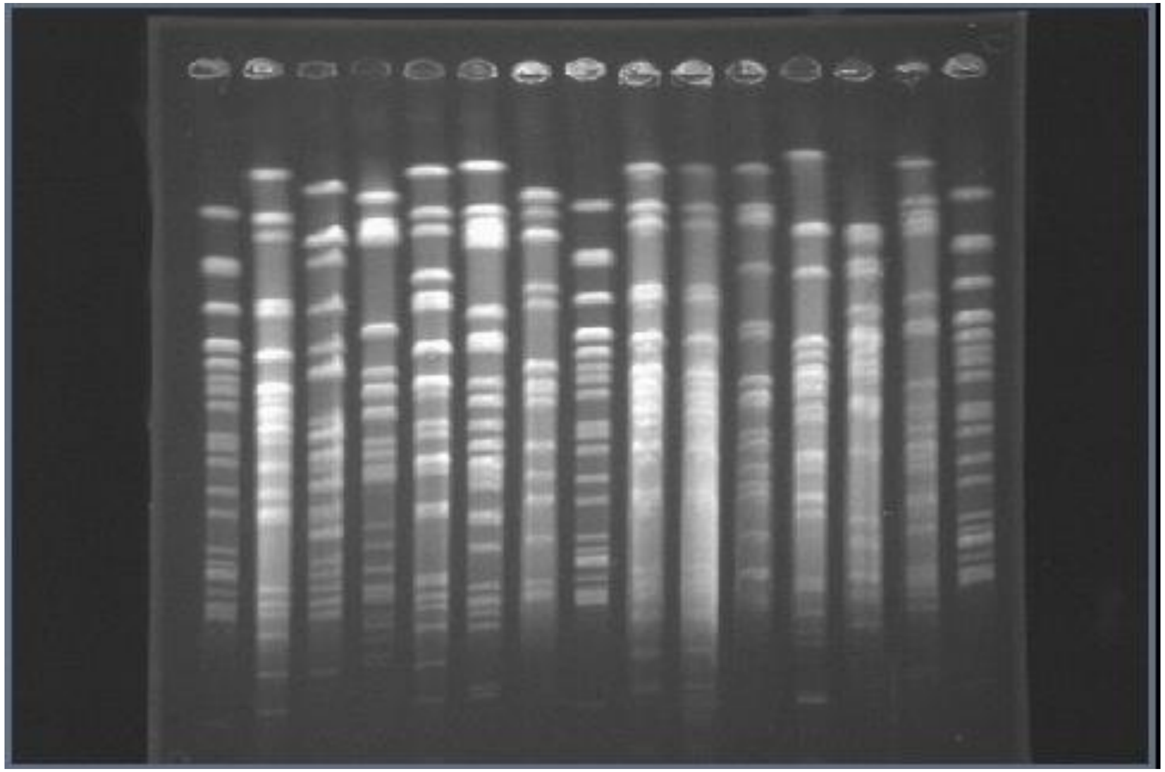

Gel 17

From the right to the left

1. G5244
2. 517
3. 518
4. 527
5. 521
6. 582
7. 586
8. G5244
9. 587
10. 598
11. 606
12. 609
13. 612
14. 613
15. G5244

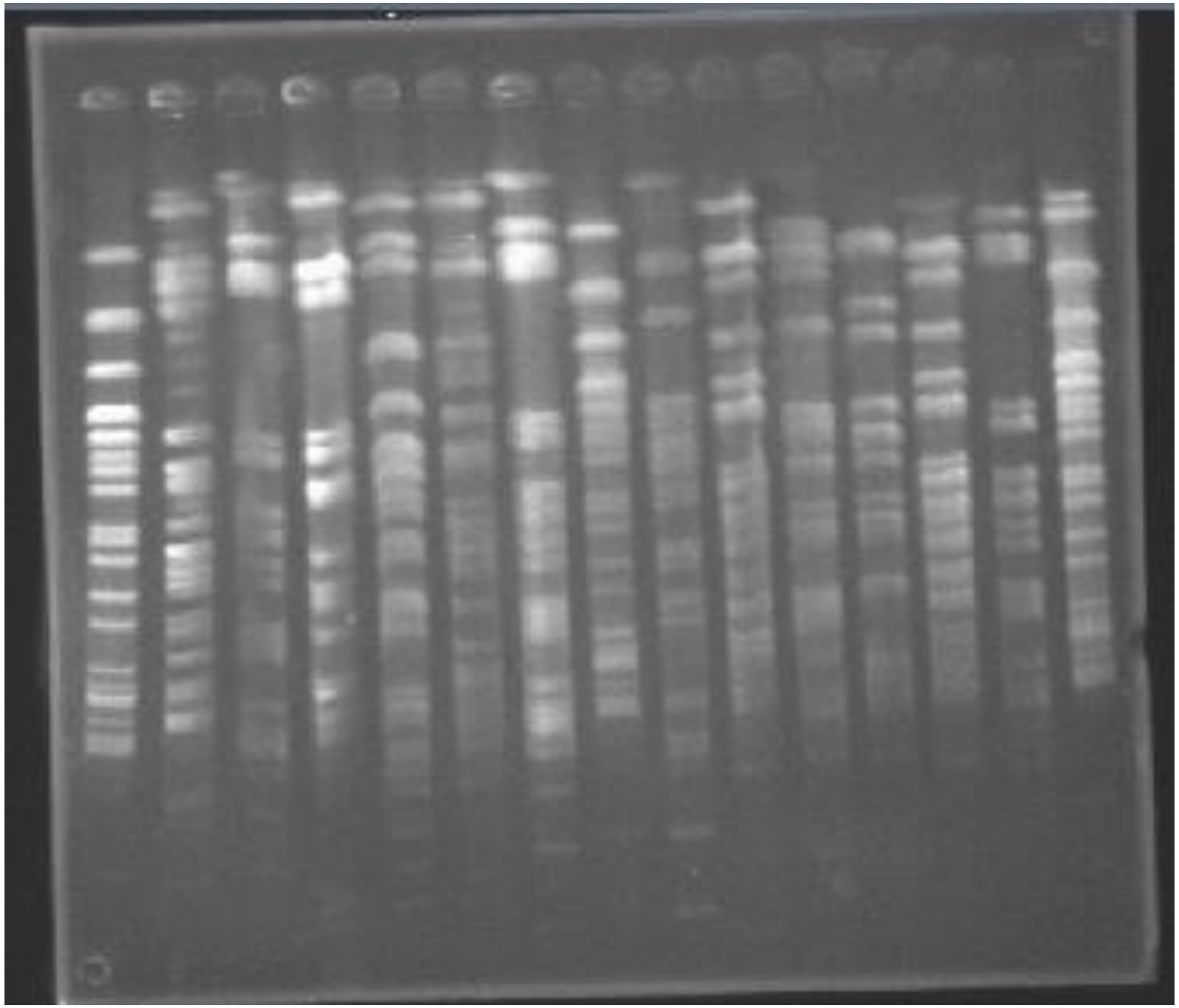

Gel 18, from the right to the left

1. G5244
2. 631X
3. 644
4. 623
5. 624
6. 658
7. 656
8. G5244
9. 659
10. 626
11. 601
12. 532
13. X
14. X
15. G5244

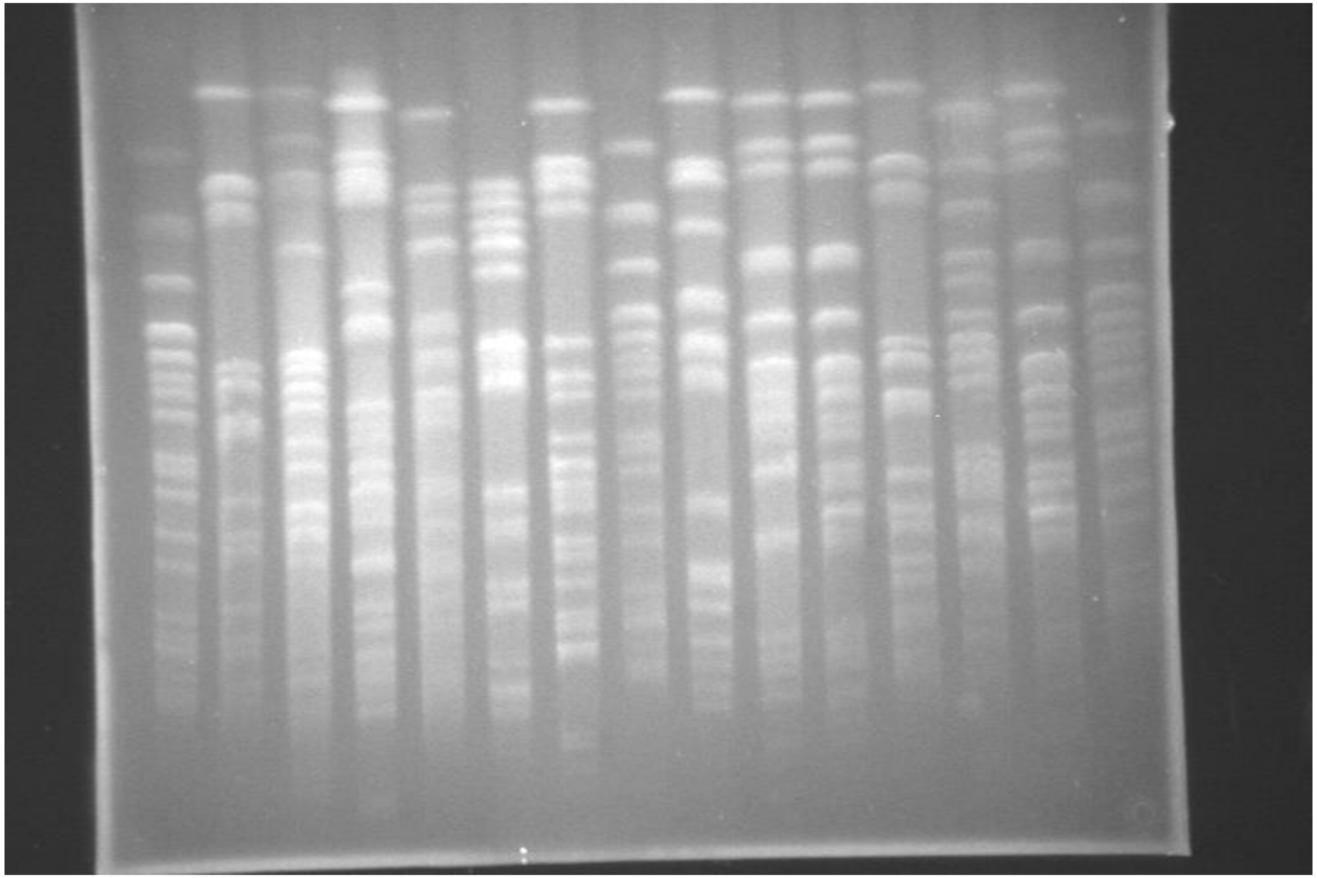

Gel 19, from the right to the left

1. G5244
2. 576
3. 645
4. 657
5. 622
6. 666X
7. 669X
8. G5244
9. 671
10. 672
11. 676
12. 683
13. 681
14. 687
15. G5244

PCR SYBR-GREEN followed by digestion with BtsCl. Almost all the gels were pictured using GelDoc and some using my phone

The lader was used in some gels 19-1114 bp.

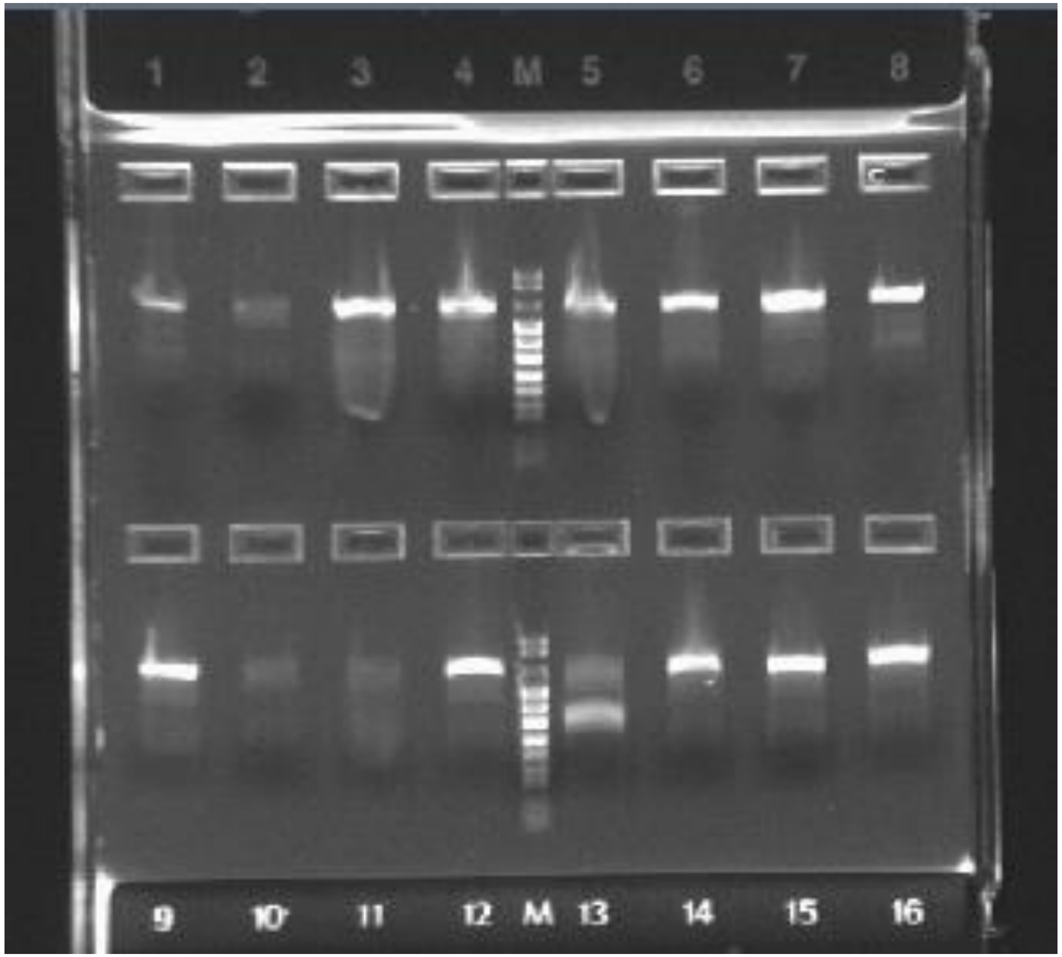

Gel 1

1. Control CR-Variant
2. X Negatve
3. 228
4. 244
5. 245
6. 249
7. 251
8. 255
9. Control CR-Variant
10. X Negatve
11. X Negatve
12. 263
13. 269
14. 272
15. 276
16. 278

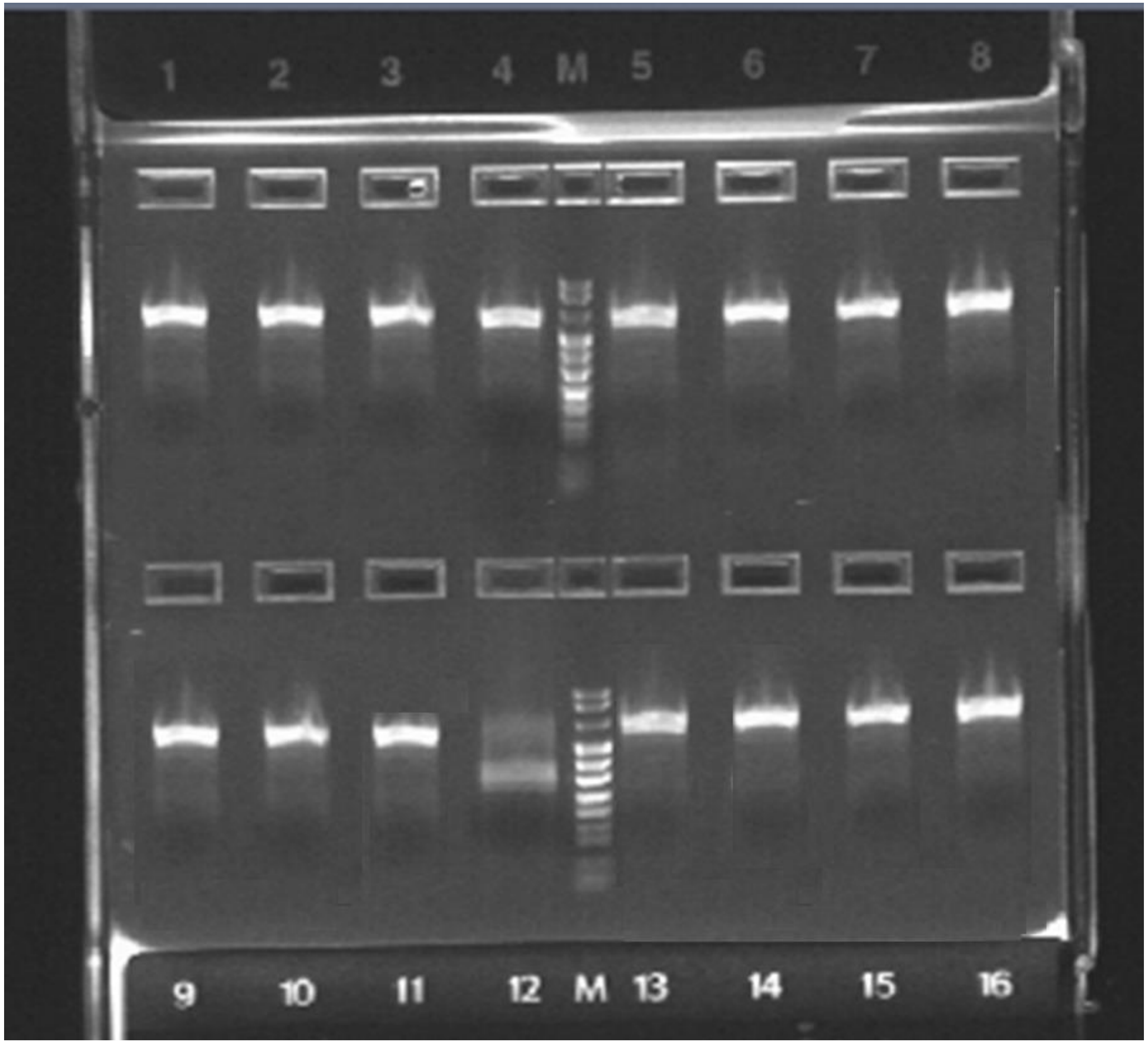

Gel 2 retouché

1. Control CR-Variant
2. 283
3. 287
4. 302
5. 304
6. 306
7. 316
8. Control CR-Variant
9. 336
10. 324
11. 322
12. 320
13. 337
14. 341
15. 342
16. 343

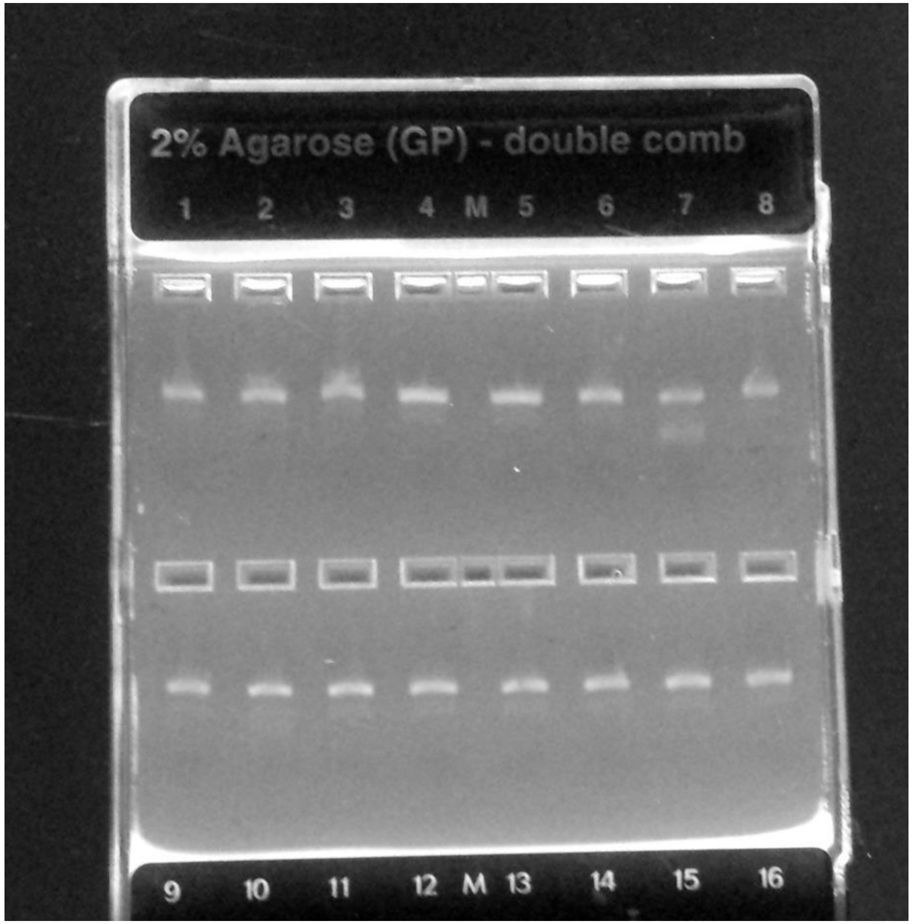

Gel 3

1. Control CR-Variant
2. 344
3. 345
4. 346
5. 347
6. 348
7. 355
8. 366
9. Control CR-Variant
10. 369
11. 375
12. 377
13. 379
14. 380
15. 382
16. 385

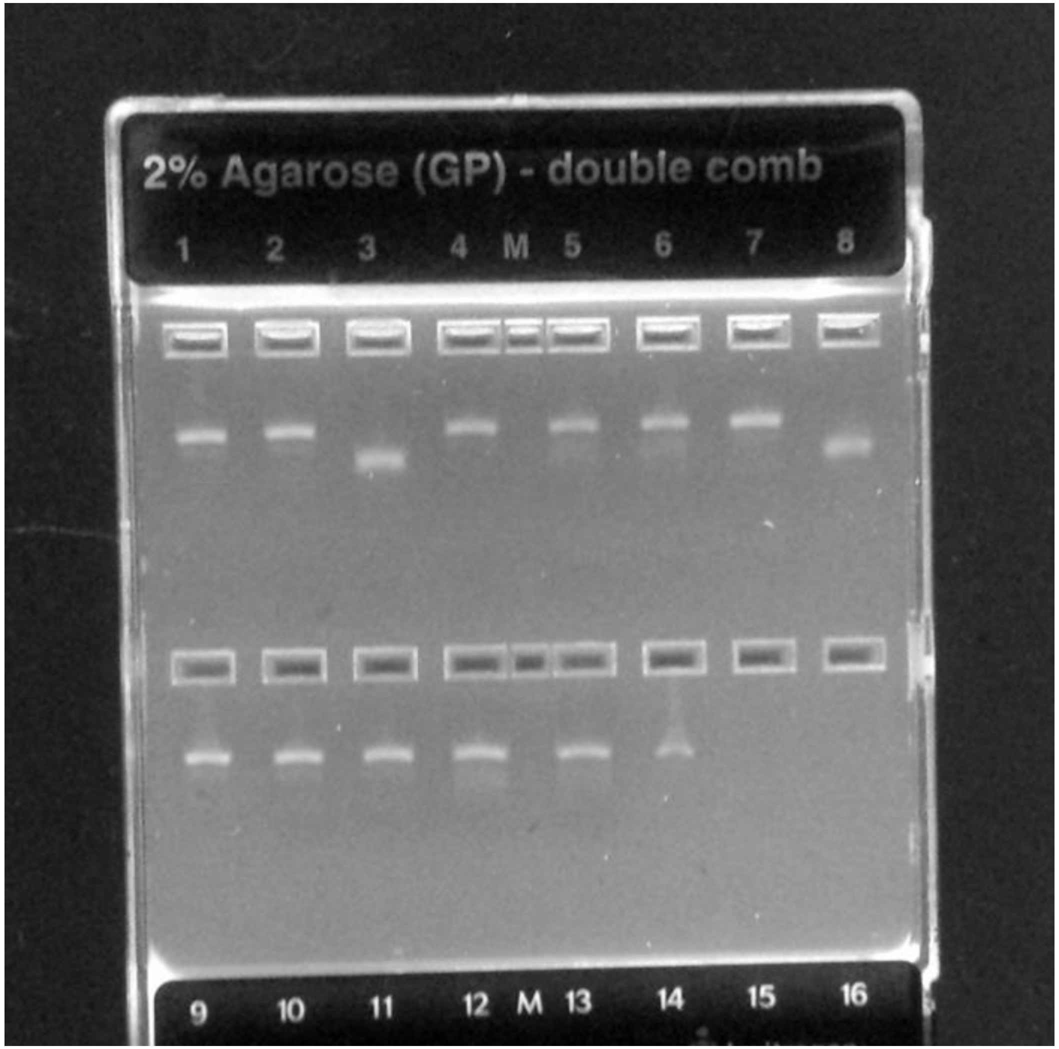

Gel 4

1. Control CR-Variant
2. 387
3. 400
4. 404
5. 408
6. 412
7. 416
8. 417
9. Control CR-Variant
10. 406
11. 429
12. 430
13. 431
14. 435
15. X
16. X

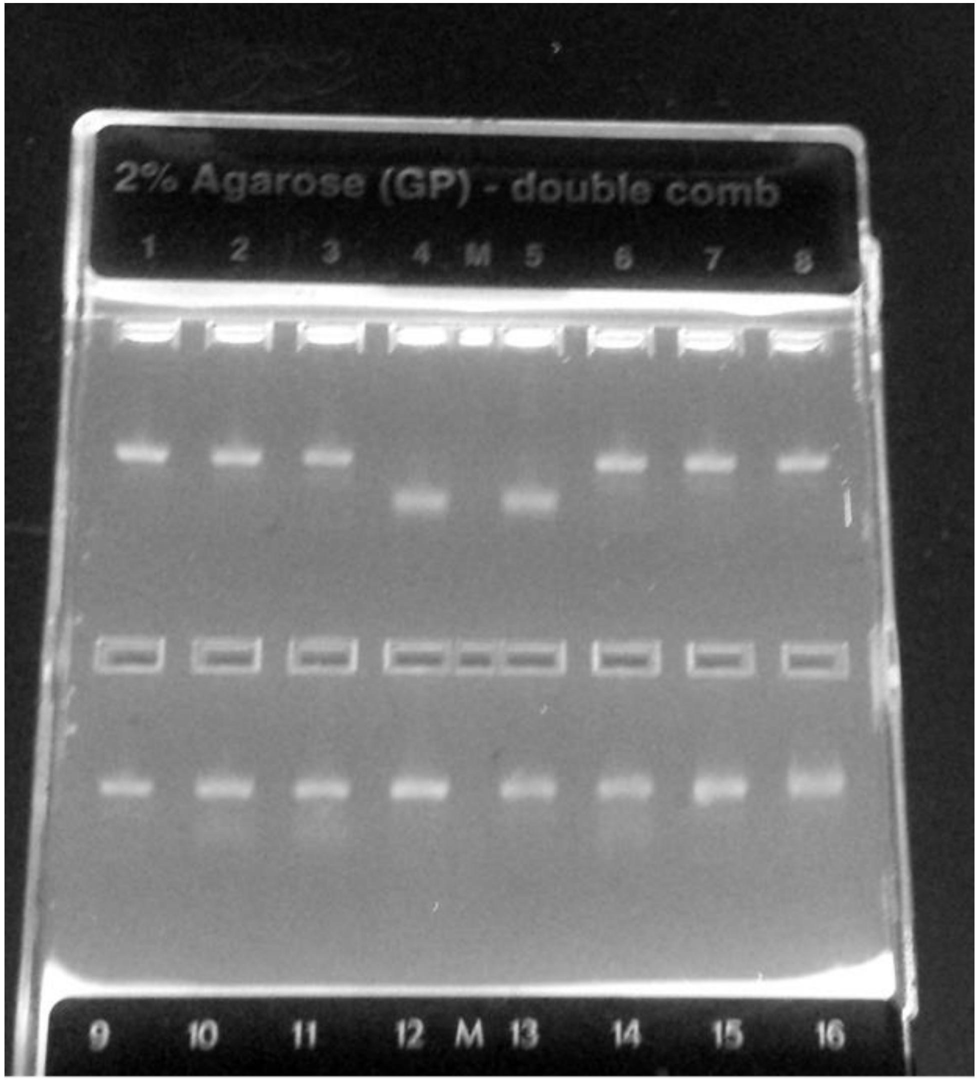

Gel 5

1. Control CR-Variant
2. 486
3. 494
4. 498
5. 500
6. 517
7. 518
8. 523
9. Control CR-Variant
10. 526
11. 527
12. 529
13. 532
14. 533
15. 544
16. 555

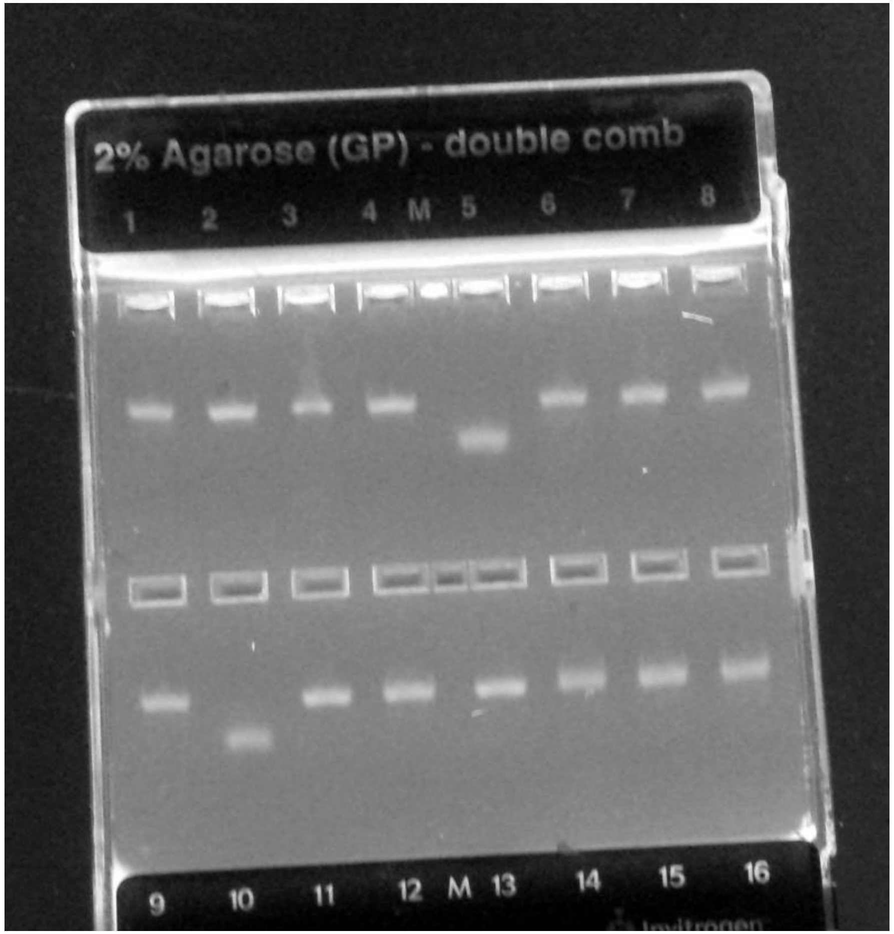

Gel 6

1. Control CR-Variant
2. 546
3. 557
4. 561
5. 571
6. 573
7. 574
8. 576
9. Control CR-Variant
10. 616
11. 613
12. 612
13. 606
14. 598
15. 586
16. 582

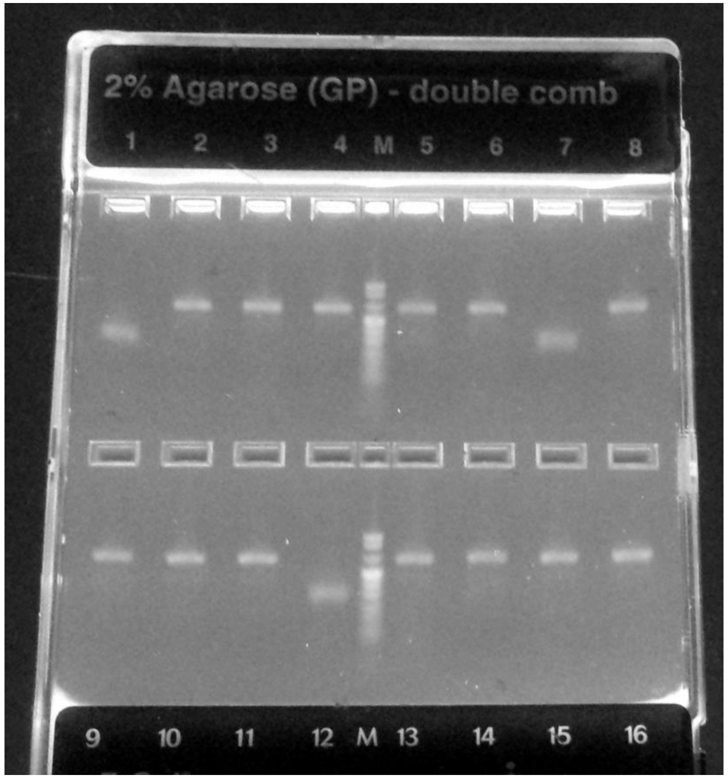

Gel 7

1. 643
2. 644
3. 645
4. 649
5. 651
6. 688
7. 690
8. Control CR-Variant
9. 656
10. 691
11. 692
12. 693
13. 694
14. 695
15. 700
16. Control CR-Variant

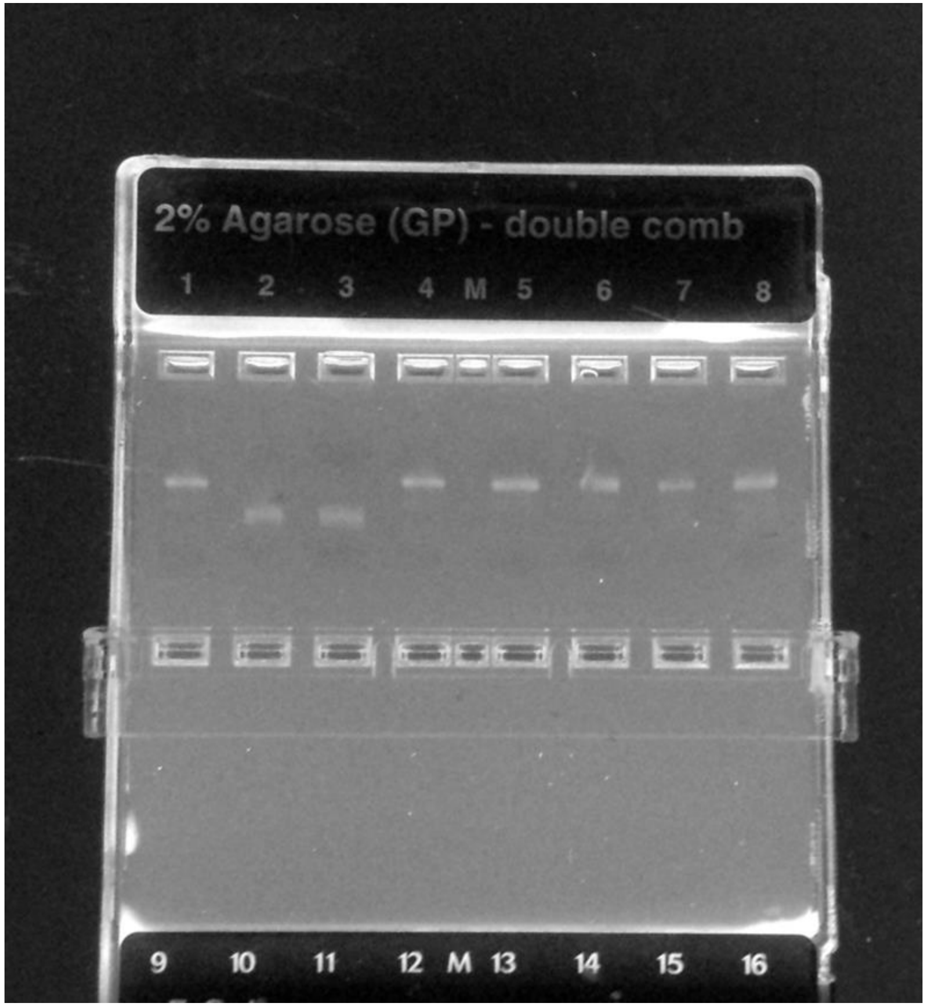

Gel 8

1. 701
2. 698
3. 697
4. 689
5. 687
6. 684
7. 683
8. Control CR-Variant

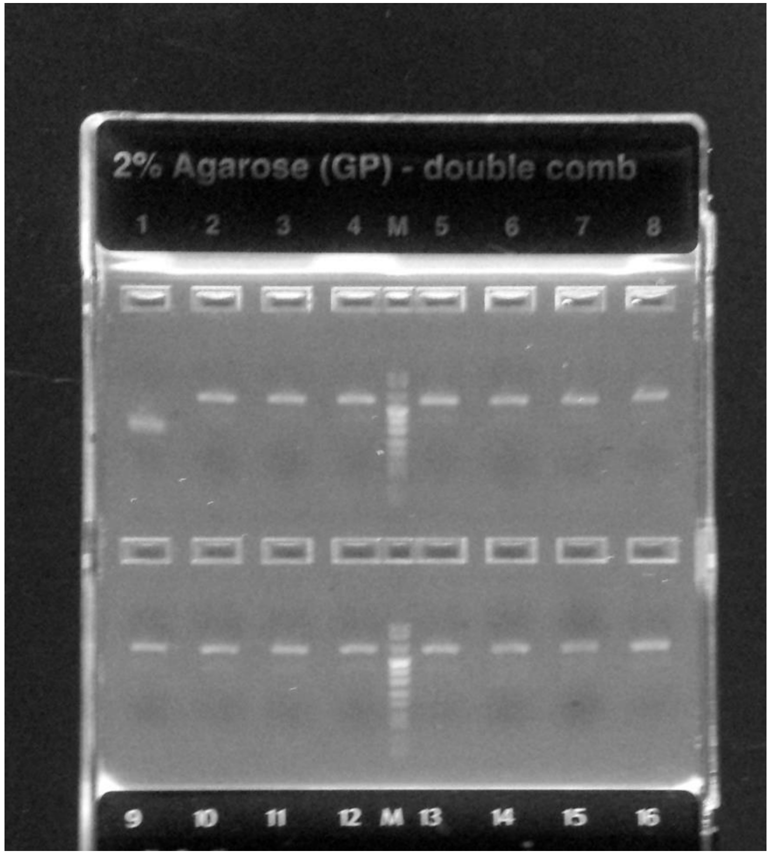

Gel 9

1. 643
2. 657
3. 658
4. 659
5. 660
6. 661
7. 662
8. Control CR-Variant
9. 663
10. 665
11. 666
12. 669
13. 670
14. 671
15. 672
16. Control CR-Variant

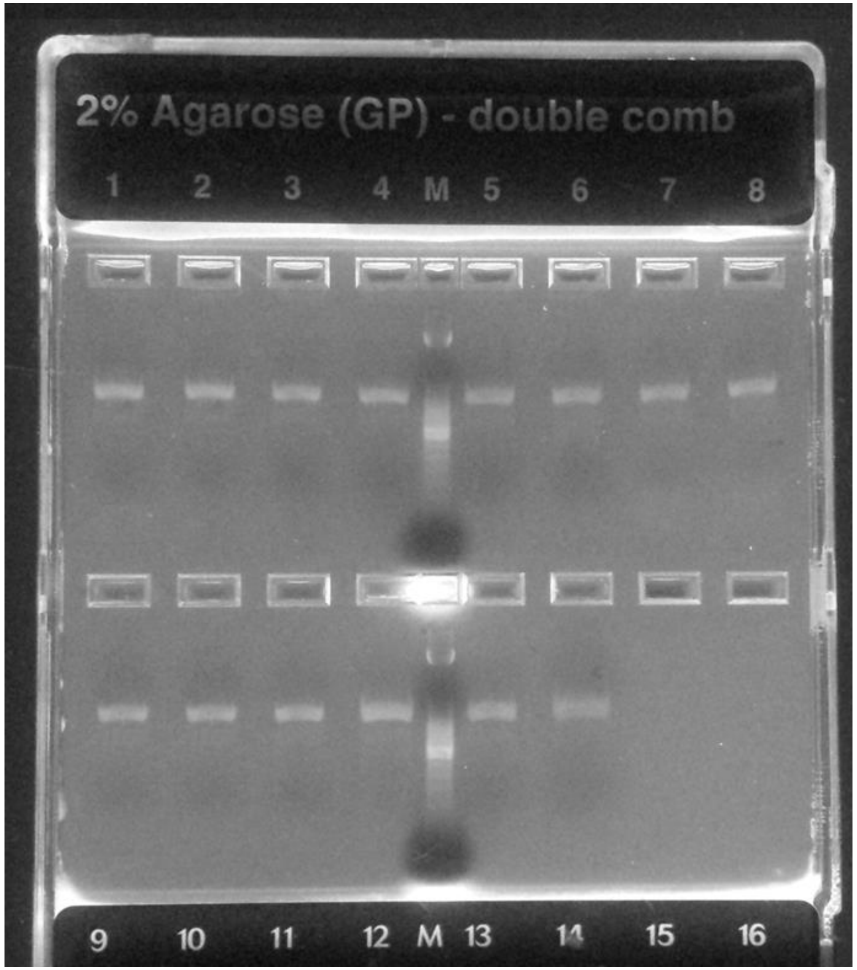

Gel 10

1. Control CR-Variant
2. 674
3. 677
4. 678
5. 679
6. 680
7. 681
8. 683
9. Control CR-Variant
10. 685
11. 687
12. 622
13. 633
14. 642
15. X
16. X

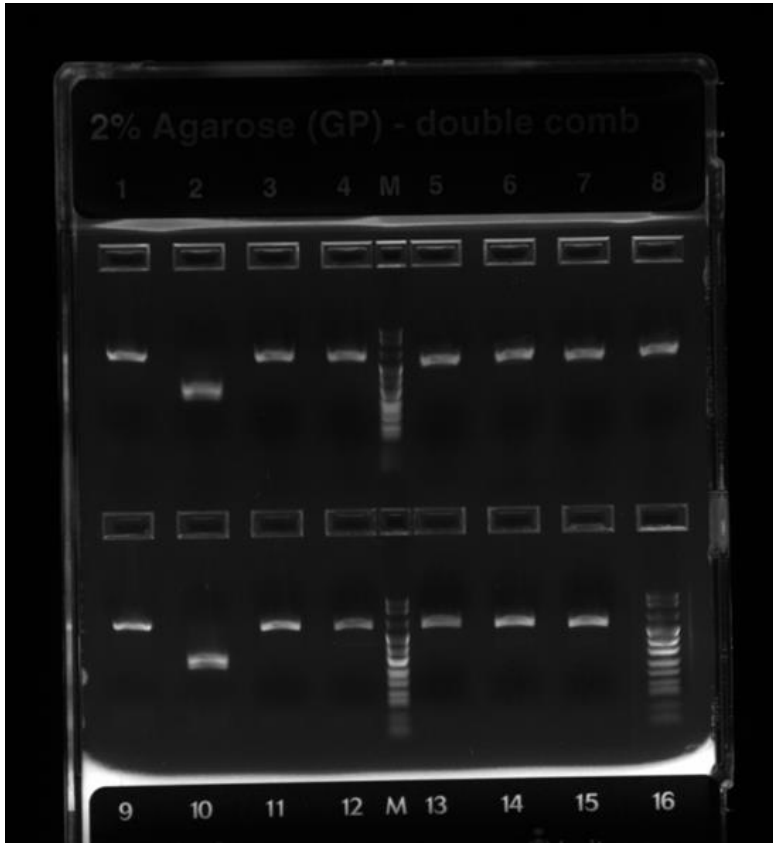

Gel 11

1. Control CR-Variant
2. Internal control aac6Ib
3. 624
4. 626
5. 628
6. 629
7. 630
8. 631
9. Control CR-Variant
10. Internal control aac6Ib
11. 632
12. 633
13. 634
14. 636
15. 642
16. Lader 19-1114 bp
