# Supplementary material for: High-risk clones of extended-spectrum β-lactamase-producing Klebsiella pneumoniae isolated from the University Hospital Establishment of Oran, Algeria (2011–2012)
Source: PLoS One. 2021 Jul 26;16(7):e0254805. doi: 10.1371/journal.pone.0254805 (PMC8312963; doi:10.1371/journal.pone.0254805)
Supplement: Supplementary file 7 [file pone.0254805.s002.docx]

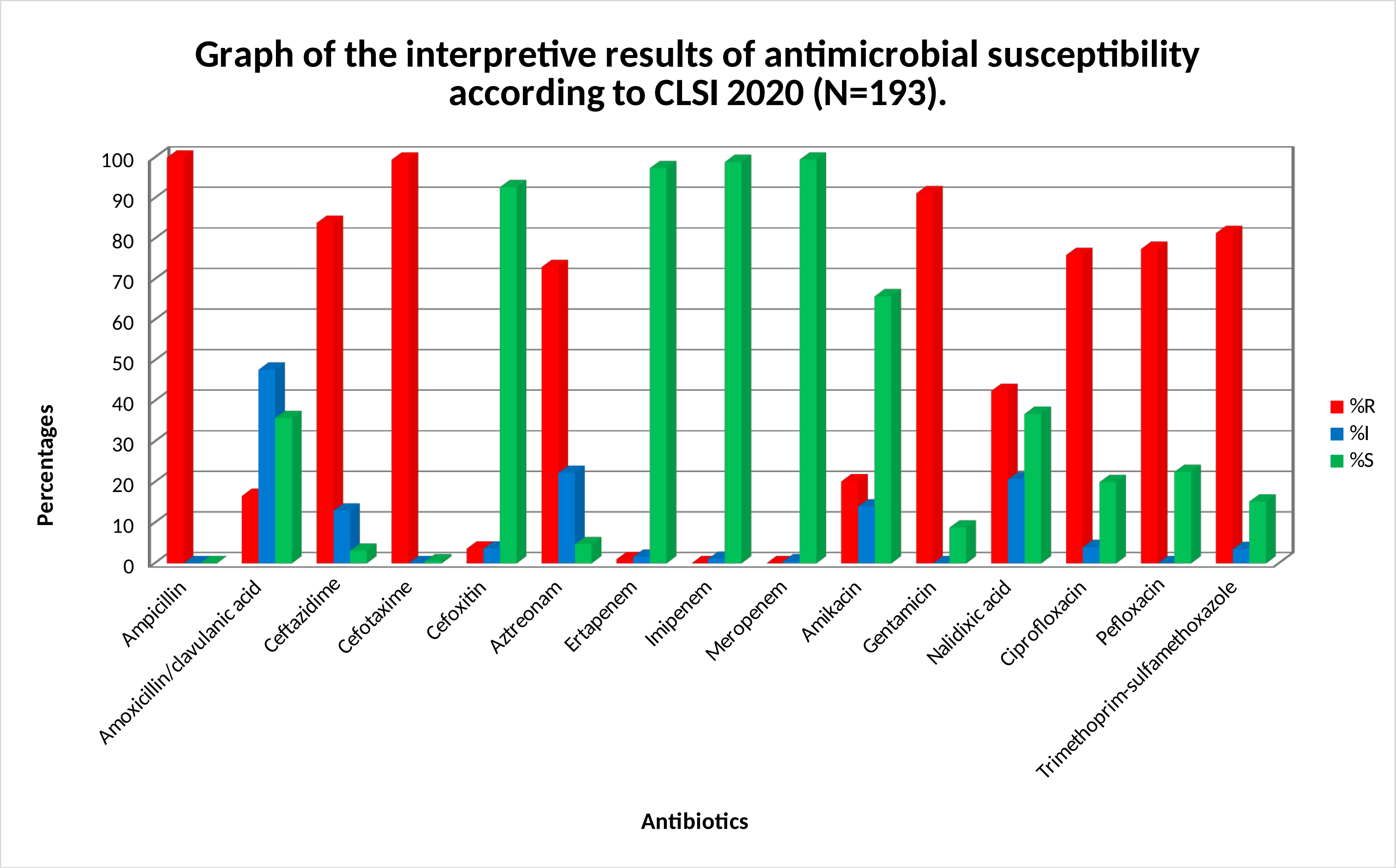

Graph of the interpretive results of antimicrobial susceptibility according to CLSI 2020 (N=193).
Percentages
0
10
20
30
40
50
60
70
80
90
100
Ampicillin
Amoxicillin/clavulanic acid
Ceftazidime
Cefotaxime
Cefoxitin
Aztreonam
Ertapenem
Imipenem
Meropenem
Amikacin
Gentamicin
Nalidixic acid
Ciprofloxacin
Pefloxacin
Trimethoprim-sulfamethoxazole
Antibiotics
%R
%I
%S
